# Reactive Passivation of Wide-Bandgap Organic–Inorganic Perovskites with Benzylamine

**DOI:** 10.1021/jacs.4c06659

**Published:** 2024-09-30

**Authors:** Suer Zhou, Benjamin M. Gallant, Junxiang Zhang, Yangwei Shi, Joel Smith, James N. Drysdale, Pattarawadee Therdkatanyuphong, Margherita Taddei, Declan P. McCarthy, Stephen Barlow, Rachel C. Kilbride, Akash Dasgupta, Ashley R. Marshall, Jian Wang, Dominik J. Kubicki, David S. Ginger, Seth R. Marder, Henry J. Snaith

**Affiliations:** † Department of Physics, Clarendon Laboratory, University of Oxford Parks Road, Oxford OX1 3PU, U.K.; ‡ School of Chemistry, Molecular Sciences Building, University of Birmingham, Birmingham B15 2TT, U.K.; § Renewable and Sustainable Energy Institute, University of Colorado Boulder, Boulder, Colorado 80303, United States; ∥ Department of Chemistry, University of Washington, Seattle, Washington 98195-1700, United States; ⊥ Molecular Engineering & Sciences Institute, University of Washington, Seattle, Washington 98195-1700, United States; # Department of Chemical and Biological Engineering, Department of Chemistry, and Materials Science and Engineering Program, University of Colorado Boulder, Boulder, Colorado 80309, United States; ¶ Department of Materials Science and Engineering, School of Molecular Science and Engineering, Vidyasirimedhi Institute of Science and Technology, Wangchan, Rayong 21210, Thailand; ∇ Department of Chemistry, Brook Hill, The University of Sheffield, Dainton Building, Sheffield S3 7HF, U.K.

## Abstract

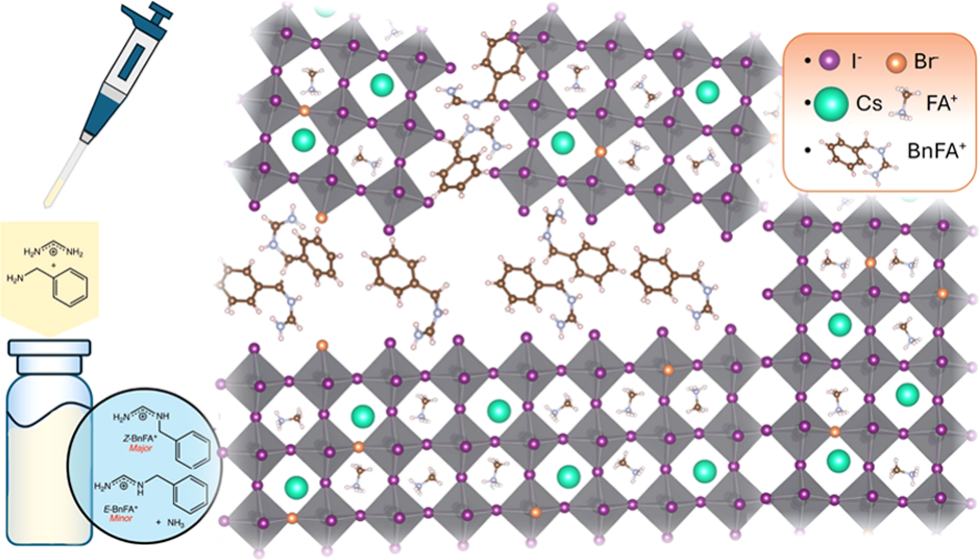

While amines are widely used as additives in metal-halide perovskites, our understanding of the way amines in perovskite precursor solutions impact the resultant perovskite film is still limited. In this paper, we explore the multiple effects of benzylamine (BnAm), also referred to as phenylmethylamine, used to passivate both FA_0.75_Cs_0.25_Pb(I_0.8_Br_0.2_)_3_ and FA_0.8_Cs_0.2_PbI_3_ perovskite compositions. We show that, unlike benzylammonium (BnA^+^) halide salts, BnAm reacts rapidly with the formamidinium (FA^+^) cation, forming new chemical products in solution and these products passivate the perovskite crystal domains when processed into a thin film. In addition, when BnAm is used as a bulk additive, the average perovskite solar cell maximum power point tracked efficiency (for 30 s) increased to 19.3% compared to the control devices 16.8% for a 1.68 eV perovskite. Under combined full spectrum simulated sunlight and 65 °C temperature, the devices maintained a better *T*_80_ stability of close to 2500 h while the control devices have *T*_80_ stabilities of <100 h. We obtained similar results when presynthesizing the product BnFAI and adding it directly into the perovskite precursor solution. These findings highlight the mechanistic differences between amine and ammonium salt passivation, enabling the rational design of molecular strategies to improve the material quality and device performance of metal-halide perovskites.

## Introduction

Organic–inorganic metal-halide perovskites (MHP) have drawn tremendous attention due to their potential applications in optoelectronics.^1^ Single-junction metal-halide perovskite solar cells (PSCs) have been extensively investigated, with power conversion efficiencies (PCEs) increasing from 3.8% to a current record efficiency of 26.7%.^2,3^ Furthermore, the tunable bandgap of perovskites allows them to be combined with silicon solar-cell technology to fabricate perovskite-on-silicon tandem photovoltaics, which are capable of yielding PCEs of over 33%.^2^ To maximize performance, a two-terminal perovskite-on-silicon tandem cell requires a top cell with a band gap of between 1.65 and 1.70 eV.^4^ Mixed-cation MHPs have been employed as highly effective photoabsorbers in such top cells.^5−7^ However, these mixed-cation, mixed-halide perovskites suffer from light-induced degradation pathways. In-operando ion migration, including halide segregation,^8^ leads to reduced open-circuit voltage and photocurrent over time.^9^ This phenomenon is due to charge-carrier funnelling from higher bandgap to lower bandgap regions resulting in charge extraction losses.^10,11^ Halide migration is likely a defect-driven process;^12,13^ hence, passivating these defects becomes crucial for reducing nonradiative recombination^14^ and slowing down halide segregation.^15,16^ Furthermore, degradation to photoinactive “non-perovskite” phases can reduce photoabsorption and inhibit charge-carrier extraction, leading to reduced photocurrent and device fill factor (FF).^11,17^ It is therefore crucial to suppress or eliminate sources of instability in wider bandgap PSCs.

Additives can be passivation agents or can lead to the formation of mixed-dimensionality systems or “hollow” 3D perovskites.^18−20^ In particular, amines in both gaseous and liquid states have been employed to passivate defects in MHPs, with enhancements to both photoluminescence and stability.^21−26^ These enhancements have been attributed to improvements in the degree of MHP preferred crystalline orientation,^23^ reduced trap densities,^24^ and altered grain size.^25−27^ NH_2_ groups can coordinate with Pb^2+^ and assist in the dissolution of PbI_2_/PbBr_2_.^28^ Kerner et al. have found that this coordination can lead to acid–base reactions between amine-PbI_2_ complexes and uncoordinated alkylamines, slowly producing (alkylamino)lead species and alkylammonium halides.^29^ As a result, many reports of amine “surface passivation” of the perovskite are likely due to dissolution of the surface layer followed by recrystallization. Amines may also react in situ with formamidinium (FA^+^),^30−34^ although most such reports employing amine additives have failed to recognize and investigate this.

We have recently shown that reactions occur in perovskite precursor solutions between FA^+^ and both the diamines ethylenediamine (EDA) and methylenediamine (MDA), forming imidazolinium and tetrahydrotriazinium, respectively.^35,36^ The presence of these reaction products is responsible for the enhanced properties of the resulting MHP materials, including homogenization of mixed-halide perovskite films, enhanced device performance, and improved photoabsorber phase stability. The new organic species are formed in solution and are incorporated throughout the processed film. In the case of EDA addition, we have shown that an excess of the additive leads to secondary phase formation.

Amines incorporating aryl groups have already been used for surface passivation in MHPs, notably by Jiang et al., who found that 3-(aminomethyl)pyridine reacts in situ with FA^+^ via an addition–elimination reaction to form *N*-(3-pyridylmethyl) formamidinium, which can act as a surface passivation layer for MHP films.^37^ Similarly, there have been reports of applying phenylalkylamines for surface passivation.^38,39^ Wang et al. have investigated the passivation efficacy of phenylalkylamines with different alkylamine chain lengths (aniline, benzylamine, and phenylethylamine). They found that benzylamine was the best passivator, which they attributed to its alkyl chain length allowing for parallel stacking benzene rings on the perovskite surface.^39^ Zhou et al. have also investigated benzylamine surface passivation with a FA_0.15_Cs_0.85_Pb(I_0.73_Br_0.27_)_3_ perovskite composition. They attributed the impact of benzylamine on device performance and moisture stability to the formation of benzylammonium lead iodide (BnA_2_PbI_4_).^38^ The formation of BnA_2_PbI_4_ would require H^+^ transfer from FA^+^ in situ during the surface processing, inconsistent with the expected amine–amidinium reactivity. As we show here, the XRD signatures reported by Zhou et al. correspond closely to phases associated with more complex benzylated species which may be forming in situ. Moreover, the morphology and diffraction measurements reported show that the additive species do not incorporate into the 3D perovskite structure, instead forming isolated domains of lower dimensionality materials, in contrast to the mechanism identified in this work. Zheng et al.^23^ have reported using linear alkyl amines and aryl amines as additives to passivate a Cs_0.05_(FA_0.92_MA_0.08_)_0.95_Pb(I_0.92_Br_0.08_)_3_ perovskite. They attribute the improved grain orientation and reduced trap states to these ligands anchoring onto the perovskite A-sites on the grain boundaries and the mechanism in which the amines “anchor” onto the perovskite A-site vacancies is thought to be amine ligating to the Pb^2+^, with amine–amidinium reactivity not considered. Not only do few reports recognize this important reaction, but also there are few reports on the synthesis and isolation of pure *N*-substituted or *N,N′*-disubstituted formamidinium halides.^40^ While these reports provide valuable insight into arylamine passivation, the complex, multimodal mechanism by which this important class of MHP additives interacts structurally with the perovskite host material—and how this interaction imparts improved optoelectronic properties to that material—remains poorly understood. Here we report the use of BnAm as an additive to improve the structural and optoelectronic properties of MHPs with bandgaps suitable for silicon-on-perovskite tandem photovoltaics. Although several other reports have focused on the use of BnAm as a passivator, in contrast, in this study we specifically find that the use of BnAm as an additive in the perovskite precursor solution leads to in situ reaction with FA^+^ to form *N*-benzyl formamidinium (BnFA^+^). By an in-depth structural study combining solid-state nuclear magnetic resonance (NMR) spectroscopy, nuclear quadrupole resonance (NQR) spectroscopy, mass spectrometry, and X-ray diffraction (XRD), we show that BnFA^+^ interacts directly with the 3D MHP structure. We propose that the only mechanism for such structural incorporation compatible with all these data is that BnFA^+^ binds to the surface of 3D MHP domains, passivating point defects and altering the structural properties of interfacial domain boundaries. By incorporating BnFA^+^ into the MHP photoactive layer in this way, we reduce nonradiative recombination, suppress halide segregation, and enhance the long-term stability of perovskite thin films and devices. To support our structural studies, we synthesize two *N*-substituted benzyl formamidinium derivatives, paving the way for targeted future investigation into these new materials. Finally, we show the implications of BnAm passivation on the long-term stability of PSCs under elevated temperature and light soaking.

## Results and Discussion

### Optoelectronic Characterization of Benzylamine Additive Perovskite Thin Films

**Figure 1:**
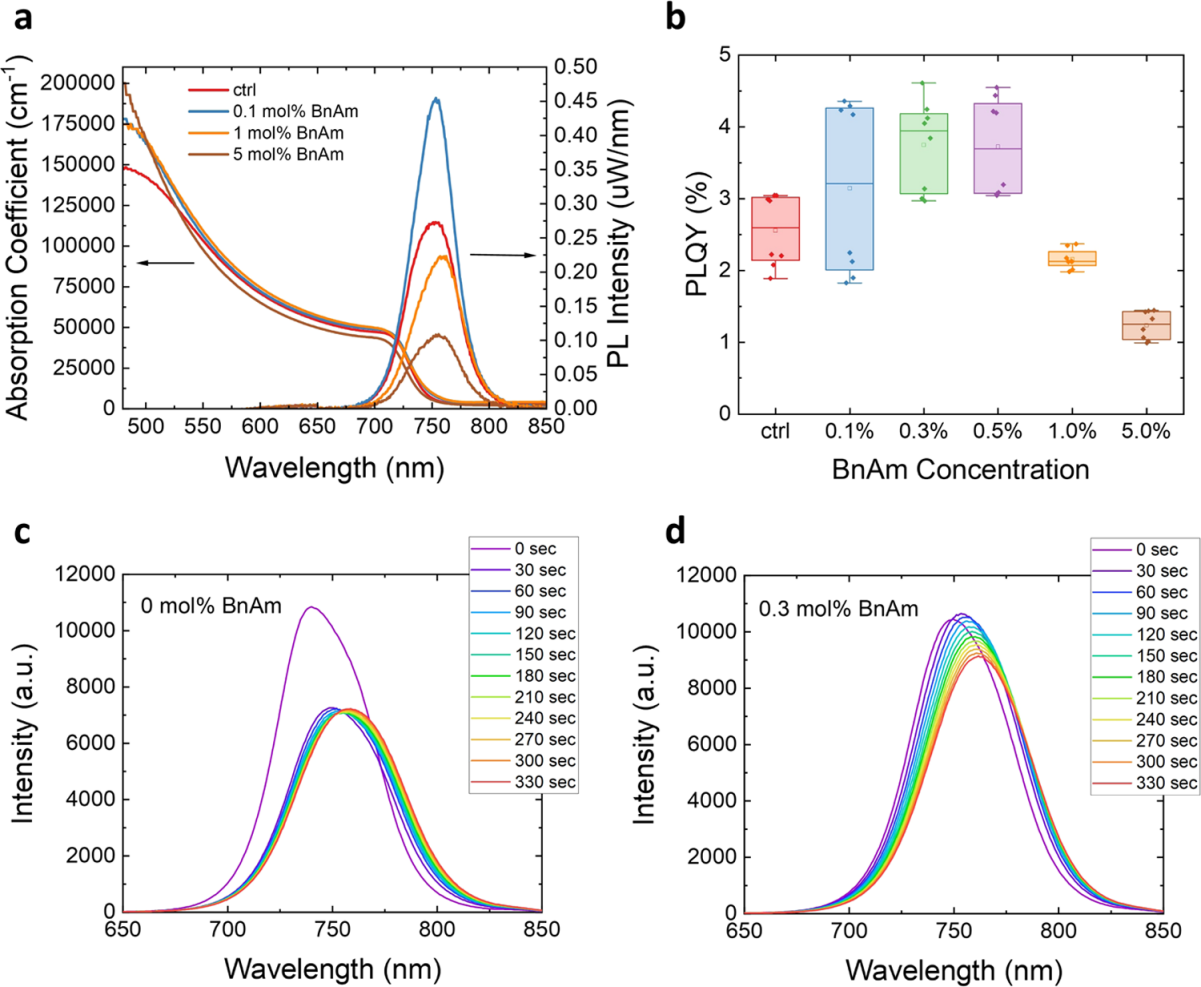
(a) UV–vis absorption spectra (left) and PL emission spectra (right) of FA_0.75_Cs_0.25_Pb(I_0.8_Br_0.2_)_3_ with 0.1–5.0 mol % BnAm additive. (b) Statistical box plot of photoluminescence quantum yield (PLQY) of FA_0.75_Cs_0.25_Pb(I_0.8_Br_0.2_)_3_ with 0.1–5.0 mol % BnAm additive measured with a 532 nm laser at 100 mW/cm^2^ (AM 1.5) intensity. Four points were measured on each film. (c) The PL spectra of the reference sample over time. (d) The PL spectra of perovskite with 0.3 mol % BnAm additive over time. Samples were measured with a 532 nm laser under 1300 mW/cm^2^ intensity.

We start by investigating the impact of adding BnAm as a bulk additive to the perovskite precursor solution for a 1.68 eV perovskite composition, FA_0.75_Cs_0.25_Pb(I_0.8_Br_0.2_)_3_, in the range of 0–5 mol % (with respect to the lead ions). In Figure 1a we show UV–vis absorbance and photoluminescence (PL) spectra of these thin films. Alongside the PL measurements, we also measure the PL quantum yield (PLQY) of the thin films under a 532 nm laser at 1 sun intensity (53.83 mW/cm^2^ for this 1.68 eV bandgap perovskite, Figure S2). We find that the luminescence efficiency is maximized when 0.3 mol % BnAm is added to the perovskite precursor solution. While the PL peak position is slightly red-shifted when BnAm is added, the optical bandgap (extracted via Tauc analysis, Figure S3) remains similar. PL imaging, which we show in Figure S4a, displays a slight enhancement in the intensity and corresponding estimated quasi-Fermi level splitting (QFLS) for compositions with up to 0.3 mol % of BnAm additive. However, consistent with our PLQY measurements, ≥1.0 mol % BnAm substantially reduces QFLS (Figure S4b).

Following the promising enhancement in PLQY with low BnAm content, we examine the effects that BnAm additive has on charge carrier transport (diffusion) and recombination by measuring and fitting time-correlated single-photon counting transient PL. We excite the FA_0.75_Cs_0.25_Pb(I_0.8_Br_0.2_)_3_ perovskite films with varying concentrations of BnAm additive with a 405 nm pulsed laser, which has a penetration depth of 41 nm, as estimated from optical constants determined by ellipsometry measurements (see Figure S5 for details). The results are shown in Figure S7. According to the time-resolved PL (TRPL) stretched exponential fitting, the average charge carrier lifetimes increased from 243 ns for the control films, to 286 ns for the 0.1 mol % and 259 ns for the 0.3 mol % BnAm additive films. We also extract β factor values, which measure uniformity in the distribution of charge carrier lifetimes, where β = 1 represents a single exponential lifetime, and lower values of β (0 < β < 1) indicate increasing heterogeneity. Together with our PLQY measurements, the increase in PL lifetime indicates a lower density of defects with a small amount of BnAm additive. However, with higher concentrations of BnAm additive (≥1 mol %), charge carrier lifetime decreases and the β value drops, indicating increased density of nonradiative recombination centers and reduced lifetime distribution uniformity, respectively.

As discussed in the introduction, light-induced halide-segregation is a critical issue for mixed-halide perovskites. To investigate the influence of BnAm addition on halide-segregation, we record PL spectra over time under 532 nm continuous-wave (CW) laser excitation with an irradiance of 1300 mW/cm^2^. In Figure 1c,d, we present a time series of PL spectra as the MHP thin films evolve under light. Within the first 30 s of illumination, the PL of the control sample has already dropped by 33% of its initial intensity. By contrast, PL intensity from the 0.3 mol % BnAm perovskite reduces more gradually and does not drop to the same intensity after 6 min of high-irradiance laser exposure. From the PL spectra, while the control sample undergoes a significant redshift within the first 30 s, the 0.3 mol % BnAm sample has a much more gradual redshift. This trend implies that the addition of BnAm decreases the rate of halide segregation, possibly through steric hindrance retarding ion motion.^41−44^ Tan et al. have shown that when there is a mismatch in the size of A-site cations, it leads to lattice distortion, causing steric impediment which increases the activation energy barrier for iodide migration.^42^

### Reaction of Benzylamine with Formamidinium in Precursor Solution

**Figure 2:**
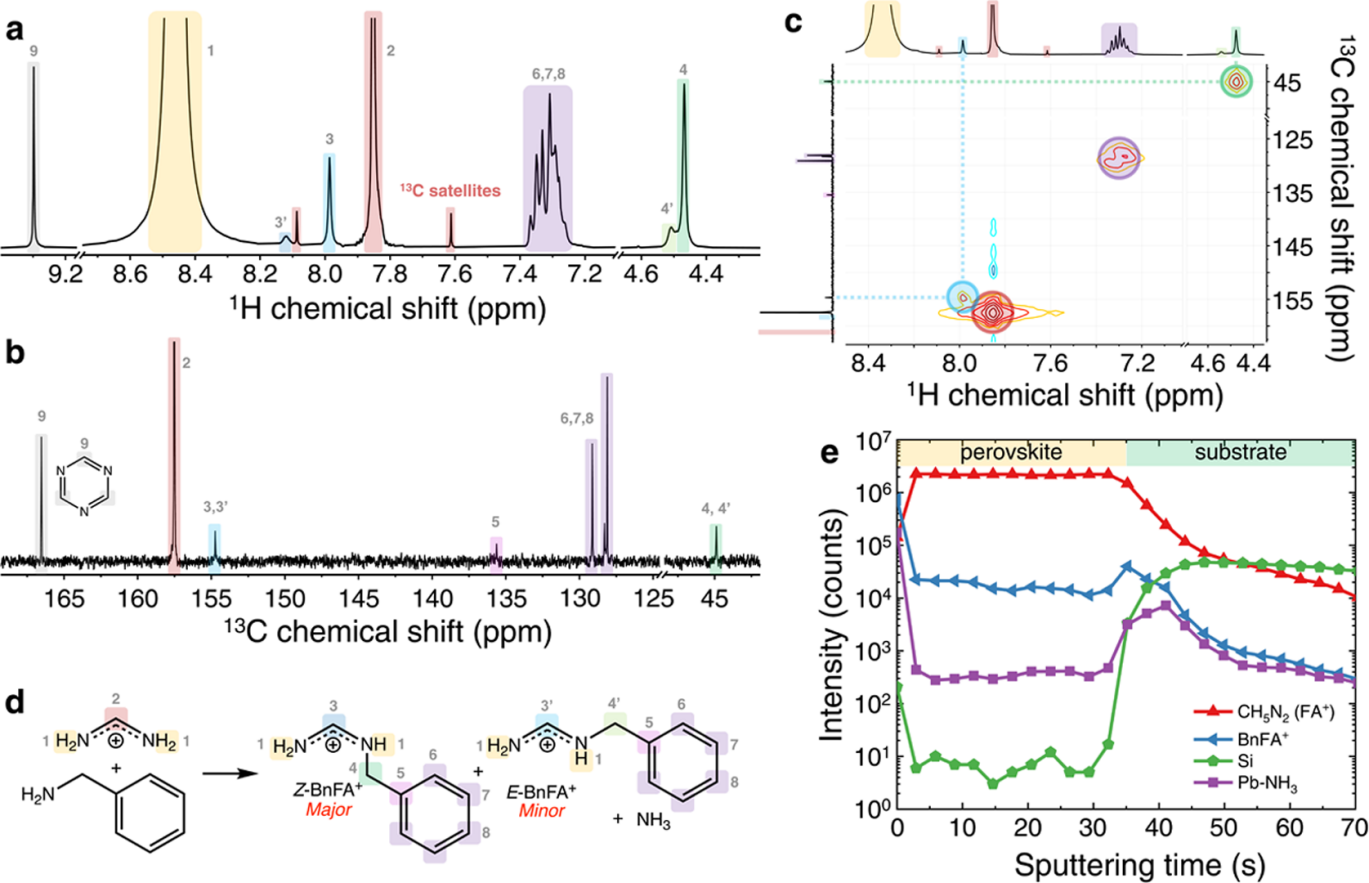
(a) ^1^H NMR spectrum of the reaction product of a BnAm and FAI mixture in DMSO-*d*_6_. (b) ^13^C{^1^H} NMR spectrum of the BnAm and FAI reaction mixture in DMSO-*d*_6_. (c) HSQC spectrum of the solution. (d) Schematic of reaction between FAI and BnAm, yielding the Z-BnFA^+^ and E-BnFA^+^ isomers. (e) ToF-SIMS molecular distribution of perovskite film with 0.6 mol % of BnAm additive. See the molecular distribution of a control perovskite film in Figure S28. Note that the Pb–NH_3_ fragment is absent in the control film.

As discussed above, many different modes of interaction and reaction of amines with perovskite materials have been reported.^29,35−37^ To examine which, if any, of these mechanisms is responsible for the enhancement we observe, we first carry out solution ^1^H NMR spectroscopy on our precursor ink to determine if there are chemical reactions between FA^+^ and BnAm in solution, identify their products and monitor the rate of their formation. Figure 2a shows the ^1^H NMR spectrum of a solution of FAI in deuterated dimethyl sulfoxide (DMSO-*d*_6_) with 5 mol % BnAm added. In Figure S11, we compare the spectra of FAI, BnAm, and benzylammonium iodide (BnAI). From this analysis, it is clear that we do not observe any BnAm in solution in Figure 2a, or its conjugate acid, BnA^+^. Instead, a mixture of FA^+^ and a new benzyl-containing organic is detected. The new species shows a singlet peak in the ^1^H NMR spectrum at a chemical shift (δ) of 7.99 ppm, close to the methine resonance of FA^+^ (7.86 ppm). Correspondingly, a ^13^C NMR spectrum, with ^1^H decoupling, {^1^H}, of the same solution shows a new signal at 154.7 ppm suggesting the presence of a new non-FA^+^ methine environment (Figure 2b). Heteronuclear single quantum correlation (HSQC) spectroscopy confirms that the two new signals described correspond to bonded nuclei (Figure 2c). These data are consistent with the formation of *N*-benzyl formamidinium (BnFA^+^) by nucleophilic attack of BnAm on FA^+^, with subsequent elimination of ammonia. To confirm that the newly formed species is BnFA^+^, we have synthesized and purified BnFAI (see Note S3.4.1). Comparison with NMR of an isolated neat sample of BnFAI (see Figures S18–S20 and 2d) confirms that the predominant in situ-formed product is indeed BnFA^+^, specifically the *Z*-isomer, and indicates that additional minor peaks in the ^1^H NMR spectrum are consistent with the *E*-isomer (Figure 2a,d). We show this reaction scheme in Figure 2d and account for it mechanistically in Figure S12. The use of higher concentrations of BnAm leads to more complex precursor solution chemistry. For <5 mol % BnAm, the substantial excess of FA^+^ in solution ensures that the only product formed in detectable quantities is BnFA^+^. However, as we show in Figure S13, when the BnAm concentration is increased to 20 mol %, we observe an additional set of signals (Figure S14) which we interpret as *N*,*N*′-dibenzyl formamidinium (Bn_2_FA^+^) formed via condensation of a second BnAm molecule with BnFA^+^ in solution. We discuss full details of this process in Figures S14–S16, and Note S3.4.2. Figure S17 shows mass spectra we acquired immediately after adding BnAm into an FAI solution, the presence of only BnFA^+^ (*m*/*z* = 135.1) and Bn_2_FA^+^ (*m*/*z* = 225.1) peaks indicate the reaction goes to completion within the time scale of mixing and injection into the mass spectrometer, i.e., on a time scale of less than 5 min.

### BnAm Bulk Additive Thin Film Structure

To understand how the BnFA^+^ affects the structure of MHPs, we first acquire time-of-flight secondary-ion mass spectrometry (ToF-SIMS) data on a FA_0.75_Cs_0.25_Pb(I_0.8_Br_0.2_)_3_ film formed using 0.6 mol % BnAm additive (Figure 2e). In this measurement, the sputtering ion beam probes deeper into the thin film over time, allowing us to determine how BnFA^+^ is distributed within the film. We find that the BnFA^+^ fragment is distributed throughout the perovskite film with a higher concentration at the bottom surface, and perhaps the top surface (the first data point in the ToF-SIMS may not be completely reliable). This distribution suggests BnFA^+^ is preferentially at the bottom interface of the film. We do not detect BnAm or BnA^+^ fragments; however, we do detect a Pb–NH_3_ signal. We do not observe any Pb–NH_3_ signal in the ToF-SIMS measurement of the control film. As noted above, NH_3_ is produced alongside BnFA^+^ as BnAm and FA^+^ react. This finding suggests that NH_3_ generated in solution may ligate Pb^2+^, altering the crystallization of the perovskite phase, and may be incorporated into the perovskite layer. According to Li et al.,^33^ ammonia gas generated during film formation affects perovskite crystallization and may even “heal” the defects in the polycrystalline perovskite films. The “healing” process increases the grain size at the expense of smaller grains which results in fewer grain boundaries and reduces grain boundary recombination. According to the scanning-electron microscopy (SEM) images and the histogram distribution of grain size in Figure S25, the BnAm additive perovskite film has a slightly larger average grain size than the control perovskite film, potentially due to the healing effects of BnAm and/or NH_3_.

Next, we conduct 1D XRD, 2D XRD, and GIWAXS on our treated perovskite films to determine the phase composition of our material. Given that BnA^+^ has been reported in 2D Ruddlesden–Popper perovskite phases of the family (BnA)_2_MA_*n*–1_Pb_*n*_X_3*n*+1_,^45,46^ we hypothesized that similar BnFA^+^ 2D perovskite phases might form in our materials. However, at low BnAm additive concentrations (<1 mol %), 1D XRD patterns show no evidence of lower-dimensionality perovskite phases (Figure S27). At higher additive concentrations (≥5 mol %) we observe the emergence of a peak at 2θ = 6.2°, consistent with lower-dimensional phase formation (Figures S29–S31 and S34). GIWAXS and XRD measurements of perovskites with 10–20 mol % BnAm (Figures S29d and S31, discussed in detail in Supporting Information Note S3.5.2), show the peak at 6.2° becomes more prominent as BnAm concentration is increased. Despite close correspondence with the *d*-spacing of BnA_2_PbI_4_, simulations suggest that no known BnA^+^ phase can account for all of the observed reflections (Figure S29g–i). The large length scale periodicity in the GIWAXS points to a distorted or step-like layered phase, which cannot be accounted for by reported BnA^+^/FA^+^ phases.^45,47,48^ Such distortions and large unit cells have been reported due to octahedral distortions by disordered organic spacer layers,^49^ and so this observation is consistent with segregation of a phase including the BnFA^+^ cation at ≥5 mol % BnAm. To confirm that these peaks do not correspond to a BnA^+^-containing phase, we fabricate perovskite films with 20 mol % excess BnAX (X = Cl^–^, Br^–^, I^–^) additive. As expected, the 1D XRD patterns of these materials do not show any new peaks compared to the pristine perovskite (Figure S31b). From these data, we conclude that at high additive concentrations, we have formed new low-dimensionality perovskite phases containing in situ generated BnFA^+^.

**Figure 3:**
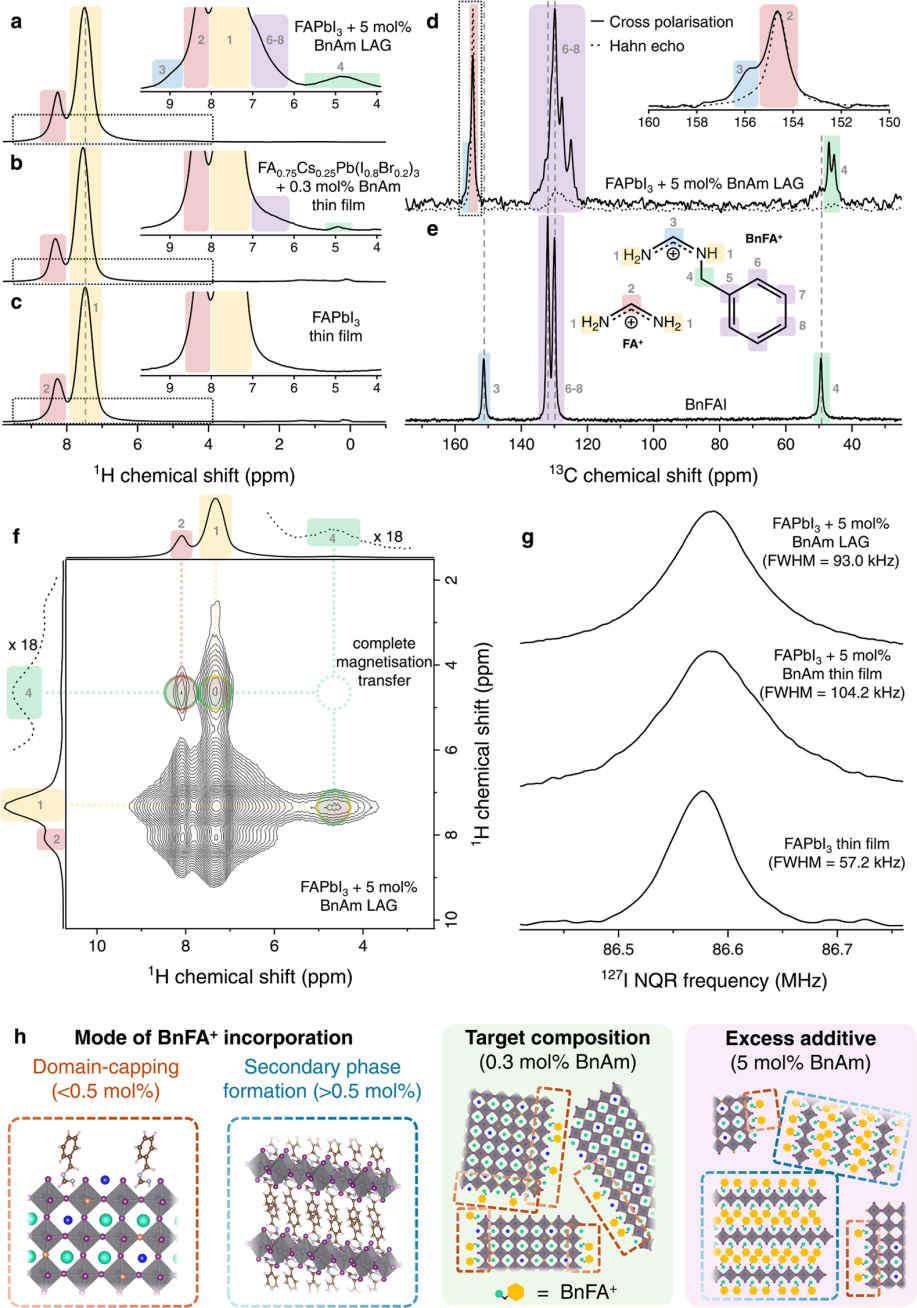
^1^H magic-angle spinning (MAS) NMR spectra of FAPbI_3_ + 5 mol % BnAm fabricated mechanosynthetically by liquid-assisted grinding (a), FA_0.75_Cs_0.25_Pb(I_0.8_Br_0.2_)_3_ + 0.3 mol % BnAm thin films (b), and FAPbI_3_ thin films (c) (23.5 T, 55 kHz). Insets highlight peaks corresponding to the BnFA^+^ additive. Spectra shown in full are quantitative. Insets are acquired using a short recycle delay to highlight the more rapidly relaxing additive nuclei. ^1^H-decoupled ^13^C MAS spectra of FAPbI_3_ + 5 mol % BnAm fabricated by liquid-assisted grinding (d) and BnFAI (e) (11.7 T, 15 kHz). Spectra were acquired either by ^1^H–^13^C cross-polarization (CP) or using a Hahn echo pulse sequence, to highlight the rigid and mobile organic species, respectively. (f) ^1^H–^1^H spin diffusion (SD) spectrum of FAPbI_3_ + 5 mol % BnAm fabricated by liquid-assisted grinding. Sections of cumulative 1D projections shown by dotted lines are enlargements of the corresponding region. (g) ^127^I nuclear quadrupole resonance (NQR) spectra of the radiofrequency region containing NQR transitions of ^127^I nuclei within α-FAPbI_3_ phase. All thin film materials are mechanically exfoliated to give a powder prior to measurement. (h) Schematics highlighting the modes of BnFA^+^ incorporation into perovskite materials (left) and the additive concentration-dependence of these incorporation modes. Molecular structures are adapted from VESTA.3 Software.^56,57^

To investigate how BnFA^+^ is incorporated in the perovskite material at both device-relevant (<1 mol %) and higher concentrations, we conduct a range of solid-state NMR (ssNMR) and nuclear quadrupole resonance (NQR) spectroscopy measurements on BnAm-derived materials fabricated as both thin films and mechanochemically produced powders. Such measurements are capable of probing the atomic level arrangement of organic species within a material. A mechanosynthetic approach to the target materials is used to generate the substantial quantity of material (>100 mg) required for some nuclear spectroscopy measurements, notably ^13^C NMR and ^127^I NQR. Mechanochemistry has been widely shown by us and others to be an effective synthetic approach to microcrystalline halide perovskites, including those making use of additive-enhancement strategies (as in this work), and to appropriately mimic polycrystalline thin film materials of the same composition. Nevertheless, we are careful to repeatedly validate that our solid-state approach replicates the same BnFA^+^-perovskite structural interaction as is observed in our solution-processed thin films. First, to establish the formation of BnFA^+^ in situ during our mechanosynthetic procedure, we fabricate α-FAPbI_3_ with 5 mol % BnAm additive by a liquid-assisted grinding method (see Experimental Methods). We choose α-FAPbI_3_ as a starting point for this investigation as more compositionally complex materials are less well ordered leading to broadening of NMR and NQR signals, which may obscure features corresponding to additives. ^1^H magic-angle spinning (MAS) NMR of this material (Figure 3a) shows ^1^H environments corresponding to BnFA^+^. In particular, we highlight the signal at 9.0 ppm (**3**), which corresponds to a methine proton only present in BnFA^+^, not BnA^+^. We note that for all perovskite materials investigated by ssNMR and NQR, we anneal the sample inside the NMR rotor at ∼150 °C immediately prior to measurement to ensure it is fully in the α-phase. Next, we compare the ^1^H spectra of our champion PLQY composition, FA_0.75_Cs_0.25_Pb(I_0.8_Br_0.2_)_3_ + 0.3 mol % BnAm (Figure 3b), and FAPbI_3_ (Figure 3c). Despite the low additive loading, we are able to resolve two signals corresponding to BnFA^+^ in our champion device composition. Quantitative ^1^H MAS NMR (Figure S35) suggests that our device composition contains ∼0.25 mol % BnFA^+^, consistent with the 0.3 mol % BnAm added. We attribute the slight shift in the position of peak 4 relative to Figure 3a (0.09 ppm) to the differing A-site and X-site composition of this material. A similar 0.07 ppm shift is observed in the peaks corresponding to FA^+^ in these materials (peaks **1** and **2**). In Figure S35 we show the ^1^H MAS spectrum of BnFAI, and the full ^1^H spectra of all materials investigated. The comparatively broad peaks of BnFAI are consistent with the denser ^1^H–^1^H dipolar coupling network in this proton-rich solid and presumably overlap of broad resonances obscures the presence of both *E* and *Z*-isomers seen in solution samples of the same material. The substantial narrowing of peak **4** in BnFA^+^ contained in the champion perovskite material compared to neat BnFAI indicates that the BnFA^+^ in the perovskite samples exhibits smaller dipolar couplings consistent with the more sparse ^1^H dipolar coupling network commonly observed in hybrid organic–inorganic phases such as perovskites.^50,51^ As we highlight in Figure S36, there is an important difference between the FAPbI_3_ + 5 mol % BnAm liquid-assisted grinding material discussed here, and the corresponding FAPbI_3_ + 5 mol % BnAm thin film material whose ^1^H MAS NMR spectrum is shown in Figure S35. At 5 mol % BnAm additive loading in the thin film material, we detect the presence of an additional peak corresponding to a more rigid BnFA^+^ environment. Based on our GIWAXS and XRD measurements of this material (Figures S29 and S30) we attribute this peak to BnFA^+^ in a 2D perovskite phase that forms only when excess BnFA^+^ is present in the material. Notably, despite the same 5 mol % additive loading, the liquid-assisted grinding material produced does not show this peak, and instead shows only the peak corresponding to BnFA^+^ in the same environment as in the device composition which can be due to the larger surface area of the liquid-assisted grinding materials.

To further corroborate the in situ formation of BnFA^+^ and its presence in the perovskite materials, we conduct ^1^H-decoupled ^13^C MAS NMR. Figure 3d shows the ^13^C MAS NMR spectra of FAPbI_3_ + 5 mol % BnAm fabricated via liquid-assisted grinding recorded using two complementary strategies. ^1^H–^13^C cross-polarization (CP) transfers ^1^H polarization to adjacent ^13^C nuclei via dipolar couplings. The efficiency of this transfer strongly depends on the degree of mobility of the species containing these nuclei. Efficient CP requires strong dipolar couplings, which is a situation typical for a rigid solid. On the other hand, rapid liquid-like reorientation of FA^+^ on the perovskite A-site removes the intramolecular dipolar couplings rendering CP in this molecular fragment inefficient.^51^ We use this effect to emphasize the presence of BnFA^+^ in the material. A ^13^C CP spectrum of the liquid-assisted grinding material shows peaks corresponding to BnFA^+^; in particular, peak **3** (155.8 ppm), which corresponds to the methine environment of BnFA^+^ and is not present in BnA^+^. Notably, we acquire this spectrum cumulatively by conducting several identical measurements sequentially to confirm that the spectrum is not evolving over time, as would be the case if α-FAPbI_3_ were gradually transforming to δ-FAPbI_3_, for example. We then immediately record a second ^13^C spectrum of the same material using an echo sequence, without applying any thermal treatment between the two experiments. The echo experiment detects all ^13^C environments regardless of their dynamics and therefore in it the FA^+^^13^C signal at 154.7 ppm is much more pronounced than in CP. By conducting this sequence of experiments we are able to confirm two important results: (1) the motional degrees of freedom of BnFA^+^ in the perovskite material are much more restricted compared to FA^+^ since the relatively strong dipolar couplings result in good CP signal for BnFA^+^, and (2) peak **3** does not correspond to FA^+^ in the degraded δ-FAPbI_3_ phase, which has been reported at 0.9 ppm higher chemical shift than the α-phase,^36^ but whose formation would evolve over time during the CP experiment and be easily detected under the Hahn echo experiment. By comparing the ^13^C CP spectrum of FAPbI_3_ + 5 mol % BnAm to that of BnFAI (Figure 3e), we show the close correspondence of the observed signals to those of BnFA^+^ in this material. The splitting of the aromatic (peaks **5–8**) and methylene (peak **4**) environments strongly suggests that there are at least two different binding modes of BnFA^+^ inside this material. A small, broad signal centered at ∼50 ppm is also visible, most likely corresponding to a small quantity of in situ formed BnFA^+^ present in a disordered and isolated phase, such as BnFAI. The two *E*/*Z* isomers may also be present, potentially in a different ratio from what is seen in the presynthesized solid and contribute to the splittings.

Having established that BnFA^+^ is present in both the best thin film composition and FAPbI_3_ derivatives, we next explore the nature of its interaction with the perovskite structure. To do so, we first conduct a ^1^H–^1^H spin diffusion experiment. This measurement relies on the transfer of magnetization within a network of dipolar-coupled ^1^H nuclei and thus can be used to establish spatial proximity between nuclei separated on the order of tens of Å.^52−54^ Observation of spin diffusion between two distinct nuclei therefore indicates atomic-level spatial proximity between those species. Importantly, it also requires those nuclei to be present within the same crystalline domain, as the small contact area between adjacent grains effectively prevents spin diffusion across the boundary.^36^Figure 3f shows a ^1^H–^1^H spin diffusion spectrum of FAPbI_3_ + 5 mol % BnAm synthesized by liquid-assisted grinding. We observe cross peaks (highlighted with solid circles) between the characteristic BnFA^+^ peak (**4**) and both the amidinium (N–^1^H, peak **1**) and methine (C–^1^H, peak **2**) peaks of FA^+^. These cross peaks provide clear evidence that the BnFA^+^ is in atomic-level contact with FA^+^ in the perovskite structure. Moreover, the nearly complete transfer of magnetization from BnFA^+^ to FA^+^ confirms that this close contact is true for almost all BnFA^+^ cations in this material.

However, this result alone does not prove that the BnFA^+^ is in atomic proximity to FA^+^ cations that are in the 3D FAPbI_3_ structure. For example, lower dimensionality phases, such as those from the 2D Ruddlesden–Popper class, (BnFA)_2_FA_*n*–1_Pb_*n*_I_3*n*+1_, could be possible, even at low BnFA^+^ concentrations. These materials contain FA^+^ cations whose ^1^H MAS NMR signals would be largely indistinguishable from those of FAPbI_3_. To address this ambiguity, we investigate the effect of BnFA^+^ incorporation on the 3D perovskite phase by employing ^127^I NQR. This technique directly and specifically interrogates the iodide sites in the α-FAPbI_3_ phase.^36^ Due to the large quadrupole moment of ^127^I (−71 Q fm^–2^), even very slight changes in structure substantially alter the electric field gradient at the ^127^I nucleus, altering its NQR transition frequency. We have previously shown that the ^127^I NQR transitions are extremely sensitive to distortion of the cubooctahedral symmetry of the FAPbI_3_ structure.^55^ Trace (<1 at %) substitution of iodide by chloride in α-FAPbI_3_ introduced enough disorder to generate a distribution of electric field gradient across inequivalent ^127^I nuclei, broadening the corresponding NQR transition.^36^ The use of BnAm as an additive in FAPbI_3_ thin films and in mechanosynthesis nearly doubles the full-width half-maximum of the NQR transition relative to pristine FAPbI_3_ (Figure 3g). This result indicates that BnFA^+^ is interacting directly with the 3D perovskite structure. Given the large size of this cation, it is unlikely that BnFA^+^ is incorporated into the bulk structure on the perovskite A-site. Instead, this result is consistent with two plausible binding modes: (1) BnFA^+^ binding via the amidinium group into vacant perovskite A-site at the surface of halide-terminated crystalline domains. (2) 2D perovskite phases, such as of the family (BnFA_2_FA_*n*–1_)Pb_*n*_I_3*n*+1_, with very large average *n* values (e.g., ⟨*n*⟩ > 100), such that ^127^I nuclei distant from the large BnFA^+^ cation experience an environment approximately the same as the bulk 3D FAPbI_3_ phase, and thus display similar NQR frequencies. Experiments interrogating the spin–spin relaxation time (*T*_2_) of BnFA^+^ in contact with 3D α-FAPbI_3_ (Figure S36) suggest that these cations are relatively dynamic within the structure. This is unlikely in the case of a 2D perovskite structure where strong π-interactions and steric interactions between adjacent BnFA^+^ cations are expected to significantly increase molecular rigidity. On this basis, we favor the first of these two proposed modes of incorporation, namely that BnFA^+^ binds via the amidinium group into vacant perovskite A-sites at the surface. As discussed in the Supporting Information (Figure S36), we also note the absence of ^1^H signals corresponding to BnFA^+^ in an isolated phase in the liquid-assisted grinding material further supports this model.

However, as noted above, domain-capping is not the only active mode of BnFA^+^ incorporation; at higher concentrations of the additive, both X-ray (Figures S27 and S30) and NMR (Figure S36) analysis suggests that in addition to BnFA^+^ capping perovskite domains, excess BnFA^+^ is segregated into an isolated low-dimensionality phase. Crucial to this unified model is the resolution with ^1^H MAS NMR of both modes of incorporation present in a single thin film material (FAPbI_3_ + 5 mol % BnAm, Figure S36 and succeeding text). We show this concentration-dependent model schematically in Figure 3h.

In summary, we use a combination of ^1^H and ^13^C MAS NMR to first demonstrate that BnFA^+^, not BnAm, is the active additive in our optimized material and that we can mimic BnFA^+^ formation and incorporation into the perovskite phase using a mechanosynthesis method. Next, we use a ^1^H–^1^H spin diffusion NMR experiment to show that BnFA^+^ is in atomic-level contact with FA^+^, followed by ^127^I NQR spectroscopy to confirm that this atomic-level contact is with FA^+^ specifically inside the 3D perovskite phase, rather than an isolated lower dimensionality phase. Taking these data together we conclude that BnFA^+^ formed following BnAm addition predominantly binds to A-site vacancies on the surfaces of perovskite domains and domain boundaries. Taking the average polycrystalline grain dimensions extracted via SEM imaging (Figure S25; 350 nm × 350 nm × 500 nm) to represent these domains, under this proposed model the optimized ∼0.25 mol % BnFA^+^ found via quantitative ^1^H MAS NMR occupy ∼16% of the surface A-sites.

### Benzylamine in Medium and Wide-Bandgap Perovskite Solar Cells and Device Stability

Finally, with this new understanding of the structural changes underpinning the BnAm treatment, we can now predict that an alternative, equivalent strategy for passivation should be possible: replacement of some FAI with a small amount of BnFAI in the perovskite precursor mixture, instead of the use of BnAm as an additive.

**Figure 4:**
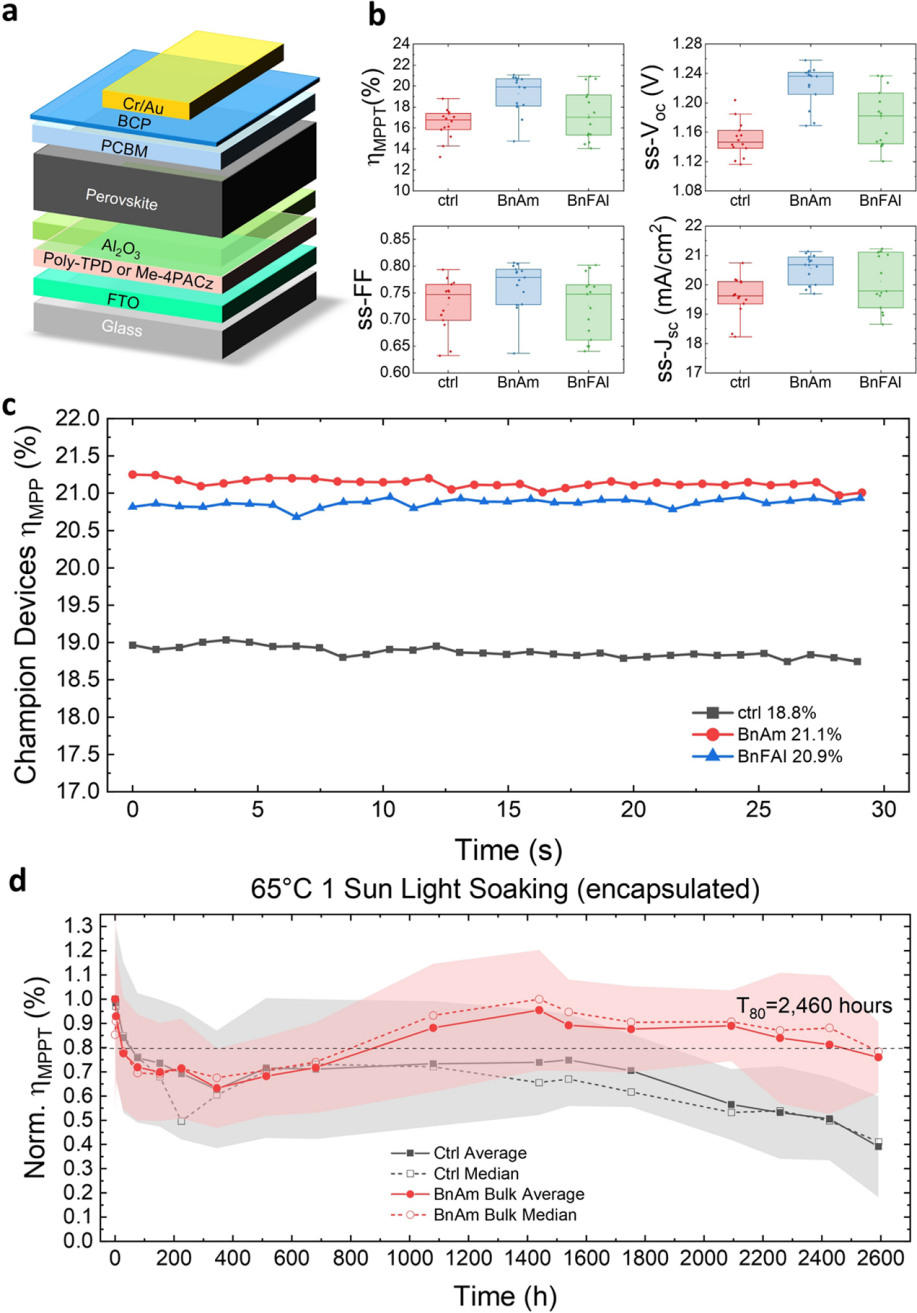
(a) Schematic of the p–i–n device structure used. (b) Statistical histogram of 1.68 eV perovskite p–i–n devices parameters without additive, perovskite with 0.3 mol % BnAm additive, and perovskite with 0.3 mol % BnFAI additive (15 devices each with Me-4PACz as hole-transporting layer (HTL) and antireflective coating). (c) 30 s of maximum power point tracking efficiencies (η_MPP_) of FA_0.75_Cs_0.25_Pb(I_0.8_Br_0.2_)_3_ champion devices for the three conditions: control, perovskite with 0.3 mol % BnAm, and perovskite with 0.3 mol % BnFAI replacing FA^+^. (d) 65 °C AM 1.5G aging of p–i–n devices with the 1.68 eV FA_0.75_Cs_0.25_PbI_0.8_Br_0.2_ (with Poly-TPD as HTL and encapsulated). The colored area denotes the standard deviation. The BnAm bulk condition is perovskite with 0.3 mol % additive. Devices were aged under open-circuit voltage conditions. Each data point is the average η_MPP_ of 6 devices for each condition. The data is normalized to show *T*_80_.

We now compare device performance when either of our passivation strategies is employed in a 1.68 eV PSC: (a) 0.3 mol % BnAm as an additive or (b) 0.3 mol % BnFAI replacing FAI. Among p–i–n PSCs with the 1.68 eV perovskite, both BnAm addition and BnFAI substitution show enhancement in device performance (Figure 4b), corroborating our conclusions that these treatments are similar. The BnAm additive devices have higher *V*_oc_ which could be due to the release of NH_3_ in the process of film formation that increased the grain sizes as discussed before. While we see some enhancement in steady-state *J*_sc_ (ss-*J*_sc_) with BnAm and BnFAI additives, the major improvement is in their open-circuit voltage, from 1.16 ± 0.04 to 1.22 ± 0.03 V for the BnAm additive devices and 1.18 ± 0.04 for the BnFAI additive devices. Overall, the average maximum power point tracked (η_MPPT_) efficiencies improved from 16.8 to 19.3% with BnAm additive and to 17.3% with BnFAI additive. The 30 s η_MPPT_ for the champion devices under each condition is shown in Figure 4c, while the champion control device η_MPPT_ is 18.8%, the champion BnAm and BnFAI additive devices increased to 21.1 and 20.9%, respectively.

Long-term photothermal stability of PSCs is critical for their commercial application, and is particularly challenging due to multiple active degradation pathways.^8,58−60^ We have already shown (Figure 1c,d) that BnAm addition is effective in reducing the rate of halide segregation in perovskite thin films. Therefore, we now investigate the long-term operational stability of solar cells. We select two perovskite compositions, FA_0.8_Cs_0.2_PbI_3_ (medium bandgap) and FA_0.75_Cs_0.25_Pb(I_0.8_Br_0.2_)_3_ (wide bandgap), suitable for single-junction and all-perovskite tandem PSCs, and integrate these into a device architecture reported to be stable: FTO/poly-TPD/Al_2_O_3_-NPs/perovskite/PCBM/BCP/Cr/Au as shown in Figure 4a.^61,62^

Initial stress testing of the medium-bandgap solar cells under maximum power point tracking (MPPT) for 1 h (Figure S46) shows rapid stabilization of devices with and without BnAm addition. Hence, we conduct long-term operational stability testing at *V*_oc_ on both the FA_0.8_Cs_0.2_PbI_3_ and FA_0.75_Cs_0.25_Pb(I_0.8_Br_0.2_)_3_ perovskites with the following treatments: (1) control devices with no BnAm additive, (2) devices with the optimum concentration of bulk additive (0.3 mol %). These devices were stressed under open-circuit voltage conditions in a Suntest CPS plus 65 °C (black standard temperature) aging box with ∼35% relative humidity (as measured under ambient conditions), simulated ∼0.7 sun illumination (xenon lamp), and AM 1.5 light spectrum.

First, we use unencapsulated medium bandgap PSCs to demonstrate accelerated aging under the effects of oxygen, moisture, heat, and light (Figure S48). While the control devices (*T*_80_ ∼ 35 h) and the devices with surface treatment (*T*_80_ ∼ 50 h) degraded rapidly, the BnAm additive devices were more stable and maintained a *T*_80_ of 108 h.

Next, we aged wide-bandgap PSCs encapsulated with UV-curable epoxy resin and glass coverslips under the same conditions (Figure 4d). After an initial “burn-in”, the BnAm additive devices gradually increase in efficiency again, achieving a remarkable *T*_80_ stability of 2460 h, while the control devices degraded to 30–50% of their initial maximum power point tracked efficiency values over the same time period. In Figure S49, we show stability plots of each device parameter as well as the stability of BnAm surface-passivated devices. These device aging results show that the in situ formed BnFA^+^ is a stable passivation for the perovskite and enhances the long-term operation stability of the solar cells.

## Conclusion

In this study, we investigate the complex chemical and optoelectronic consequences of adding BnAm to lead halide perovskite precursor solutions. BnAm reacts completely with FA^+^ in situ to produce BnFA^+^ and NH_3_ on the time scale of minutes, in a reaction that is general to many nucleophilic additives, including methylamine.^30−32^ At sufficiently high BnAm loading, secondary phase formation is observed in perovskite thin films fabricated from such solutions. By contrast, the BnAX (X = halide ions) additives do not react with the FA^+^ cation and do not produce identifiable new phases. By a combined solid-state NMR and NQR approach we demonstrate that BnFA^+^ incorporates into perovskite thin films at device-relevant concentrations and is in atomic-level contact with the 3D perovskite domains, but located at the domain boundaries and surfaces. These results are consistent with BnFA^+^ functioning as a “capping ion” covering the polycrystalline domains of the thin films. Notably, we find that at higher BnFA^+^ concentrations, isolated low-dimensionality phases form in addition to domain-capping. However, we note that this occurs at concentrations consistent with a marked drop in both optoelectronic (PLQY) and device performance. The incorporation of BnFA^+^ into the perovskite thin films improves photoluminescence and charge carrier lifetimes, indicating defect passivation. Additionally, the presence of BnFA^+^ slows down the rate of light-induced halide-segregation in mixed halide perovskite thin films. We show that this enhancement in optoelectronic properties of the perovskite films translates into an increase in PSC performance. Furthermore, BnFA^+^ helps protect the perovskite from combined light and heat-induced degradation, improving the device’s stability. Our BnAm bulk passivation produces PSCs with a median *T*_80_ of almost 2500 h at 65 °C under AM 1.5G illumination. The direct addition of BnFAI, which we synthesize and isolate, has an identical effect on device performance as BnAm addition, further corroborating our structural model. The approach of adding an aryl-containing amine demonstrates an under-explored but promising method of passivating perovskite defects while improving the perovskite stability. In particular, the structures of lead halide phases containing large-organic cations with FA^+^ head groups are poorly understood, with a considerable lack of atomic-level evidence that trace quantities of such organics are in the same phase as the 3D perovskite material. We anticipate that substantial further work will be conducted in this area.

## Data Availability

All relevant data are provided in the figures, tables, and Supporting Information.

## Supporting Information Available

Additional experimental details: materials and synthesis methods, X-ray diffraction, optical and electrical characterizations, PSCs characterizations, NMR spectroscopy, scanning electron microscopy, electron probe microanalysis (PDF)
