# Supplementary material for: Reactive Passivation of Wide-Bandgap Organic–Inorganic Perovskites with Benzylamine
Source: J Am Chem Soc. 2024 Sep 30;146(40):27405–16. doi: 10.1021/jacs.4c06659 (PMC11467896; doi:10.1021/jacs.4c06659)
Supplement: Supplementary file 1 — ja4c06659_si_001.pdf [file ja4c06659_si_001.pdf]

Supporting Information:

Reactive Passivation of Wide-Bandgap Organic-Inorganic Perovskites with Benzylamine

Suer Zhou [a], Benjamin M. Gallant [a,b], Junxiang Zhang [c], Yangwei Shi [d,e], Joel Smith [a], James N. Drysdale [a], Pattarawadee Therdkatanyuphong [c,g], Margherita Taddei [d], Declan P. McCarthy [c], Stephen Barlow [c], Rachel C. Kilbride [h], Akash Dasgupta [a], Ashley R. Marshall [a], Jian Wang [d], Dominik J. Kubicki [b], David S. Ginger [d], Seth R. Marder [c,f], Henry J. Snaith [a]*

[a] Department of Physics, Clarendon Laboratory, University of Oxford Parks Road, Oxford, OX1 3PU, UK

[b] School of Chemistry, Molecular Sciences Building, University of Birmingham, Birmingham, B15 2TT, UK

[c] Renewable and Sustainable Energy Institute, University of Colorado Boulder, Boulder, CO 80303, USA

[d] Department of Chemistry, University of Washington, Seattle, WA, 98195-1700, USA

[e] Molecular Engineering & Sciences Institute, University of Washington, Seattle, WA, 98195-1700, USA

[f] Department of Chemical and Biological Engineering, Department of Chemistry, and Materials Science and Engineering Program, University of Colorado Boulder, Boulder CO 80309, USA

[g] Department of Materials Science and Engineering, School of Molecular Science and Engineering, Vidyasirimedhi Institute of Science and Technology, Wangchan, Rayong 21210, Thailand

[h] Department of Chemistry, Brook Hill, The University of Sheffield, Dainton Building, Sheffield S3 7HF, UK

*Corresponding author: henry.snaith@physics.ox.ac.uk

Table of Contents

1. Experimental Methods ... 3
  1.1. Materials and general methods ... 3
  1.2. Thin Film Synethesis ... 4
  1.3. n-i-p Device Fabrication ... 4
  1.4. p-i-n Device Fabrication ... 6
  1.5. Perovskite Mechanosynthesis ... 8
2. Thin Film and Device Characterization ... 9
  2.1. UV-vis Absorption Measurement ... 9
  2.2. Ellipsometry ... 9
  2.3. Steady-State and Time-Resolved Photoluminescence ... 9
  2.4. Photoluminescence Quantum Yield (PLQY) ... 9
  2.5. PL Imaging ... 10
  2.6. AFM ... 10
  2.7. X-Ray Diffraction (XRD) ... 10
  2.8. Scanning Electron Microscopy (SEM) ... 10
  2.9. GIWAXS ... 11
  2.10. 2D XRD ... 11
  2.11. Solution NMR ... 11
  2.12. Solid-State NMR ... 11
  2.13. Current Density-Voltage (J-V) Measurements ... 12
  2.14. External Quantum Efficiency (EQE) Measurements ... 13
  2.15. 65 °C and 85 °C AM 1.5 Light-Soaking Box ... 13
  2.16. ToF-SIMS ... 13
3. Further Results and Discussion ... 14
  3.1. BnAm as a Bulk Additive in Perovskite Thin Films ... 14
    3.1.1. Steady-State PL Analysis ... 14
    3.1.2. Ellipsometry Measurements ... 18
    3.1.3. Time-Resolved PL Analysis ... 19
  3.2. BnAm Surface Treatment ... 23
  3.3. Solution Nuclear Magnetic Resonance (NMR) Spectroscopy ... 26
  3.4. Synthesis and Isolation of BnFAI and Bn_2FAI ... 30
    3.4.1. Synthesis of *N*-benzylformamidinium Iodide (BnFAI) ... 30
    3.4.2. Synthesis of *N N'*-dibenzylformamidinium Iodide (Bn_2FAI) ... 31
    3.4.3 Solution NMR of Pre-synthesized BnFAI and Bn_2FAI ... 32
  3.5. Perovskite Phase and Morphology ... 38

3.5.1. SEM Images of Thin Films 38
3.5.2. GIWAXS 41
3.5.3. BnAm Additive at High Concentrations........ 43
3.5.4. Comparison of Adding BnAm and BnFAI and BnAm Order of Addition 44
3.6. Solid-State Nuclear Magnetic Resonance Spectroscopy 49
3.7. Perovskite Solar Cells........ 55
3.7.1. n-i-p Perovskite Solar Cells 56
3.8. BnAm and BnFAI comparison in MA-free perovskite solar cells........ 58
3.9. Perovskite Solar Cell Stability 63
4. References........ 66

1. Experimental Methods

1.1. Materials and general methods

Tin(II) chloride dihydrate (99.995%), urea (≥98%), hydrochloric acid (37%), and thioglycolic acid (≥99%) were purchased from Sigma-Aldrich. Ultrapure water was purchased from Cambridge Bioscience. Formamidinium iodide (FAI) (99.999%) was purchased from Dyenamo. Lead(II) iodide (PbI_2) (99.999%) and cesium iodide (CsI) (>99.999%) were purchased from Alfa Aesar. Lead(II) bromide ($PbBr_2$) (>98.0%) and Me-4PACz (4-(3,6-Dimethyl-9H-carbazol-9-yl)butyl)phosphonic acid (>99.0%) was purchased from TCI. 2,2′,7,7′-tetrakis(N,N′-di-p-methoxyphenylamine)-9,9′-spirobifluorene (Spiro-OMeTAD) (>99.5%) was obtained from Lumtech. Poly[N,N' -bis(4-butylphenyl)-N,N' -bis(phenyl)-benzidine] (Poly-TPD) was purchased from 1-Material. PC61BM or PCBM (>99.5%) was purchased from Special Carbon Products. Bathocuproine ($C_{26}H_{20}N_2$ > 98%) was purchased from TCI Chemicals. Lithium bis-(trifluoromethanesulfonyl)imide (Li-TFSI, >99%), 4-tert-butylpyridine (tBP, 98%), isopropanol (IPA, 99.5%), N, N-dimethylformamide (DMF, anhydrous 99.8%), dimethyl sulfoxide (anhydrous DMSO, >99.9%), anisole (anhydrous, 99.7%), chlorobenzene (CB, 99.8%), acetonitrile (ACN, anhydrous 99.8%) were purchased from Sigma-Aldrich. Chemicals were used as received without further purification. Gold pellets and chromium bar for evaporation were purchased from Kurt J. Lesker.

BnFAI and Bn_2FAI were purified on Biotage® Isolera One, with C18 cartridge as the stationary phase and MeOH/water as the mobile phase.

1.2. Thin Film Synthesis

For the BnAm and BnFAI Surface Treatment

We apply benzylamine (BnAm) as a surface treatment for wide-bandgap perovskites as was done in reference.[1,2] We used perovskite composition $FA_{0.83}Cs_{0.17}Pb(I_{0.75}Br_{0.25})_3$ to study the surface passivation effect of BnAm. In a nitrogen-purged glovebox, the $FA_{0.83}Cs_{0.17}Pb(I_{0.75}Br_{0.25})_3$ perovskite precursor solution (1.2 M) is prepared by dissolving FAI, PbI_2, $PbBr_2$, and CsI in 1 ml DMF: DMSO = 4:1 volume ratio according to the stoichiometry, which was filtered before deposition. The $FA_{0.83}Cs_{0.17}Pb(I_{0.75}Br_{0.25})_3$ perovskite films were spin coated at 4,000 rpm for 60 s. At 35 s, 120 μL of anhydrous chlorobenzene was dispensed onto the perovskite film, which was annealed at 100 °C for 40 minutes afterwards.

BnAm was dissolved in chlorobenzene (CB) with a volume ratio of 1 %, 3%, and 5%, respectively. The BnAm solution was dynamically spincoated onto the perovskite top surface with 3,000 rpm for 30 s. After the surface treatment, the perovskite films were annealed at 100 ˚C for 10 min.

1.3. n-i-p Device Fabrication

Pre-etched FTO substrates (Pilkington Tec 7) were cleaned by subsequently sonicating them in Decon 90 solution (1% vol. in D.I. water), D.I. water, acetone, isopropanol for 10 min each. Then they were dried in a stream of nitrogen and UV-ozone cleaned for 15 min. 17.4 mg/ml tin (II) chloride dihydrate was dissolved in isopropanol and spin-coated onto the FTO substrates at 3,000 rpm for 25 s under ambient atmosphere. Subsequently, they were dried on a hotplate at 100 °C for 10 min. Then the hotplate temperature was increased to 180 °C and the substrates were annealed for 1 hour and then they were cooled to room temperature. A chemical bath solution containing 200 mL D.I. water, 2.5 g urea, 50 uL thioglycolic acid, 2.5 mL hydrochloric acid and 540 mg Tin (II) chloride dihydrate was stirred until fully dissolved. The substrates were then laid inside a Pyrex glass dish with FTO side face up. The

chemical bath solution was added into to the glass dish and closed with a glass lid. The container was left in a 90 °C oven for 3 h for the chemical bath deposition. Afterward, the substrates were taken out and subsequently sonicated in D.I. water and isopropanol for 5 min each and dried with a nitrogen gun. Then the substrates were annealed again at 180 °C for 1 h.

Before PCBM deposition, the substrates were UV-ozone cleaned again for 15 minutes. The substrates were transferred into a nitrogen-filled glovebox. A 5 mg/ml PCBM in chlorobenzene solution was spincoated on the substrates at 5,000 rpm for 30 s. Then the substrates were annealed at 100 °C for 10 minutes.

A 1.2 M $FA_{0.75}Cs_{0.25}Pb(I_{0.8}Br_{0.2})_3$ (1% excess PbI_2) precursor solution was made by dissolving 154.8 mg FAI, 77.9 mg CsI, 132.1 mg $PbBr_2$ and 391.1 mg PbI_2 for every 1 mL 4:1 ratio of DMF:DMSO. For perovskite precursor solutions made with benzylamine in the bulk, a modified preparation was used. The perovskite precursors were first dissolved overnight in a DMF:DMSO solvent deficient system (if 1 mL desired final volume, precursors dissolved in 950 μL 4:1 DMF:DMSO). Afterward, a 50 μL concentrated BnAm stock solution is added back into the perovskite precursor to achieve the intended (0~5 mol%) BnAm molarity. For example, for a 1 mol% BnAm additive perovskite precursor solution, 131 μL benzylamine is added to 5 mL of 4:1 DMF:DMSO (2.62% by volume), and then 50 μL of the stock solution is added to 950 μL solvent deficient perovskite precursor (5% x 2.62 vol%= 0.131 vol%). The molecular weight of benzylamine is 107.15 g/mol and the density at room temperature is 0.981 g/ml. As a result, 1 mol% of 1.2 M perovskite precursor is (1% x $1.2\text{x}10^{-3}$ mol/mL=0.000012 mol/mL). In volume percent, it is 0.000012 mol/mL x 107.15 g/mol ÷ 0.981 g/ml=0.131 vol%. Similarly, the 0.1 ~ 5 mol% BnAm additive perovskites were made by diluting the appropriate amount of BnAm into the DMF/DMSO solvents first and then added to the solvent-deficient perovskite precursor solutions. The precursor solutions were stirred for approximately 1 h and then filtered with a 0.22 μM PTFE filter before use.

The perovskite precursor solution was dropped dynamically onto the substrate spinning at 1,000 rpm for 10 s with 200 rpm/s acceleration and then 5,000 rpm for 45 s with 800 rpm/s acceleration. The antisolvent (1:1 diethyl ether: ethyl acetate) was

dropped onto the substrates 15 s before the end of the program. Then the substrates were annealed at 100 °C for 45 min.

For the hole-transporting layer, 85 mg/ml Spiro-OMeTAD is dissolved in chlorobenzene. 33 μL of tert-butyl pyridine (tBP) is added to 1 mL of the Spiro-OMeTAD solution first. A 517 mg/ml Li-TFSI stock solution in acetonitrile is made separately. Then 20 μL of the Li-TFSI solution is added per mL of Spiro-OMeTAD solution.

For the top metal contact, the evaporation chamber was pumped down until the pressure reaches <5 x 10^{-6} Torr. Then 80 nm of Au was evaporated at the rate of 0.1 Å/s for the first 10 Å and then 0.5 Å/s for the rest. For Cr/Au evaporation, first a 3.5 nm Cr was evaporated at the rate of 0.1 Å/s then the same Au program was subsequently used to evaporate 80 nm of Au.

1.4. p-i-n Device Fabrication

Pre-etched ITO substrates (Biotain Tec 10-15) were cleaned by subsequently sonicating in Decon 90 solution (1% vol. in D.I. water), D.I. water, acetone, isopropanol for 10 min each. Then they were dried in a stream of nitrogen and UV-ozone cleaned for 30 min. For device stability studies, pre-etched FTO substrates (Latech Tec-15) were used instead and cleaned using the exact same procedure as the ITO stated above.

For the hole-transporting layer, 1 mg/ml Poly-TPD was dissolved in toluene, stirred overnight at 80 °C, and filtered with a 0.22 μM PTFE filter before use. In an ambient environment, 200 μL of the 1 mg/ml Poly-TPD was dynamically spin-coated onto the substrates at 2,000 rpm for 20 sec with 2,000 rpm/s acceleration under ambient atmosphere. Subsequently, the substrates were dried on a hotplate at 130 °C for 10 min. The substrates were transferred into a nitrogen-filled glovebox. 500 μL of a 1:150 Al_2O_3 nanoparticles:isopropanol solution was statically spin-coated onto the substrates as a wetting layer at 5,000 rpm speed for 20 s with 5,000 rpm/sec acceleration and subsequently annealed at 100 °C for 2 min.

When Me-4PACz was used as the alternative hole-transporting layer, in a N_2 glovebox, Me-4PACz was dissolved in anhydrous ethanol at a concentration of 0.33 mg/mL. A

fluorine-doped tin oxide (15 Ω cm-2) substrate was first UV-ozone cleaned for a longer duration of 30 minutes. 300 μL Me-4PACz solution was statically dropped onto the substrate, and after 10 s it was spincoated at 3,000 rpm (600 rpm s^{-1}) for 30 s and then annealed at 100 °C for 10 min in the same environment.

A 1.4 M $FA_{0.8}Cs_{0.2}PbI_3$ (1% excess PbI_2) precursor solution was made by dissolving 192.6 mg FAI, 72.7 mg CsI, and 651.9 mg PbI_2 in 1 mL 3:1 ratio of DMF:DMSO. The perovskite precursor solution was stirred overnight and filtered with a 0.22 μM PTFE filter before use. For perovskite precursor solutions made with benzylamine in the bulk, a modified preparation was used. The perovskite precursors were first dissolved overnight in a DMF:DMSO solvent deficient system (if 1 mL desired final volume, precursors dissolved in 950 μL 3:1 DMF:DMSO overnight). 1 hour before spin-coating the perovskite layer, a 1 mol% stock solution of benzylamine was prepared by adding 153 μL benzylamine to 5 mL of 3:1 DMF:DMSO and diluted as necessary. The appropriate concentration of benzylamine bulk solution (0.2 mol% excess concentration found to be optimal for p-i-n devices) was then added to the solvent deficient perovskite precursor solution (if 1 mL desired final volume, added 50 μL benzylamine bulk solution to 950 μL perovskite precursor solution) and stirred for approximately 1 hour. The solution was then filtered with a 0.22 μM PTFE filter before use.

The perovskite precursor solution was dropped dynamically onto the substrate spinning at 1,000 rpm for 10 s with 200 rpm/s acceleration and then 5,000 rpm for 35 sec with 1000 rpm/s acceleration. 150 μL of anisole antisolvent was dropped onto the substrates 20 s before the end of the program. The substrates were then annealed at 100 °C for 45 min.

The 1.68 eV bandgap perovskite used in the p-i-n configuration follows the exact same fabrication procedure as the 1.2 M $FA_{0.75}Cs_{0.25}Pb(I_{0.8}Br_{0.2})_3$ used in the n-i-p configuration.

If benzylamine surface passivation was applied, a 25 mM solution of benzylamine was dissolved in isopropanol and stirred overnight. 100 μL of benzylamine surface passivation was dynamically spin-coated onto the cooled perovskite layer at 2,000 rpm

for 30 s with 2,000 rpm/sec acceleration. The substrates were then annealed at 100 °C for 1 min.

For the electron-transporting layer, 20 mg/mL PCBM was dissolved in chlorobenzene, stirred overnight, and filtered with a 0.22 μM PTFE filter before use. 40 μL of PCBM was dynamically spin-coated onto the substrates at 2,000 rpm for 30 s with 2,000 rpm/s acceleration and the substrates were subsequently annealed at 100 °C for 1 min. 0.5 mg/mL bathocuproine (BCP) was dissolved in isopropanol and stirred at 75 °C overnight before use. 100 μL of BCP was dynamically spin-coated on top of the cooled PCBM layer at 5,000 rpm for 20 s with 5,000 rpm/s acceleration and the substrates were then annealed at 100 °C for 1 min.

For the top metal contact, the evaporation chamber was pumped down until the pressure reaches <2 x 10^{-6} Torrs. Then 80 nm of Au was evaporated onto the substrates at the rate of 0.1Å/s for the first 50 Å, then 0.2 Å/s for the next 100 Å, then 0.5 Å/s for the next 100 Å, and finally 0.7 Å/s for the rest. A Cr/Au evaporation was used for device stability studies. For the Cr/Au evaporation, first 3.5 nm Cr was evaporated at the rate of 0.1 Å/s then the same Au program was subsequently used to evaporate 100 nm of Au.

1.5. Perovskite Mechanosynthesis

Reagents were stored inside a nitrogen glovebox at room temperature. Microcrystalline perovskite powders were synthesized by grinding the reagents in an electric ball mill (Retsch Mixer Mill MM-200) at 30 Hz for 30 minutes using a stainless-steel grinding jar (10mL) and a stainless-steel ball (⌀10 mm). The resulting powders were annealed at 150 °C for 15 minutes.

The following quantities of reagents were used. $FAPbI_3$: PbI_2 (299.7 mg, 0.65 mmol) and FAI (111.8 mg, 0.65 mmol). $FAPbI_3$ with 5 mol% BnFAI: PbI_2 (161.4 mg, 0.35 mmol), FAI (57.2 mg, 0.3325 mmol) and BnFAI (4.6 mg, 0.0175 mmol). $FAPbI_3$ with 5 mol% BnAm by liquid-assisted grinding (LAG): PbI_2 (276.6 mg, 0.6 mmol), FAI (103.2 mg, 0.6 mmol) and BnAm (3.21 mg, 3.28 uL, 0.03 mmol).

2. Thin Film and Device Characterization

2.1. UV-vis Absorption Measurement

Absorption spectra were measured in dilute solutions and on glass substrates using a UV/Vis-NIR spectrophotometer, Lambda 35 (Perkin-Elmer).

2.2. Ellipsometry

Optical constant values of refractive index (n) and extinction coefficient (k) were calculated using Woollam M-2000 ellipsometer with an integrated CCD camera, scan speed < 10 s, spot size > 1.5 mm and spectral range of 210-1700 nm. The fitting was done using B-spline function from default glass substrate with absorbing layer general method.

2.3. Steady-State and Time-Resolved Photoluminescence

Steady-state and time-resolved PL measurements were acquired using a time-correlated single-photon counting (TCSPC) setup (FluoTime 300, PicoQuant GmbH). Film samples were photoexcited using either a 532 nm/405 nm laser head (LDH-PC-532/LDH-P-C-405, Pico Quant GmbH) pulsed at a frequency of 5 kHz~1 MHz, with a pulse duration of 128 ps~4 ns and fluence of 221.1 nJ/cm^2 for the 532 nm laser and 242.1 nJ/cm^2 for the 405 nm laser. The PL was collected using a high-resolution monochromator and hybrid photomultiplier detector assembly (PMA Hybrid 40, PicoQuant GmbH) with a detection wavelength set to the peak PL emission wavelength from the sample.

2.4. Photoluminescence Quantum Yield (PLQY)

PLQY (or PLQE) values were determined following the method of De Mello *et al.*[3] using a 532 nm or 410 nm continuous wave laser excitation source (Roithner, RLTMLL-532 2 W) to illuminate a sample in an integrating sphere (Newport, 70682NS), and the laser scatter and PL were collected using a fiber-coupled spectrometer (Ocean Optics MayaPro). The beam intensity was modified using neutral density filters. To obtain PLQY values that correlate with a device operating under 1 sun illumination for a particular wavelength laser, an optical density filter was used to adjust the laser

output to the equivalent of AM 1.5G for each perovskite bandgap. See Figure 3 from Kirchartz *et al.* [4]

2.5. PL Imaging

The macroscopic photoluminescence (PL) mapping system used for measurements in this work is identical to the one employed in reference.[5] In short, the sample was illuminated using a 450 nm LED source (ThorLabs M450LP1). The PL was imaged using a cooled scientific CMOS sensor (ANDOR Zyla 4.2) coupled to a 50 mm lens set to a F5.6 aperture, and a long pass filter to block the excitation light. Images acquired using this setup in a raw format can be used to produce spatially resolved maps of the PL emission from the sample. Each pixel on the image was approximately 12 microns in real distance in this configuration.

The relative PL maps were converted into maps of estimated PLQY by taking an image of a white barium sulphate plate as a reference, and accounting for the relative wavelength of all components in the imaging setup (see reference[5] for details). This was then converted to a map of QFLS using the relationship:

$$QFLS^{i,j} = QFLS_{rad} + \frac{kT}{q}\ln(PLQY^{i,j}), \qquad (Equation\,S1)$$

Where i,j are indices of the pixels of the image, and $QFLS_{rad}$ is the radiative limited QFLS, approximated assuming perfect top-hat EQE with a sharp drop-off around the bandgap.

2.6. AFM

The morphologies of perovskite films were measured with Asylum Research MFP3D atomic force microscope mounted on an inverted optical microscope by using a 75 kHz tip.

2.7. X-Ray Diffraction (XRD)

XRD patterns were collected using a Panalytical X'Pert Pro X-ray diffractometer with a Cu Kα source (λ=1.54 Å).

2.8. Scanning Electron Microscopy (SEM)

Surface SEM images were collected using an FEI Quanta 600 scanning electron microscope at 5 kV acceleration voltage and a spot size of 3.0.

2.9. GIWAXS

GIWAXS was performed with a Xenocs Xeuss 2.0 system combining a Ga MetalJet source (Excillum) with a Pilatus3R 1M hybrid photon counting (HPC) detector (Dectris). X-rays with energy 9.243 keV (λ = 1.341 Å) were directed towards aligned samples at an incidence angle of 2.0°. Scattered X-rays were collected by the 2D HPC detector, positioned 290 mm from the sample. The sample chamber, detector and flight tube were held at vacuum during measurement to prevent air scattering. The alignment was calibrated using LaB_6, and the 2D data was integrated and reshaped using scripts based on the PyFAI and pygix libraries.[6]

2.10. 2D XRD

The Bruker D8 powder x-ray diffractometer (XRD) with extremely sensitive Pilatus 100 K large-area 2D detector (Cu Kα radiation) was used to measure the phase and crystallinity of benzylamine surface treated wide-bandgap perovskites, which provided 2D XRD images of the samples.

2.11. Solution NMR

The nuclear magnetic resonance (NMR) experiments (including ^{1}H, ^{13}C, COSY, and HSQC) were conducted using a two-channel Bruker Avance III HD Nanobay 400 MHz instrument running TOPSPIN 3 equipped with a 5 mm z-gradient broadband/fluorine observation probe or a Bruker Ascend 400 spectrometer. Deuterated dimethyl sulfoxide-*d*6 (DMSO- d_6) was used as the solvent for all the experiments.

2.12. Solid-State NMR

Thin film samples and mechanosynthesized materials were prepared as described above. Prior to measurement, thin film samples deposited on glass substrates were removed from the substrate by mechanical exfoliation with a razor blade to produce thin film powders. The powdered samples were packed into 1.3 mm (1 GHz, ^{1}H measurements) or 3.2 mm (500 MHz, ^{13}C and ^{127}I NQR measurements) zirconia rotors. Immediately prior to measurement, we heat all samples to ~150 °C with a heatgun (Bosch EasyHeat 500) to ensure materials are fully in the α-$FAPbI_3$ phase. We calibrated the temperature inside the rotor by attaching it to a thermocouple. This enabled us to identify the optimal heating conditions (flow rate, distance) to induce the phase transition. We are careful to heat the sample only gently with the heat gun in order to avoid thermally degrading the sample. The aim of the thermal treatment is only to ensure that the powdered thin film material is in the black cubic phase. Thus,

we apply the treatment only briefly (~30 seconds) and only for as long as until the material appears fully black to the eye. The phase transition from *2H*-FAPbI3 to alpha-$FAPbI_3$ occurs at ~85 °C. However, given the structural complexity of this phase transition, we have found that often to drive transformation a higher temperature is necessary, typically approximately 150 °C. We therefore quote this as the approximate maximum temperature achieved during heat gun treatment.

Solid-state MAS NMR spectra of ^{1}H (1000.4 MHz, 60 kHz MAS) were recorded on a Bruker Avance Neo 23.5 T spectrometer equipped with a 1.3 mm MAS probe using 100 kHz RF field amplitude. ^{1}H chemical shifts were referenced to adamantane (1.85 ppm). Quantitative ^{1}H spectra were recorded using a recycle delay of 300 s (The longest T_1 time of 40-50 s corresponded to the FA moieties). ^{1}H-^{1}H spin-diffusion spectra were recorded with a recycle delay of 0.2 s, mixing time of 0.1 s, and 400 slices in the indirect dimension. ^{13}C MAS spectra were recorded on a Bruker Avance III 11.7 T (125.7 MHz, 15 kHz MAS) spectrometer equipped with a 3.2 mm CPMAS and referenced to solid adamantane (38.48 ppm for -CH_2-). For Hahn echo experiments, 83.3 kHz RF field amplitude was used, with a recycle delay of 10 s. For CPMAS experiments, 100 kHz RF field amplitude was used with recycle delay selected to optimize signal acquisition, based on measured ^{1}H T_1. 100 kHz ^{1}H decoupling was used for all ^{13}C experiments. The rotors were spun using dry nitrogen. ^{127}I NQR spectra were recorded on a Bruker Avance III spectrometer equipped with either a 2.5 or 3.2 mm CPMAS probe using 167 kHz RF field amplitude, a recycle delay of 0.05 s with the probe placed outside the stray field of the magnet. For NQR measurements, the sample was static.

2.13. Current Density-Voltage (J-V) Measurements

The photovoltaic devices were characterized in ambient conditions with the room temperature around 20~25 °C and 25% relative humidity under 1 sun AM 1.5G simulated sunlight generated by a class AAA WaveLabs Sinus-220 solar simulator, using a Keithley 2400 source meter. The intensity of the solar simulator was set to produce 100 mW/cm^2 equivalent irradiance using a certified KG3-filtered Si reference photodiode (Fraunhofer ISE). The voltage was swept at a rate of 0.32 V/s first with a forward sweep followed by a reverse sweep. For the n-i-p devices, the voltage scan range was from -0.2 V to 1.2 V. For the p-i-n devices, the voltage scan range was from

-1.3 V to 0.2 V. On each substrate, there were three devices with an area 0.25 cm^2 and one larger device with an area of 1.00 cm^2. The areas were defined using black anodized aluminium shadow masks in direct contact with the glass side of the substrates within enclosed sample holders. Unless otherwise stated, the maximum power point tracked efficiency (for 30 sec) is used instead of power conversion efficiency. The steady-state FF is calculated by η_{MPP}/(steady-state V_{OC} × steady-state J_{SC}).

2.14. External Quantum Efficiency (EQE) Measurements

External quantum efficiency was acquired with a custom build Fourier transform photocurrent spectrometer utilizing a Bruker Vertex 80v Fourier Transform Interferometer. Devices were calibrated to a Newport-calibrated silicon reference solar cell with known external quantum efficiency and illuminated with an AM1.5 filtered solar simulator. Device were masked with the same metal aperture masks as in the J-V measurements, with active areas 0.25 cm^2 and 1 cm^2.

2.15. 65 °C and 85 °C AM 1.5 Light-Soaking Box

The 65 °C and 85 °C light aging tests were done on encapsulated devices in an ambient atmosphere, either 65 °C or 85 °C, full spectrum pulsed light ATLAS SUNTEST XLS+ aging box. The (1,700 W) xenon lamp is pulsed at 100 Hz and averaged off to 1 sun intensity. Unless otherwise stated, the devices were aged under open-circuit conditions.

2.16. ToF-SIMS

ToF-SIMS depth profiles were collected using IONTOF TOF.SIMS5 spectrometer with a 25 keV Bi^{3+} cluster ion source in the pulsed mode with argon gas cluster ion source for sputtering. The mass range of m/z is in the range of 0-800. The sputtering was performed over 500 micron x 500 micron area at 6.97 nA for a dose of 5.1 x 10^{13} ions/cm^2.

3. Further Results and Discussion

3.1. BnAm as a Bulk Additive in Perovskite Thin Films

3.1.1. Steady-State PL Analysis

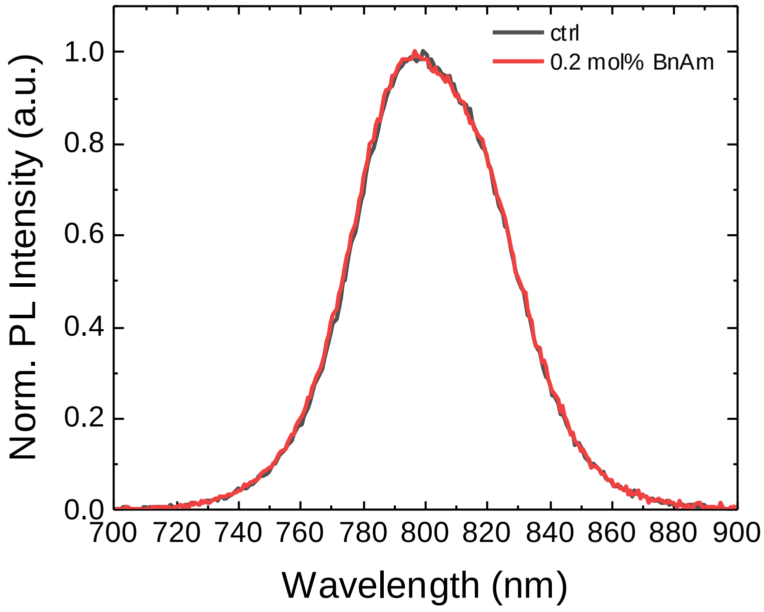

Supplementary Figure S1. Normalized photoluminescence quantum yield (PLQY) spectra of $FA_{0.8}Cs_{0.2}PbI_3$ perovskite with and without BnAm additive. Unlike in the case of $FA_{0.75}Cs_{0.25}Pb(I_{0.8}Br_{0.2})_3$ (**Figure 1a**), no peak shift is observed when BnAm is added to a pure iodide perovskite.

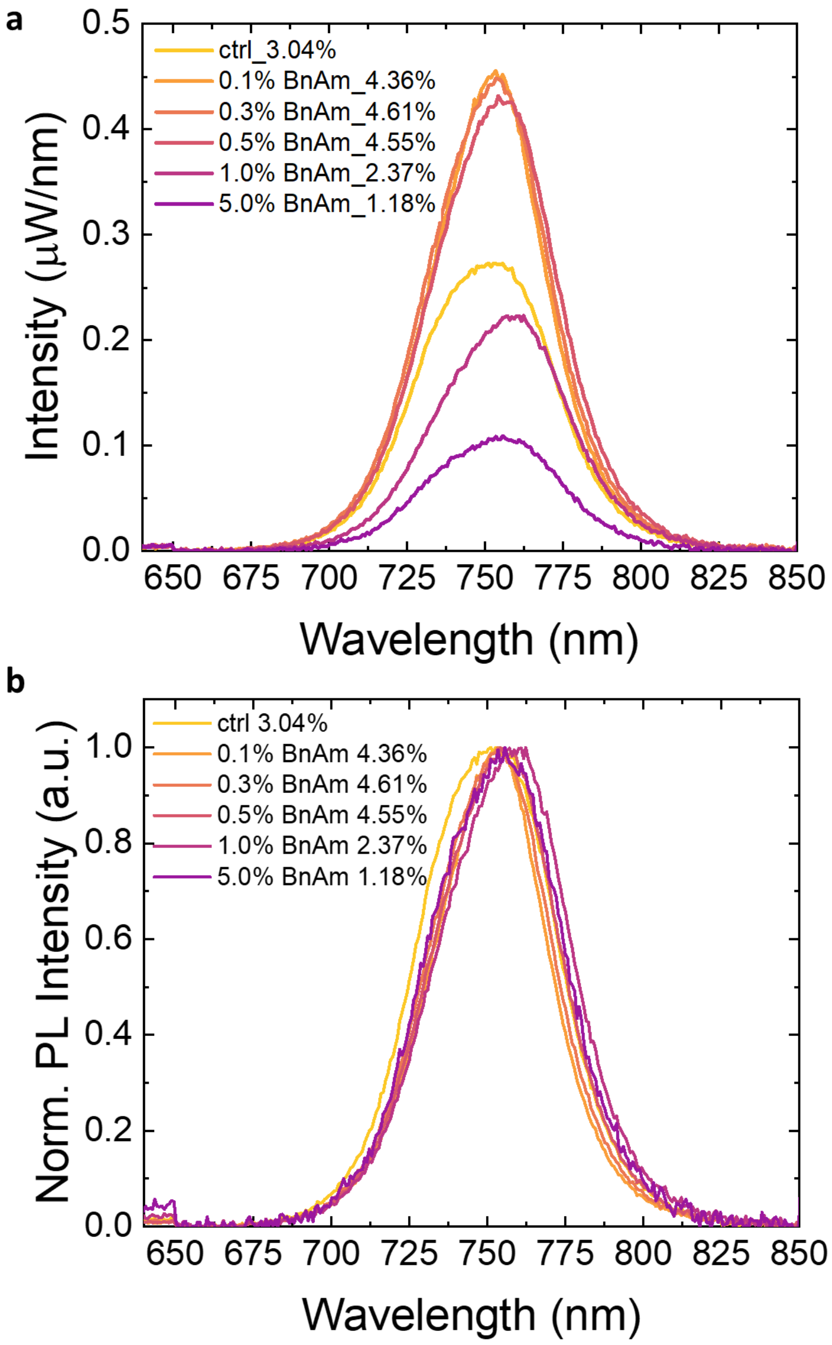

Supplementary Figure S2. (a) Photoluminescence quantum yield (PLQY) spectra of BnAm additive $FA_{0.75}Cs_{0.25}Pb(I_{0.8}Br_{0.2})_3$ perovskite with their best PLQY listed. (b) normalized plot of (a) to show peak position.

The Stokes Shift in energy is calculated by the following equation:

$$\boldsymbol{\Delta E = E_{abs} - E_{exc}} \qquad (Equation\ S2)$$

Where E_{abs} is the Tauc bandgap and E_{exc} is the PL emission peak energy.

Table S1. Stokes Shift calculation

BnAm conc. (mol%)	Wavelength (nm)	E_{exc} (eV)	E_{abs} (eV)	Δ E (eV)
0	752.7	1.64725	1.678	0.03075
0.1	753.4	1.64572	1.676	0.03028
1	757.2	1.63746	1.676	0.03854
5	755.7	1.64071	1.686	0.04529

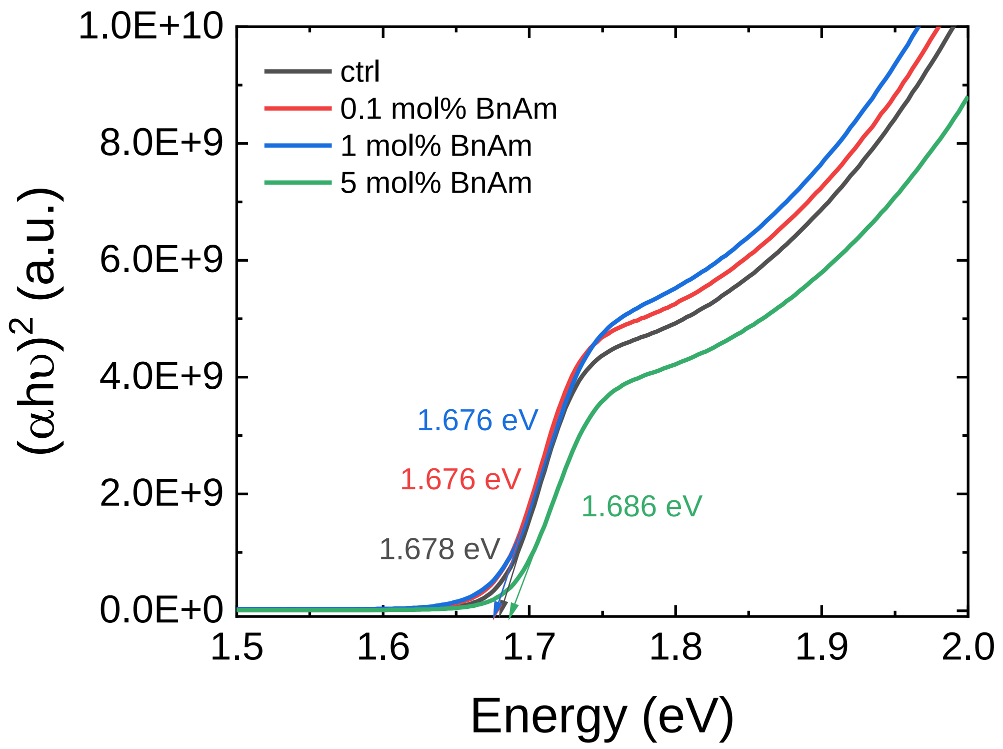

Supplementary Figure S3. Tauc plot of $FA_{0.75}Cs_{0.25}Pb(I_{0.8}Br_{0.2})_3$ perovskite with different amounts of BnAm additive.

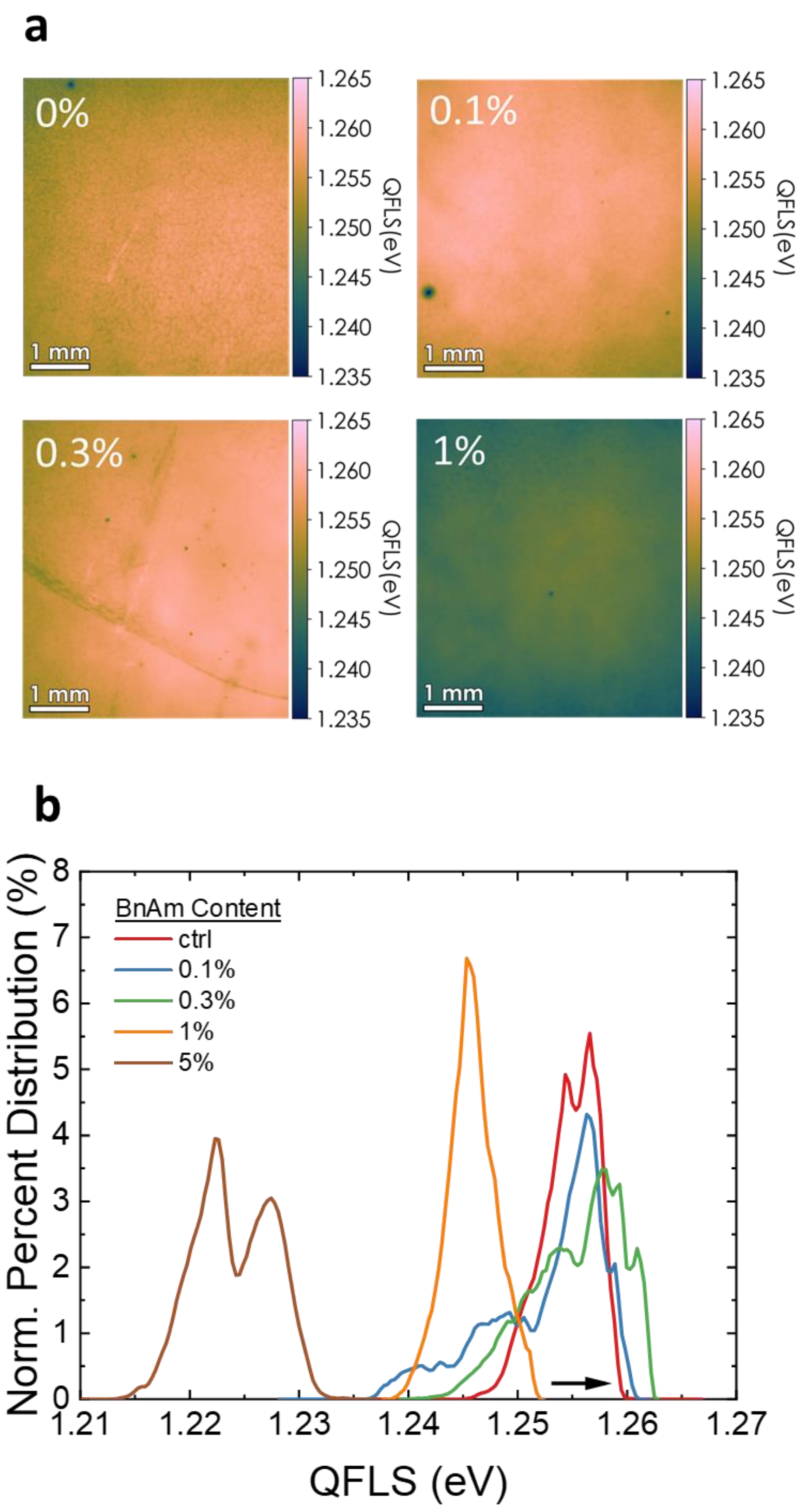

Supplementary Figure S4. (a) Spatially-resolved QFLS for perovskite films with different BnAm concentrations. (b) Quasi-Fermi level splitting (QFLS) of each sample calculated by measuring PLQY of four points from photoluminescence imaging**.**

3.1.2. Ellipsometry Measurements

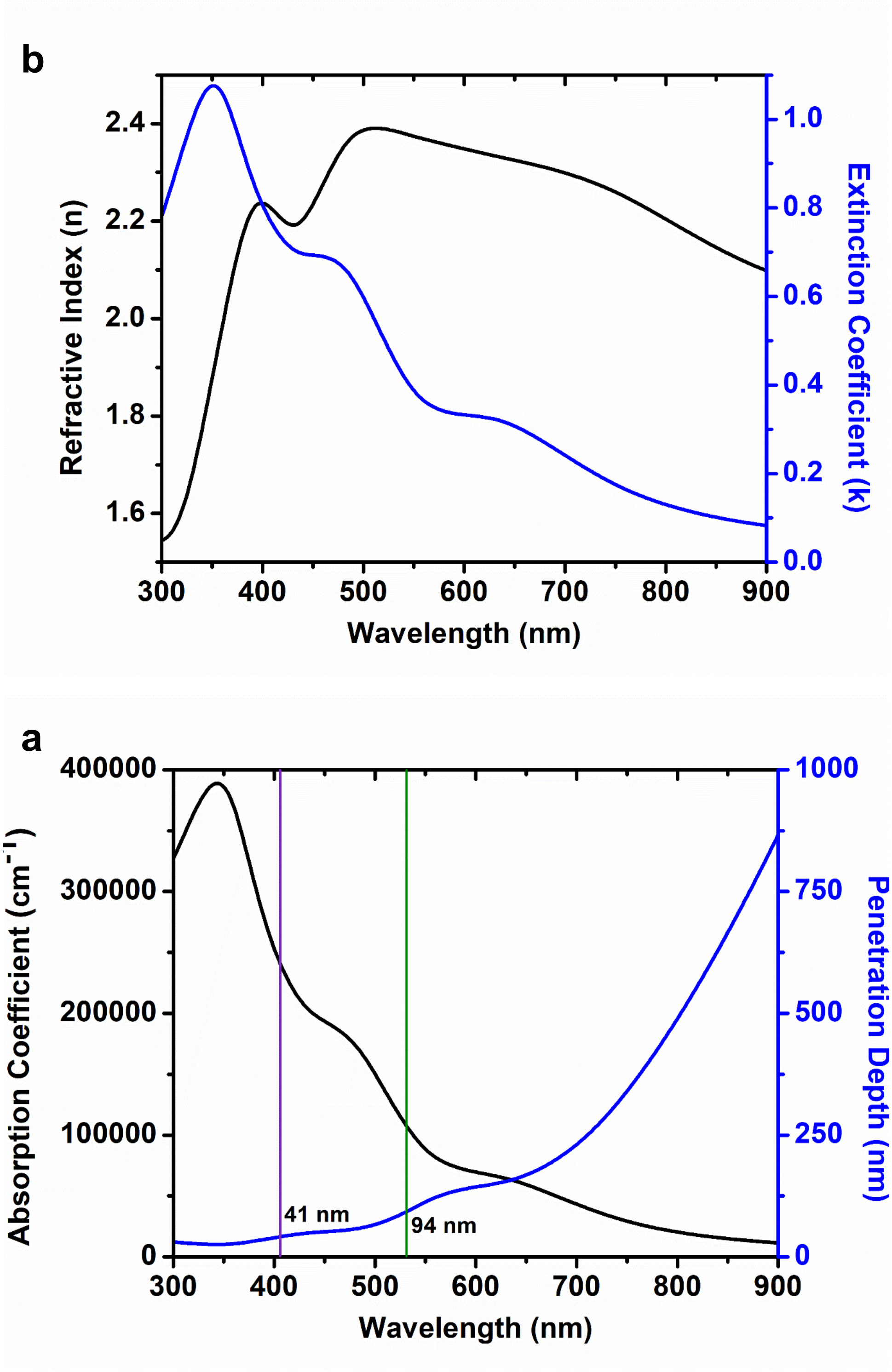

Supplementary Figure S5. Ellipsometry measurements of $FA_{0.75}Cs_{0.25}Pb(I_{0.8}Br_{0.2})_3$ perovskite thin film. (a) Refractive index (n) and extinction coefficient (k) from ellipsometry measurements of $FA_{0.75}Cs_{0.25}Pb(I_{0.8}Br_{0.2})_3$ perovskite thin film. (b) The absorption coefficient was calculated from the extinction coefficient following formula: $\alpha = \frac{4\pi k}{\lambda}$. The penetration depth is the inverse of the absorption

coefficient. The lines represent the values of α and penetration depth at the wavelength of excitation: 405 nm and 532 nm.

3.1.3. Time-Resolved PL Analysis

505 nm Laser Excitation

We use a stretched exponential function to fit the PL decays:

$$I = e^{-\left(\frac{t}{\tau_c}\right)^{\beta}} \qquad (Equation\ S3)$$

where β is the stretching exponent and τ_c is the characteristic lifetime. The physical meaning of β can be associated with the distribution of recombination lifetimes.[7,8] Smaller values of β generally indicate more distributed kinetics (more inhomogeneity) while values of β closer to unity indicate narrower distributions of lifetimes. In the case of β = 1, the decay function reduces to a single exponential, and the average lifetime is equal to τ_c. When 0< β <1, the average lifetime is calculated using **Equation S4**:

$$< \tau > = \frac{\tau_c}{\beta}\, \Gamma\left(\frac{1}{\beta}\right) \qquad (Equation\ S4)$$

First, we excite the perovskite films with a 532 nm laser for which the penetration depth is 94 nm of the 492 nm thick films, as measured by ellipsometry (see **Figure S5** for details). We fitted the time-resolved photoluminescence (TRPL) with a stretched exponential decay curve, according to **Equation S3** and **S4**. According to the TRPL fitting (**Figure S6**), the stretched exponential lifetime (τ) increases as more BnAm is added, up to 0.5 mol%. The τ increases from 185 ns for the control to 237 ns and 298 ns with the addition of 0.3 and 0.5 mol% of BnAm, respectively. The β value is highest, 0.65, for the 0.3 mol% film and decreases to 0.49 for a higher 0.5 mol% addition.

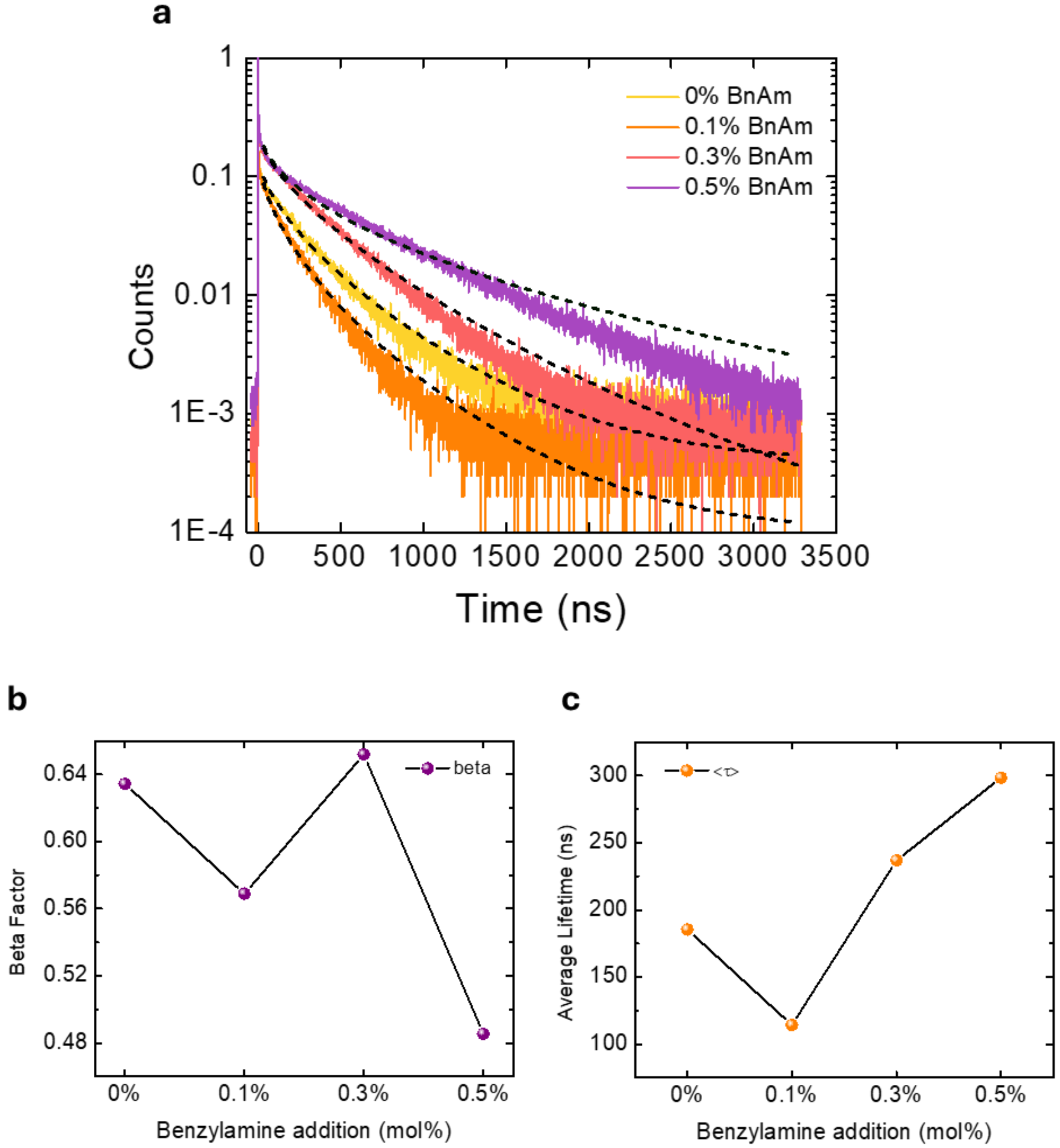

Supplementary Figure S6. (a) Time-resolved photoluminescence of BnAm with 0.1 mol%~0.5 mol% additive in $FA_{0.75}Cs_{0.25}Pb(I_{0.8}Br_{0.2})_3$ perovskite. Measured with a 532 nm laser, 300 kHz pulse frequency, and 10% excitation attenuation, with a fluence of 199.0 J cm^{-2}.

Table S2. Fitted charge carrier lifetime and β with varying BnAm concentration.

BnAm Concentration	τ (ns)	β
0 mol%	185.5	0.63
0.1 mol%	114.2	0.57

0.3 mol%	236.9	0.65
0.5 mol%	298.4	0.49

The beta value is 0<$\boldsymbol{\beta}$<1 and it indicates the heterogeneity of carrier lifetimes distribution. When beta is closer to 0, there is a more heterogenous distribution, whereas, when it is close to 1, the material has a more homogenous distribution.

405 nm Laser Excitation

Perovskite thin films with or without BnAm modification were also measured under a 405 nm laser excitation. According to the TRPL fitting results, the average lifetime, τ, between the front and back illumination was calculated. The average lifetime increased from 0~0.1 mol% BnAm and starts dropping again at ~0.3 mol%. While the control sample has an average lifetime of 243.5 ns, the 0.1 mol% and 0.3 mol% BnAm samples have enhanced lifetimes of 286.2 ns and 258.5 ns. However, we note that the lifetimes are not significantly different due to the long timescale of measurement. The β, however, consistently decreases as more BnAm is added, from β=0.63 at 0 mol% to β=0.32 at 5.0 mol%, which suggests that the compositional homogeneity is

dropping and there could be mixed BnFA-rich phases at the surface and grain boundaries.

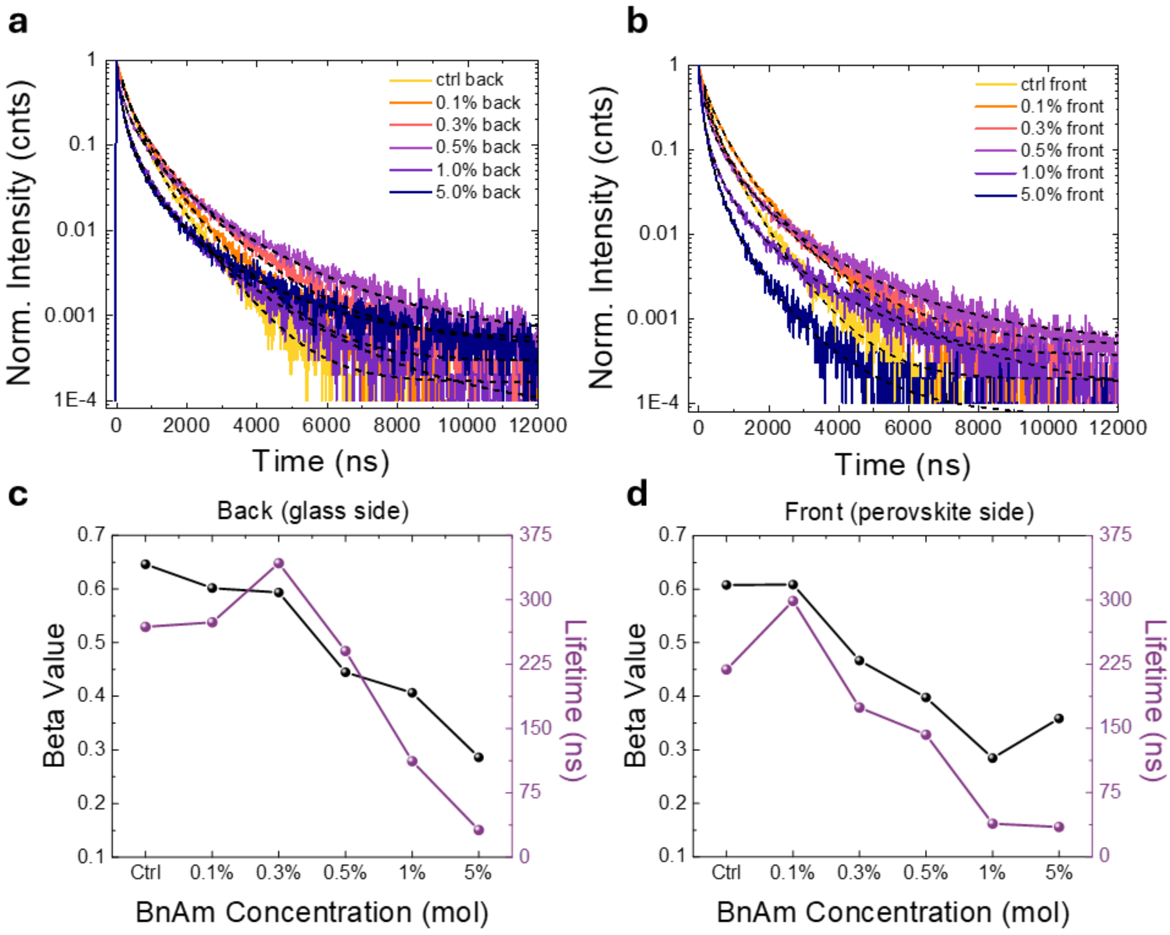

Supplementary Figure S7. Time-resolved photoluminescence of BnAm with 0.1 mol%~0.5 mol% additive in $FA_{0.75}Cs_{0.25}Pb(I_{0.8}Br_{0.2})_3$ perovskite (a) Illuminated from the glass side, (b) Illuminated from the perovskite side. Changes in beta value (left) and lifetime (right) with BnAm concentration variation in the film (c) from the glass side, (d) from the perovskite side. Measured with a 405 nm laser, 5 kHz pulse frequency, 10% excitation attenuation with a fluence of 217.9 J cm^{-2}. Data fitted with a stretched exponential decay fitting.

Table S3. Fitted charge carrier lifetime and β with varying BnAm concentration (both front and back).

	Front		Back		Average	
**BnAm Concentration**	**τ (ns)**	**β**	**τ (ns)**	**β**	**τ (ns)**	**β**
0 mol%	218.7	0.61	268.4	0.65	243.5	0.63
0.1 mol%	298.7	0.61	273.7	0.60	286.2	0.61
0.3 mol%	174.3	0.47	342.6	0.59	258.5	0.53
0.5 mol%	142.6	0.40	240.2	0.44	191.4	0.42
1.0 mol%	38.9	0.28	111.9	0.41	75.4	0.35

5.0 mol%	35.0	0.36	31.7	0.29	33.3	0.32

3.2. BnAm Surface Treatment

To gain an insight of the BnAm surface modification on perovskite films, X-ray diffraction (XRD) was conducted. As shown in **Figure S8**, 1 vol% (92 mM) BnAm surface treatment had negligible influence on the properties of the perovskite films. However, with 3 vol% (275 mM) BnAm modification, the surface formed low dimensional phases on top of the three-dimensional (3D) perovskite film. When the volume ratio of BnAm was further increased to 5 vol% (458 mM), the surface is fully converted to a 2D phase.

To understand the influence of BnAm surface treatment on the morphology of perovskite films, atomic force microscopy (AFM) was conducted. As shown in **Figure S9**, 1% BnAm treatment had negligible influence on the morphology of perovskite films. However, with 3 vol% BnAm treatment, there were two new phases perovskite formed on the 3D counterpart (**Figure S8**), one of which at 2θ~6˚ is likely the 2D phase, $BnFA_2PbI_4$ (with a d-spacing close to BnA_2PbI_4), and the other one at 2θ ~7˚ is an unidentified lower-dimensional phase. As the BnAm surface treatment concentration increases to 5 vol%, the proportions of these two new phases change, as less of the 7˚ phase is formed and more of the pure 2D phase is formed. It is worth mentioning that with 5 vol% BnAm treatment, it forms a uniform 2D capping layer on top of the 3D perovskite.

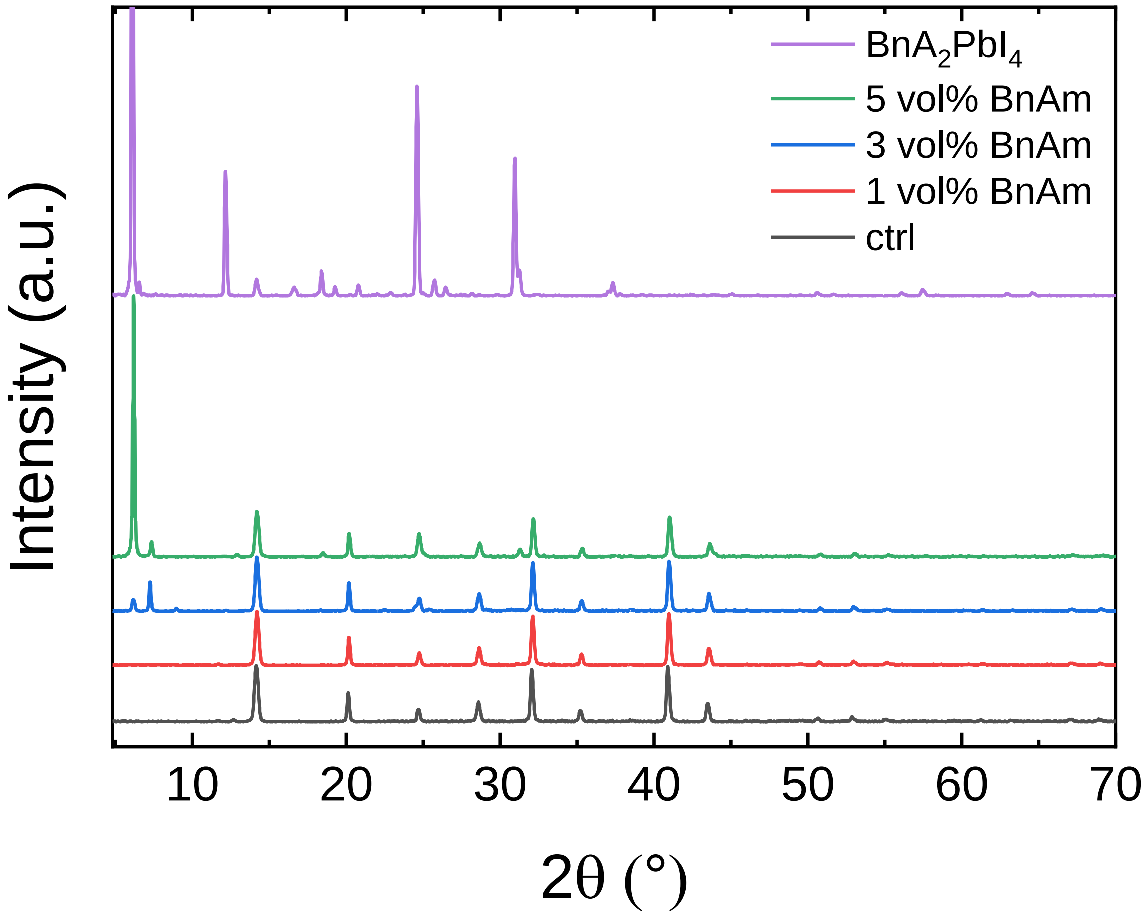

Supplementary Figure S8 XRD patterns of $FA_{0.83}Cs_{0.17}Pb(I_{0.75}Br_{0.25})_3$ perovskite films with 1 vol%, 3 vol%, and 5 vol% BnAm surface treatment. 1 vol% is about 91.6 mM. BnA_2PbI_4 solution was prepared by mixing BnAI and PbI_2 in DMF solution and spincoating a thin film on glass.

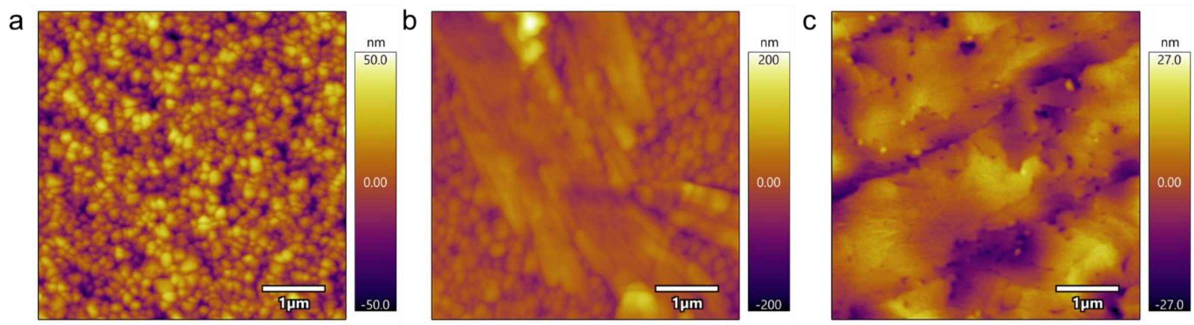

Supplementary Figure S9. Atomic force microscopy (AFM) measurement of (a) 1 vol%, (b) 3 vol%, and (c) 5 vol% BnAm surface treated perovskite films.

UV-vis absorption spectroscopy was further carried out to understand the influence of the BnAm surface treatment. **Supplementary Figure S10** shows that 1% volume

treatment had negligible influence on the absorption of the films. With 3% volume treatment, there is a substantial increase in below bandgap reflectance, which is consistent with rougher film surface. When the BnAm surface treatment is increased to 5% volume, the absorbance is significantly reduced. This is consistent with the XRD results because the formation of 2D and quasi-2D perovskite consumes the surface 3D perovskite which reduces the thickness of 3D films and thus results in the less absorption.

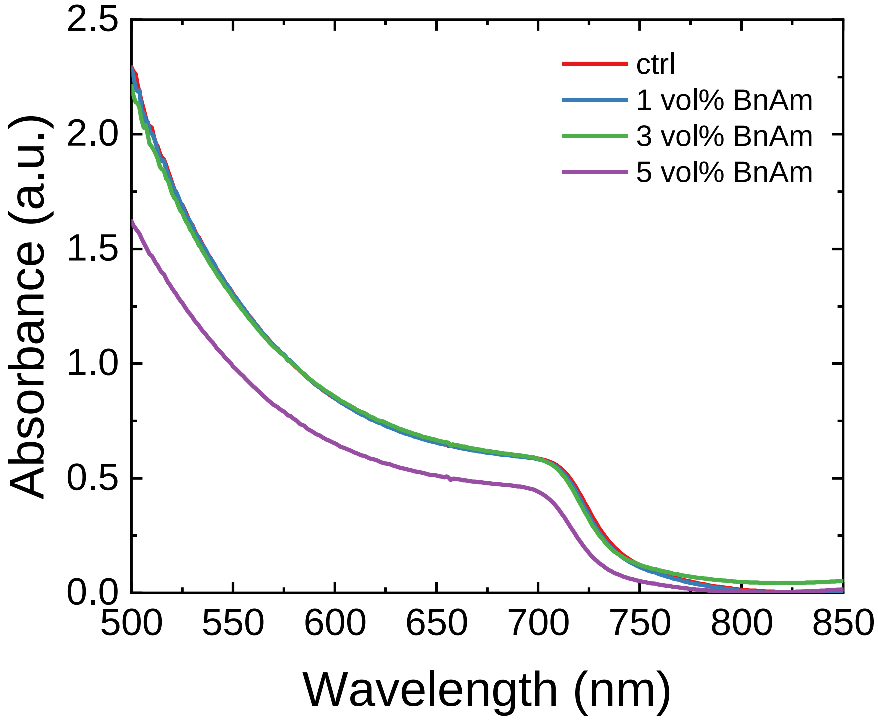

Supplementary Figure S10. UV-vis absorbance spectra of 1 vol%, 3 vol%, and 5 vol% BnAm surface treated $FA_{0.83}Cs_{0.17}Pb\ (I_{0.75}Br_{0.25})_3$ perovskite films.

3.3. Solution Nuclear Magnetic Resonance (NMR) Spectroscopy

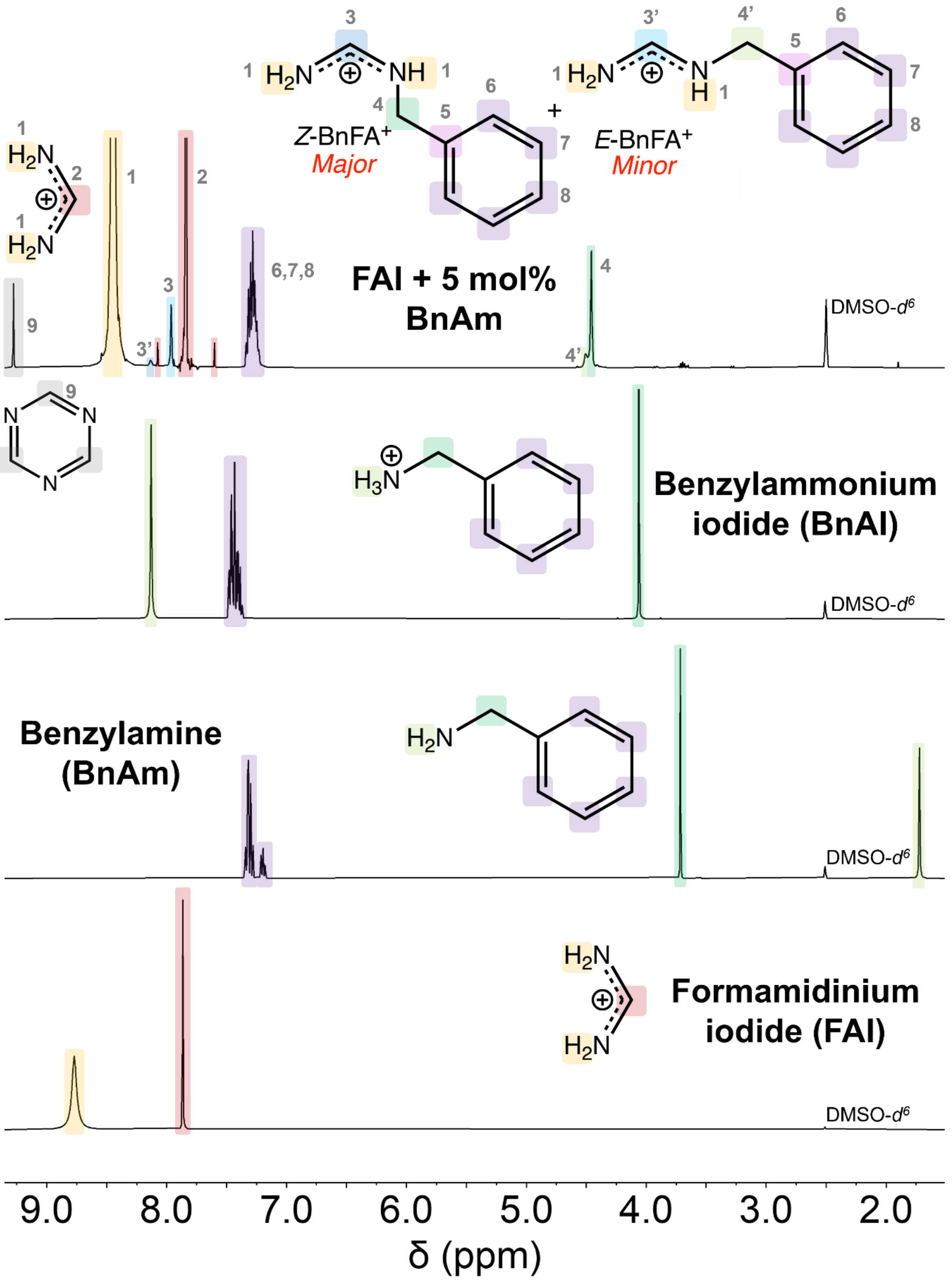

Supplementary Figure S11. ^{1}H nuclear magnetic resonance (NMR) spectra of (bottom to top) formamidinium iodide (FAI), benzylamine (BnAm), benzylammonium iodide (BnAI) and a mixture of

5:75 BnAm:FA+ all dissolved in DMSO-d_6 at equivalent concentrations. Although the presence of coordinating halides in solution can shift signals in solution NMR, if concentrations of all species are kept equivalent, we expect these effects to be consistent, particularly for ^{1}H shifts of C-H moieties. As such, that signals in the FAI + 5 mol% BnAm sample do not align with those in either BnAm or BnAI demonstrates that these species are not present in this solution.

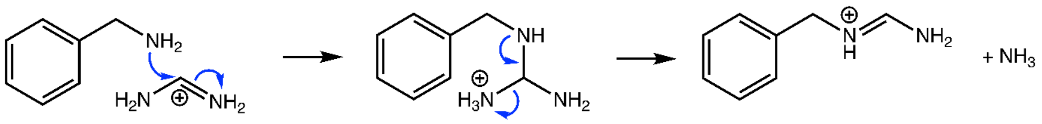

Supplementary Figure S12. Mechanism for formation of *N*-benzyl formamidinium ($BnFA^+$).

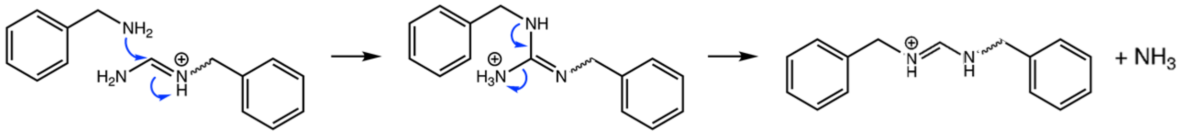

Supplementary Figure S13. Mechanism for formation of *N*,*N'*-dibenzyl formamidinium (Bn_2FA^+).

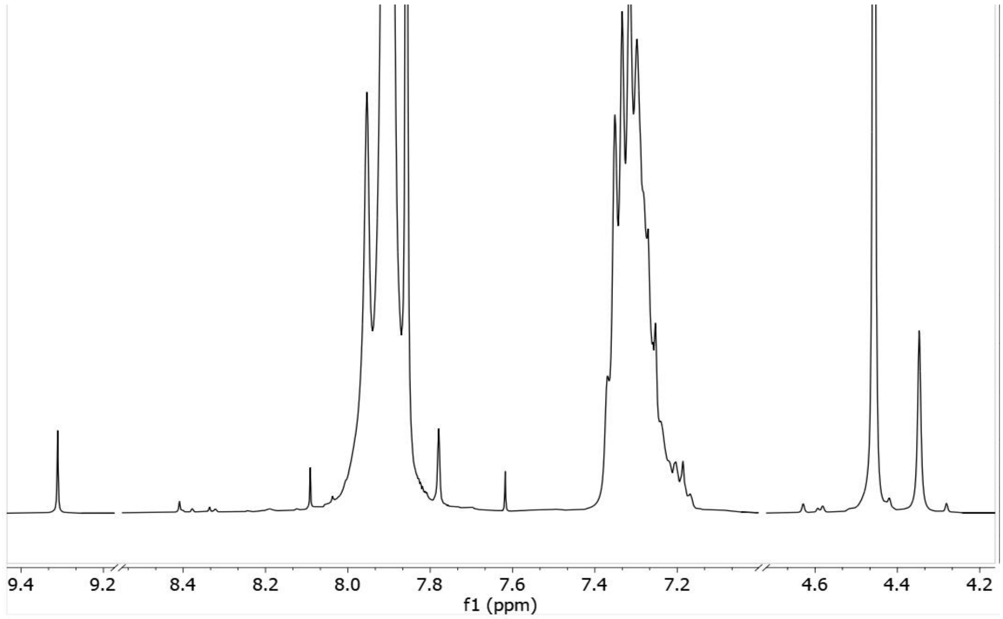

Supplementary Figure S14. ^{1}H NMR spectrum of a solution of FAI + 20 mol% BnAm in DMSO-d_6. Formation of $BnFA^+$ is apparent. However, an additional set of signals is observed, which comparison of the data in **Table 4** and the ^{1}H NMR spectrum of purified Bn_2FAI in **Supplementary Figure S23** suggests may correspond to the formation of Bn_2FA^+.

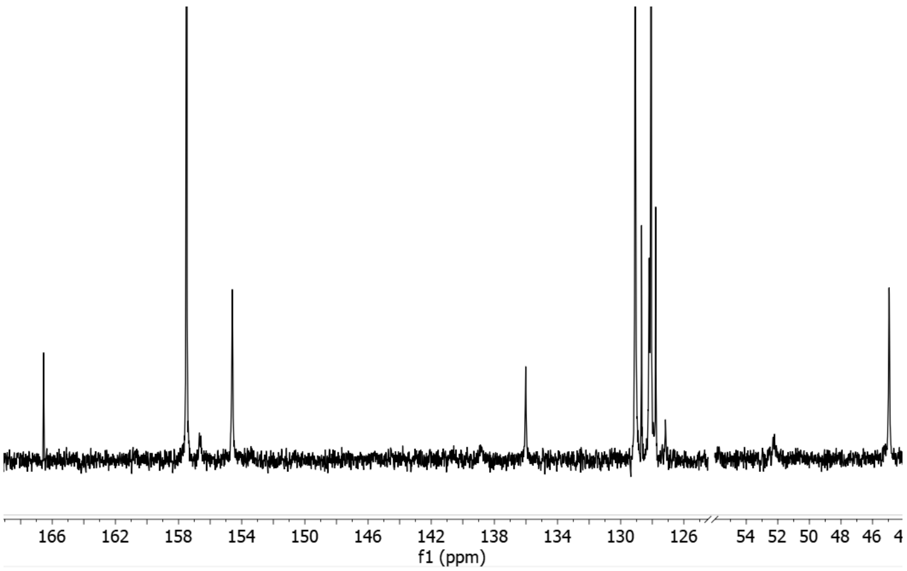

Supplementary Figure S15. $^{13}C\{^1H\}$ NMR spectrum of a solution of FAI + 20 mol% BnAm in *d6*-DMSO. Formation of $BnFA^+$ is apparent. However, an additional set of signals is observed, which comparison of the data in **Table 4** and the ^{13}C NMR spectrum of purified Bn_2FAI in **Supplementary Figure S21** suggests may correspond to the formation of Bn_2FA^+.

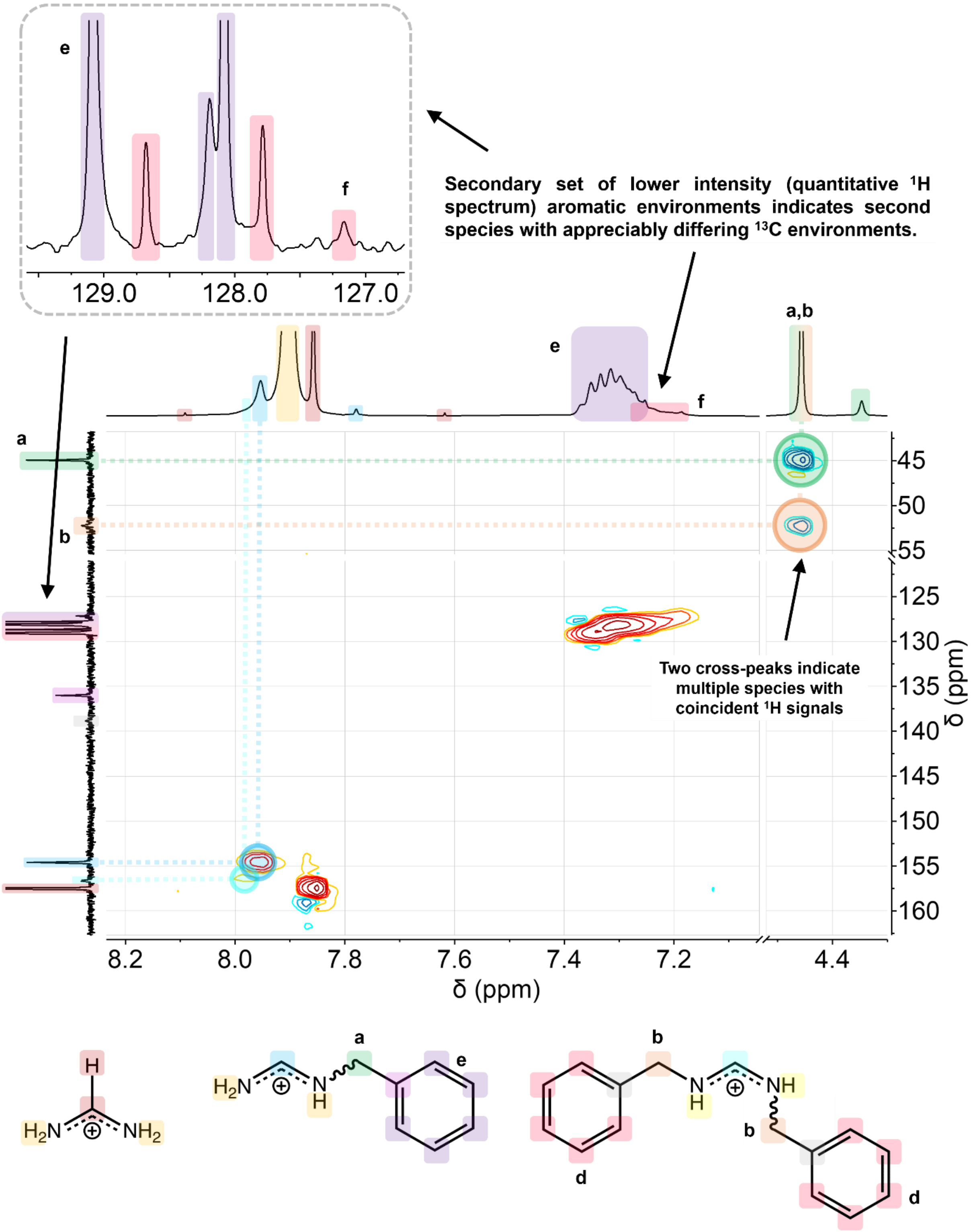

Supplementary Figure S16. ^{1}H-^{13}C heteronuclear single quantum coherence (HSQC) NMR spectroscopy of a solution of FAI + 20 mol% BnAm in DMSO-d_6. HSQC shows cross-peaks between ^{1}H and ^{13}C nuclei that are directly bonded. Correlation between signals labelled a and b indicate that a second, identical methylene signal, not found in $BnFA^{+}$ but with identical ^{1}H chemical shift, is present. As we also clearly observe the presence of a new set of peaks in both the ^{1}H and ^{13}C spectra

corresponding to a new aromatic region, we tentatively assign the additional signals observed as indicating the formation of small quantities of Bn_2FA^+.

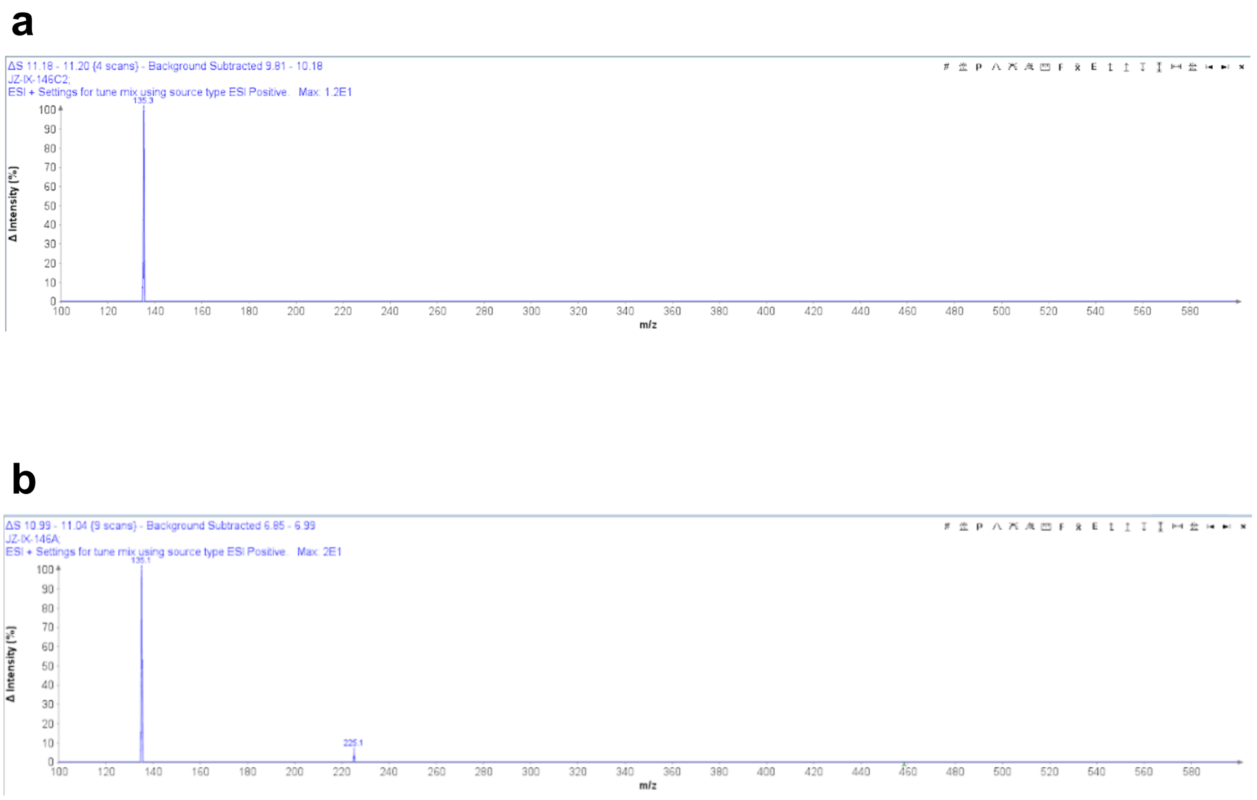

Supplementary Figure S17. Mass spectra of (a) the solution of FAI (1.4 M in 3:1 DMF:DMSO) and BnAm (0.2 mol%), immediately after mixing; (b) the solution of $FA_{0.8}Cs_{0.2}PbI_3$ (1.4 M in 3:1 DMF:DMSO) and BnAm (0.2 mol%), immediately after mixing. Ion peaks at *m/z* = 135.1 and 225.1 are assigned to mono- and bis- substituted products, $BnFA^+$ and Bn_2FA^+, respectively. Figure **a** indicates that immediately after mixing, the reaction is done and no BnA^+ (*m/z* = 108.1) can be detected by mass spectroscopy. Figure **b** suggests that addition of BnAm to pre-mixed perovskite solution generates Bn_2FA^+ besides $BnFA^+$, presumably because most FA^+ cations are already associated with PbI_2 in solution perovskite precursors so that limited free FA^+ is available for reaction with BnAm.

3.4. Synthesis and Isolation of BnFAI and Bn_2FAI

3.4.1. Synthesis of *N*-benzylformamidinium Iodide (BnFAI)

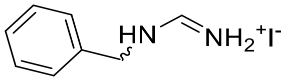

To a solution of formamidinium iodide (0.86 g, 5.0 mmol) in anhydrous methanol (50 mL) under ice bath, benzylamine (0.59 g, 5.5 mmol) in anhydrous methanol (100 mL) was slowly added over 2 h under argon. After addition, the resulting clear solution was concentrated to afford a colorless wax. Purification using reverse phase

chromatography yielded the product as a white solid (1.08 g, 82%, inseparable mixture of *Z* and *E* isomers in ca. 4:1 ratio, assigned based on J_{HH} values obtained in presence of CF_3CO_2H, which suppresses rapid exchange of NH and NH_2 protons with water). ^{1}H and ^{13}C{^{1}H} NMR spectra are shown in **Supplementary Figures S18-20**. Once formed, isomers do not appreciably interconvert.

^{1}H NMR (400 MHz, DMSO-d_6). Major (*Z*) isomer: *δ* 9.31 (br s, 3H, N*H*/NH_2), 8.03 (s,1H, NC*H*N), 7.51–7.22 (m, 5H, Ph C*H*s), 4.50 (s, 2H, CH_2). Minor (*E*) isomer: *δ* 9.31 (s, 3H, N*H*/NH_2), 8.15 (s,1H, NC*H*N), 7.51–7.22 (m, 5H, Ph C*H*s), 4.54 (s, 2H, CH_2). ^{1}H NMR (400 MHz, 25:1 v/v DMSO-d_6/CF_3CO_2H). Major (*Z*) isomer: δ 9.98 (app. d, J_{app} = 7 Hz, 1H, N*H*, *Z* side), 9.27 (app. d, J_{app} = 7 Hz, 1H, N*H*, *Z* side), 9.07 (d, *J* = 16 Hz, 1H, N*H*, *E* side), 8.02 (dt, *J* = 16, 7 Hz, 1H, NC*H*N), 7.48 – 7.25 (m, 5H, Ph C*H*s), 4.50 (d, J = 6 Hz, 2H, CH_2). Minor (*E*) isomer: 9.74 (app. d, J_{app} = 7 Hz, 1H, N*H*, *Z* side), 9.07 (app. d, *J* = 14 Hz, 1H, N*H*, *E* side), 8.73 (app. d, J_{app} = 14 Hz, 1H, N*H*, *E* side), 8.17 (td, *J* = 14, 7 Hz, 1H, NC*H*N), 7.48 – 7.25 (m, 5H, Ph C*H*s), 4.55 (d, J = 6 Hz, 2H, CH_2). ^{13}C{^{1}H} NMR (101 MHz, DMSO-d_6) Isomer mixture: *δ* 158.07, 154.98, 135.56, 129.19, 128.39, 128.13 (some aryl signals from the two isomers likely overlap), 49.96, 44.71. HRMS (ESI) calcd for $C_8H_{11}N_2^+$ (M^+-I), 135.0917; found, 135.0930. Anal. Calcd. for $C_8H_{11}IN_2$: C, 36.66; H, 4.23; N, 10.69. Found: C, 36.54; H, 4.22; N, 10.60.

3.4.2. Synthesis of *N N'*-dibenzylformamidinium Iodide (Bn_2FAI)

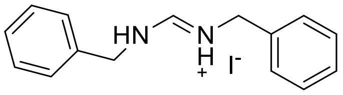

To a solution of formamidinium iodide (0.86 g, 5.0 mmol) in anhydrous methanol (50 mL) at room temperature, benzylamine (1.34 g, 12.5 mmol) in anhydrous methanol (50 mL) was added over 5 min under argon. After addition, the resulting clear solution was concentrated to afford a colorless wax. Purification by reverse phase chromatography yielded the product as a white solid consisting overwhelmingly of the *E,Z* isomer (1.50 g, 85%). ^{1}H and ^{13}C{^{1}H} NMR spectra are shown in **Figures S21-23**.

^{1}H NMR (400 MHz, DMSO-d_6): δ 9.93 (s, 2H, N*H*), 8.31 (s, 1H, NC*H*N), 7.60–7.27 (m, 10H, Ph C*H*s), 4.64 (s, 2H, CH_2), 4.53 (s, 2H, CH_2). ^{1}H NMR (400 MHz, 25:1 v/v DMSO-d_6/CF_3CO_2H): δ 10.14 (app. q, J_{app} = 6 Hz, 1H, N*H*, *Z* side), 9.88 (dt, *J* = 13, 6 Hz, 1H, N*H*, *E* side), 8.45 (dd, J = 13, 6 Hz, 1H, NC*H*N), 7.44–7.28 (m, 10H, Ph C*H*s),

4.70 (d, *J* = 6 Hz, 2H, C*H*$_2$), 4.60 (d, *J* = 6 Hz, 2H, C*H*$_2$). $^{13}C\{^1H\}$ NMR (101 MHz, DMSO-*d*$_6$) *δ* 155.43, 137.24, 135.40, 129.20, 129.13, 128.44, 128.40, 128.21, 128.18, 50.20, 45.60. HRMS (ESI) calcd for $C_{15}H_{17}N_2^+$ (M^+-I), 225.1386; found, 225.1427. Anal. Calcd. for $C_{15}H_{17}IN_2$: C, 51.15; H, 4.87; N, 7.95. Found: C, 51.19; H, 4.88; N, 7.93.

Table S4. Summary of Selected ^1H and ^{13}C (in parenthesis) Chemical Shifts for BnFAI and Bn_2FAI in *d*6-DMSO.

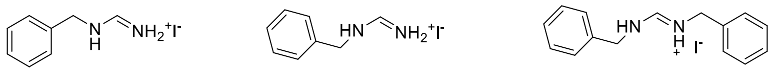

	Mono-		Bis-
	Minor	Major	
NH	9.31	9.31	9.93
NHC*H*NH	8.15 (158.07)	8.03 (154.98)	8.31 (155.43)
C*H*$_2$NH	4.54 (49.96)	4.50 (44.71)	4.64 (50.20)<br>4.53 (45.60)
*C*CH$_2$NH	-	- (135.56)	- (137.24)<br>- (135.40)

3.4.3 Solution NMR of Pre-synthesized BnFAI and Bn$_2$FAI

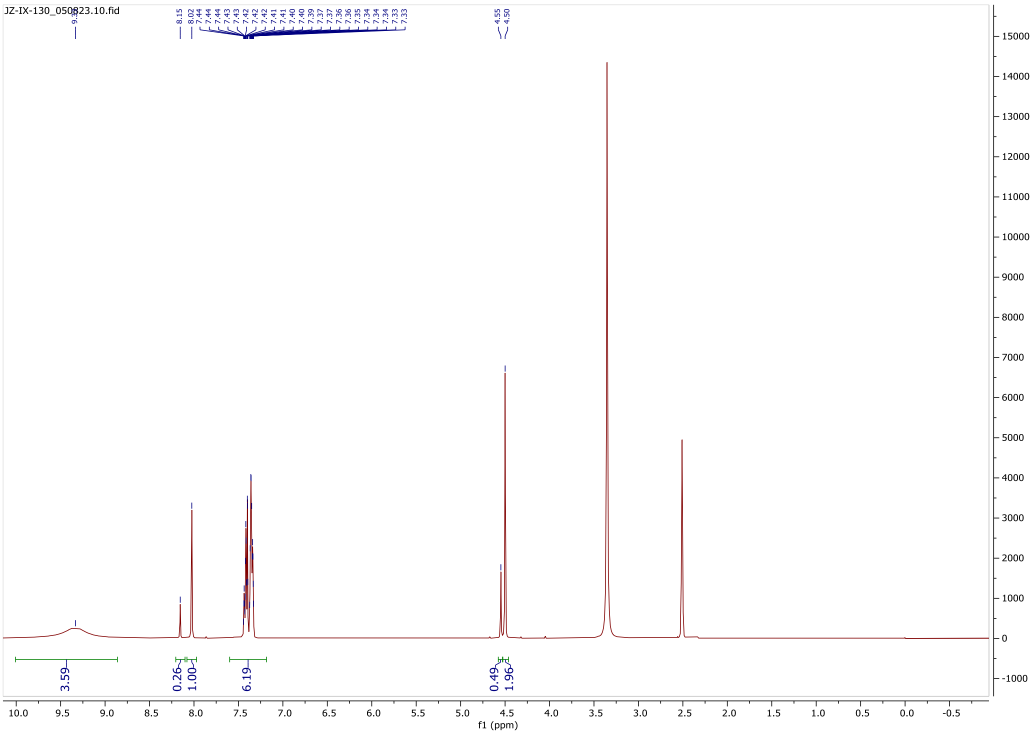

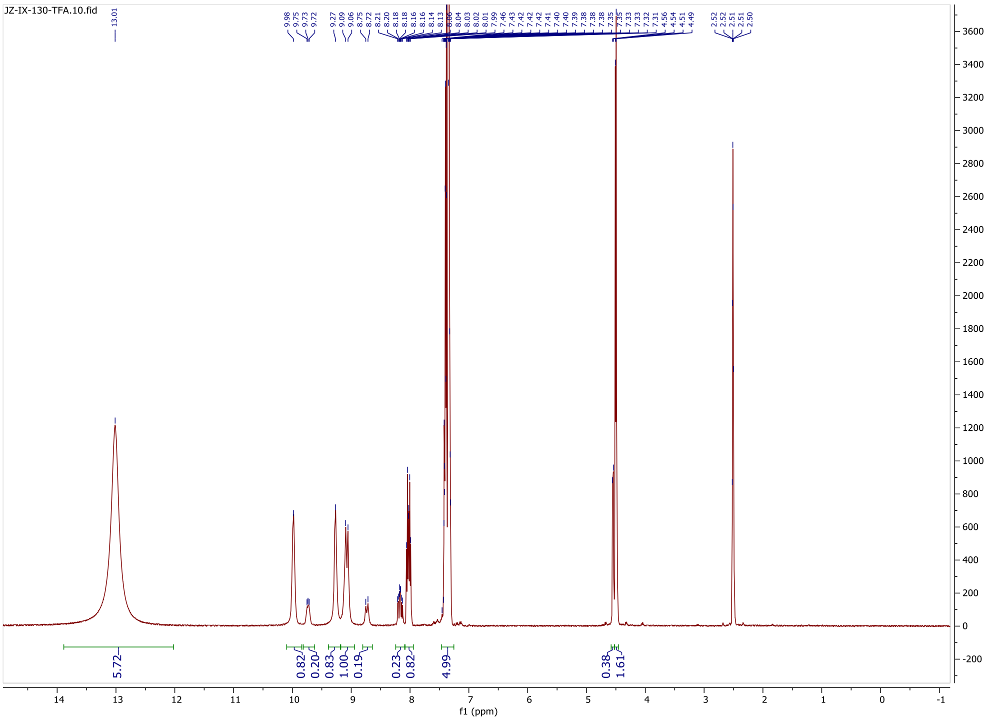

Supplementary Figure S18. ^{1}H NMR spectrum of *N*-benzyl formamidinium iodide in DMSO-d_6.

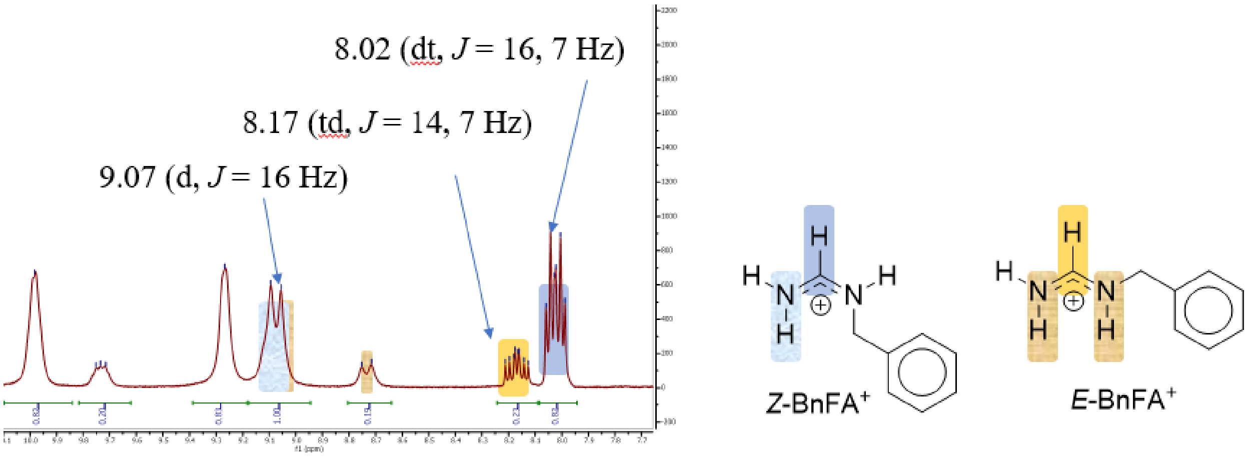

Supplementary Figure S19. ¹H NMR spectrum of *N*-benzyl formamidinium iodide in DMSO-*d*$_6$/CF_3CO_2H (25:1 v/v). Note that the formamidinium CH resonance of the major isomer appears as a dt (marked in blue); *i.e.*, exhibits one relatively large couplings to one proton and relatively small couplings to another two protons, consistent with the CH protons being *trans* to one NH proton and *cis* to two NH protons, *i.e.*, with this being the *Z*-isomer. Similarly, the minor isomer formamidinium CH resonance appears as a td with two large couplings and one small (marked in yellow), consistent with the CH protons being *trans* to two NH protons and *cis* to one NH proton, *i.e.*, with this being the *E*-isomer.

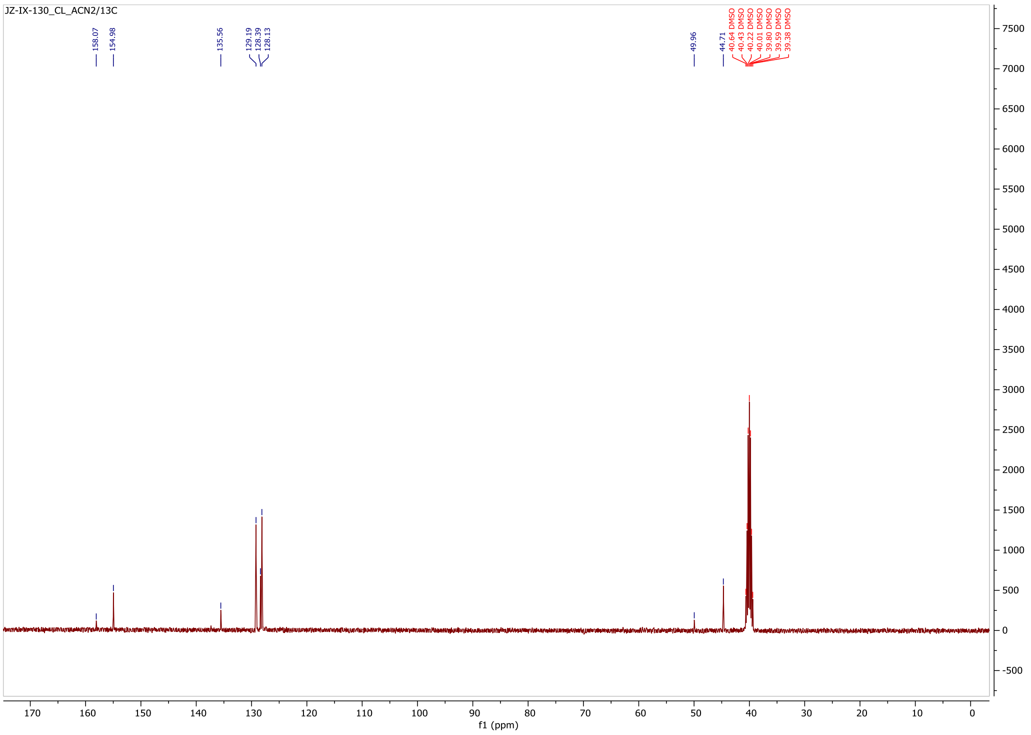

Supplementary Figure S20. $^{13}C\{^1H\}$ NMR spectrum of *N*-benzyl formamidinium iodide in DMSO-*d*$_6$.

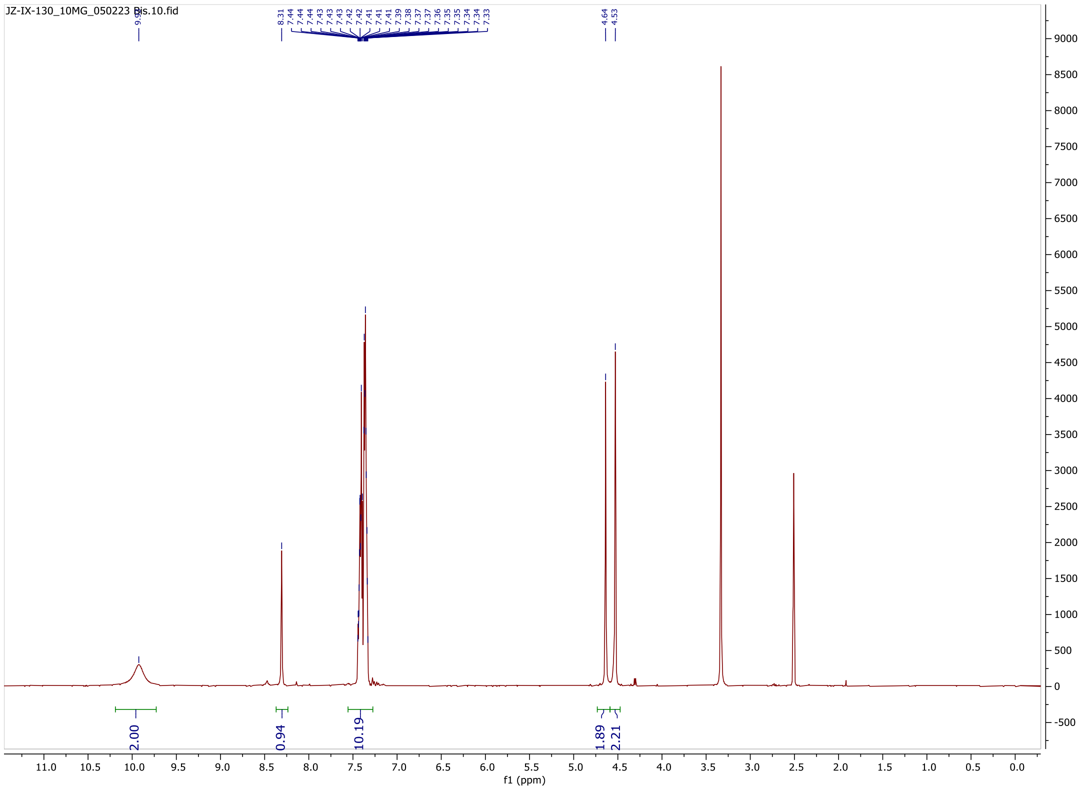

Supplementary Figure S21. ^{1}H NMR spectrum of *N,N'*-dibenzylformamidinium iodide in DMSO-d_6.

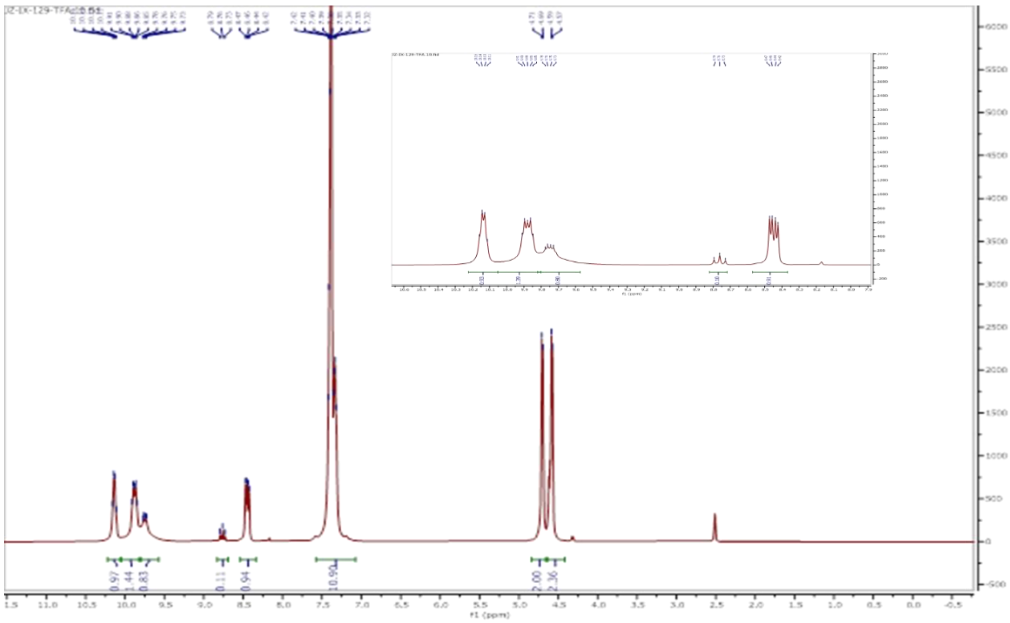

Supplementary Figure S22. ^{1}H NMR spectrum of *N,N'*-dibenzylformamidinium iodide in DMSO-d_6/CF_3CO_2H (25:1 v/v). The appearance of the central formamidinium CH resonance as a doublet of doublets (dd) with two different coupling constants is consistent with this being the *E*,*Z* isomer, in which the CH protons is *trans* to one NH proton and *cis* to another, ruling out the spectrum of **Supplementary**

Figure S21 representing a mixture of isomers with coincident CH resonances. The t at 8.76 ppm likely represents a small amount of the *E*,*E* isomer.

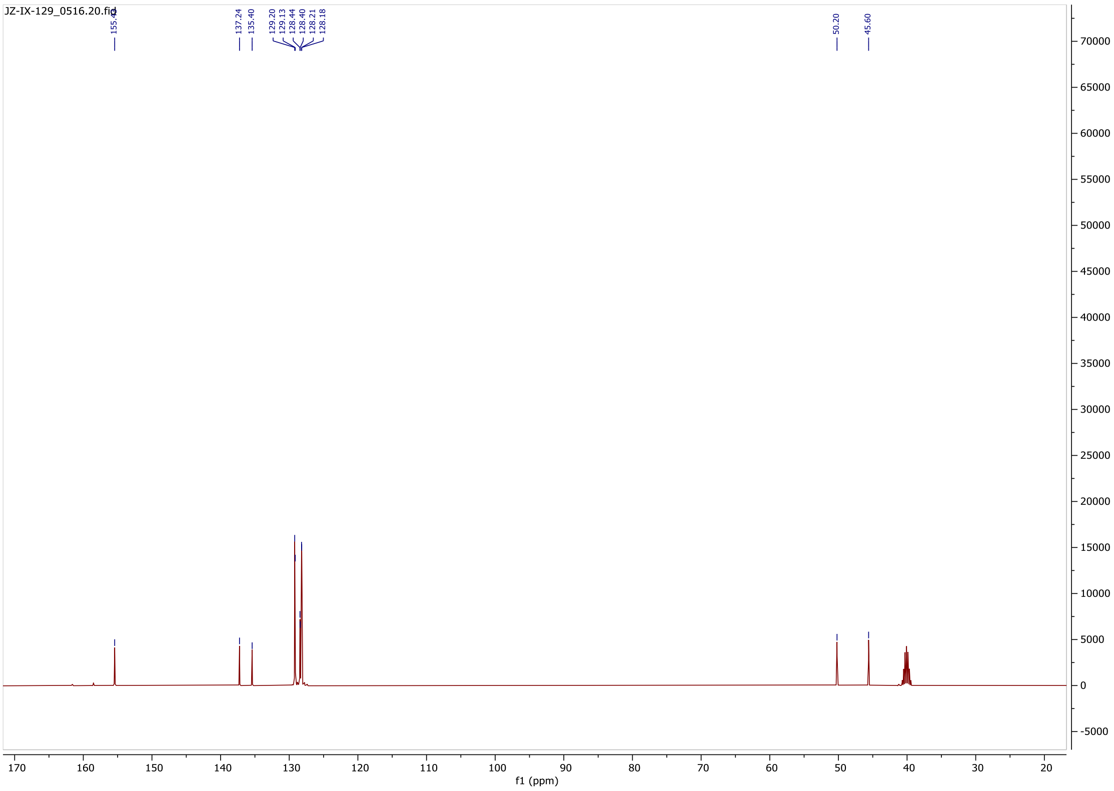

Supplementary Figure S23. $^{13}C\{^1H\}$ NMR spectrum of *N N'*-dibenzylformamidinium iodide in DMSO-d_6.

As shown in **Figure S24**, the isomers are stable in solution under elevated temperature, which suggests that they are likely to persist throughout the perovskite thin film.

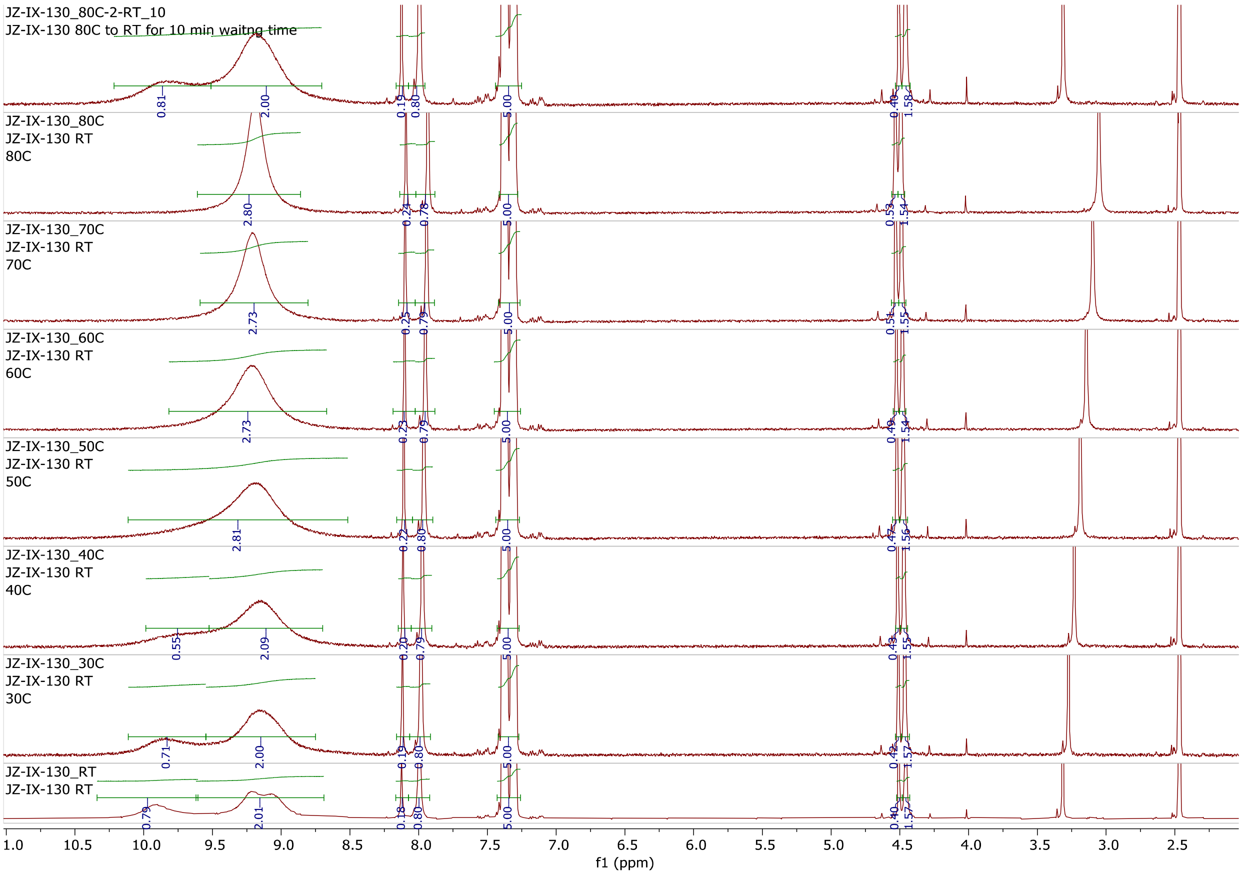

Supplementary Figure S24. ^{1}H NMR spectra of *N*-benzyl formamidinium iodide in DMSO-d_6 at temperature at (from bottom to top) room temperature (*ca*. 25 °C), 30 °C, 40 °C, 50 °C, 60 °C, 70 °C, 80 °C, and again at room temperature after cooling following heating. Note that the signals of the two isomers are neither broadened nor changed significantly in relative intensity on heating, implying slow interconversion. From the lack of broadening of the CH_2 signals (at ca. 4.5 ppm) we can infer a rate constant (k) of << 44 s^{-1} and a free-energy barrier (ΔG^*) >> 75 kJ mol^{-1} using the relation $k = \pi Dn/\sqrt{2}$ and the Eyring equations ($k = (k_B T/h)e^{-DG^*/RT}$). Note that there *are* exchange processes that affect the appearance of the signals from the NH and NH_2 protons at ca. 9-10 ppm.

3.5. Perovskite Phase and Morphology

3.5.1. SEM Images of Thin Films

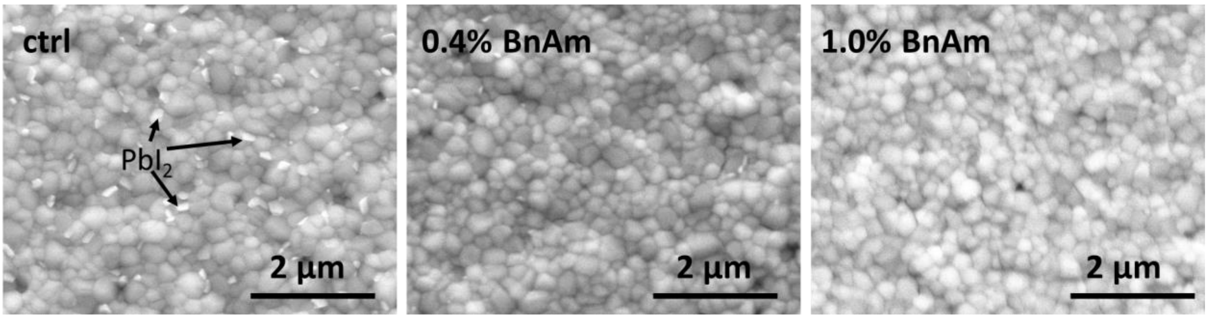

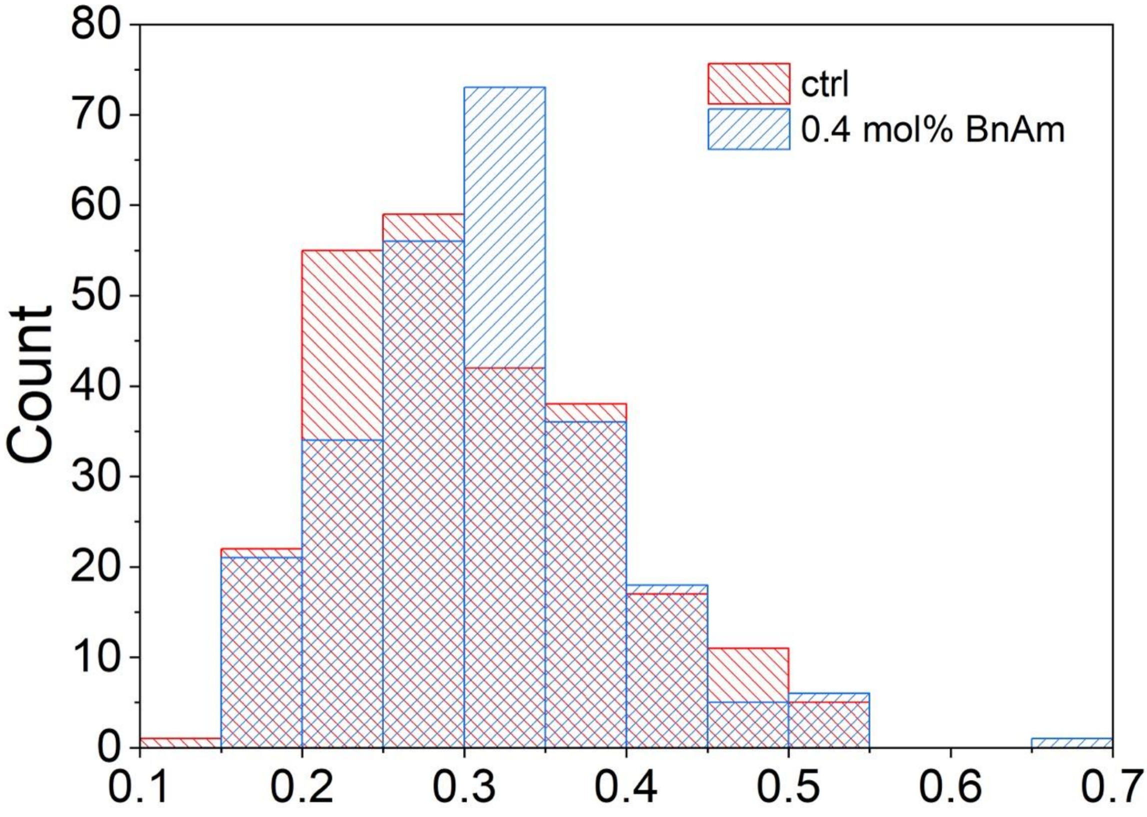

Supplementary Figure S25. Different mol% of BnAm additive in 1.68 eV $FA_{0.75}Cs_{0.25}Pb(I_{0.8}Br_{0.2})_3$ perovskite (1% excess PbI_2). PbI_2 formation in the control film is likely due to prolonged annealing on the hotplate at 100°C. Grain size distribution of the ctrl and 0.4 mol% BnAm additive samples above.

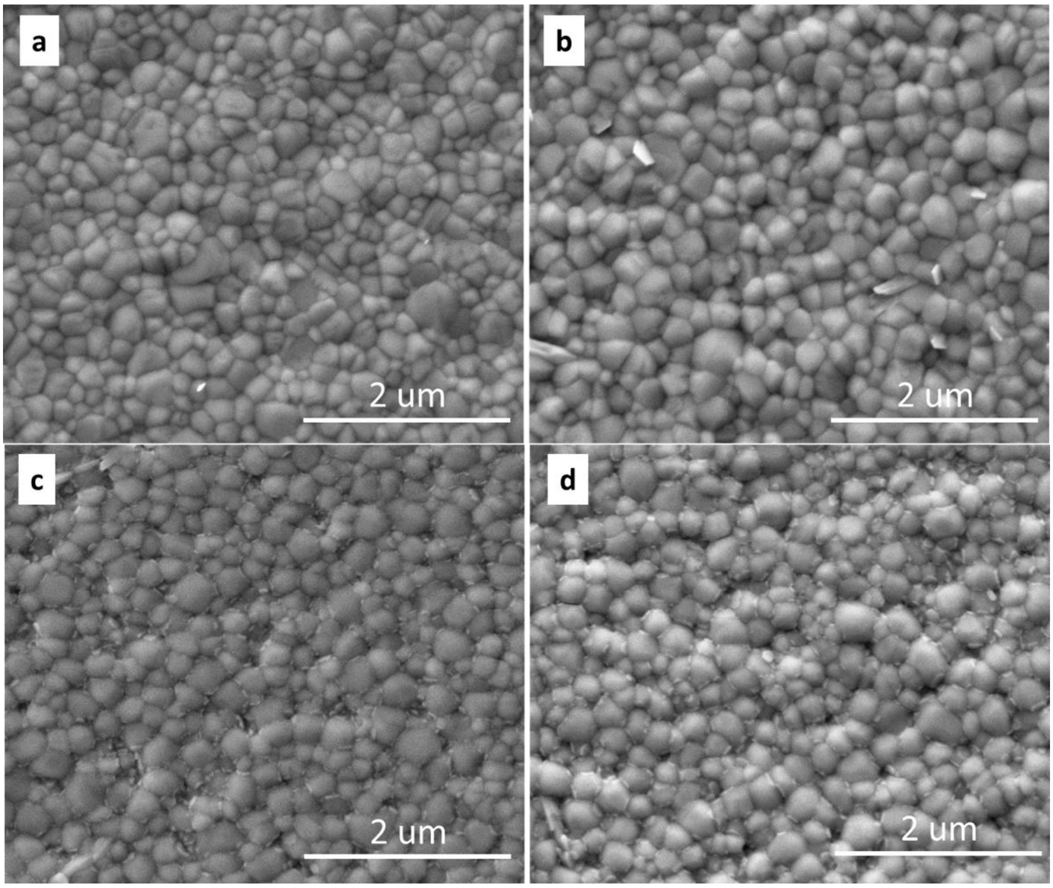

Supplementary Figure S26. 1.55 eV $FA_{0.8}Cs_{0.2}PbI_3$ (stochiometric) (a) ctrl film (b) $FA_{0.8}Cs_{0.2}PbI_3$ with 0.2 mol% BnAm additive. (c) $FA_{0.8}Cs_{0.2}PbI_3$ with 25 mM BnAm surface treatment (d) $FA_{0.8}Cs_{0.2}PbI_3$ with both 0.2 mol% BnAm additive and 25 mM BnAm surface treatment.

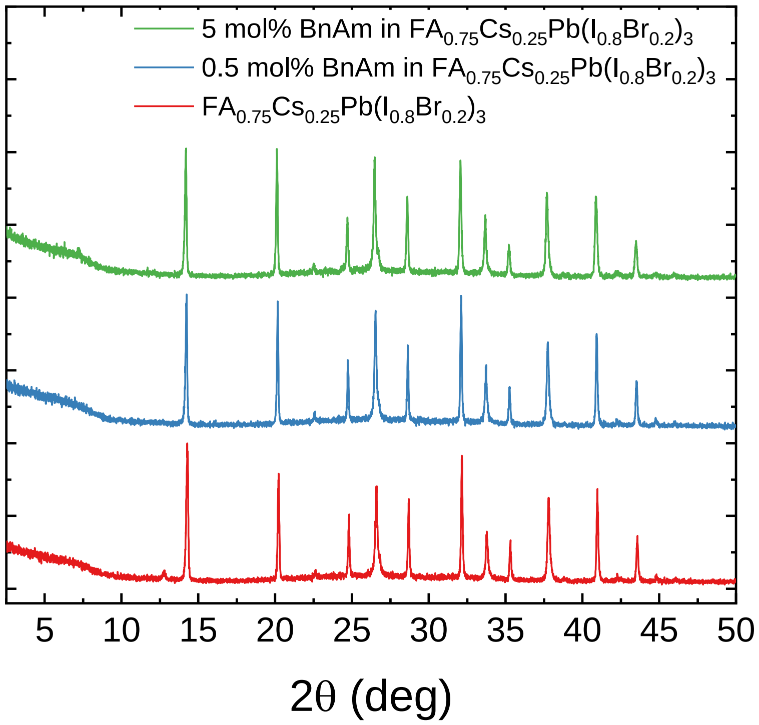

Supplementary Figure S27. XRD patterns of $FA_{0.83}Cs_{0.17}Pb(I_{0.75}Br_{0.25})_3$ perovskite films without additive, with 0.5 mol% BnAm additive, and with 5 mol% BnAm additive.

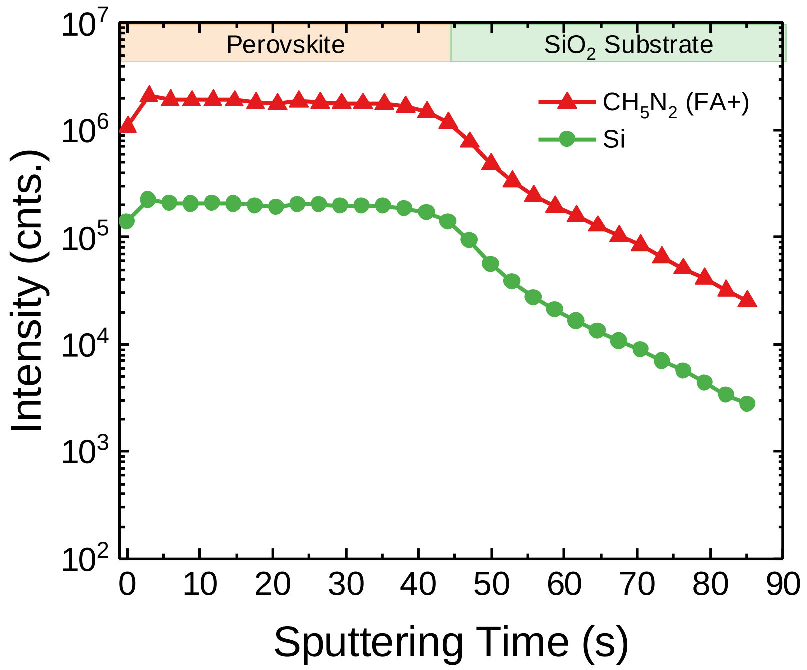

Supplementary Figure S28. ToF-SIMS molecular distribution of control perovskite film.

3.5.2. GIWAXS

To better understand the lower dimensional phases formed shown in **Figure S8**, we performed grazing-incidence wide-angle X-ray scattering (GIWAXS) measurements and found that a 5 mol% bulk additive and 3 vol% surface treatment (**Figure S29 c, e**) both resulted in a scattering ring at Q = 0.52 $Å^{-1}$, which was isotropic (unoriented) for the bulk additive, and more oriented with the surface treatment. In both cases, an out-of-plane scattering feature with a similar length scale to a benzylammonium n=1 Ruddlesden-Popper phase also emerged, with greater intensity after the surface treatment. Increasing the additive further to 10 mol% or the surface treatment to 5 vol% (**Figure S29 d, f**), and these peaks increase in intensity, with the surface treatment in particular resulting in a highly oriented phase with a significant number of Bragg spots. To confirm if this could be related to a reported BnAm phase, we simulated GIWAXS patterns for BnA_2PbI_4, $BnA_2MAPb_2I_7$ and $BnA_4Pb_5I_{14}\cdot 2H_2O$ (as reported in reference [9]) using *simDiffraction* [10] and found that clearly none of these phases could account for all of the observed reflections. Whilst we initially assumed that the new reflection at 7° in the XRD was from a second phase, along with BnA_2PbI_4, the clear observation of layer lines in the 5 vol% surface treatment (**Figure S29 f**) instead points to a single layered phase. This appears to have a comparable interplanar distance to BnA_2PbI_4 but reduced symmetry, resulting in periodic reflections in Qxy corresponding to a larger distance. We combined the peaks observed in GIWAXS together with 2D-XRD (**Figure S30**) and indexed these using GIDind,[11] finding a possible monoclinic unit cell with a = 8.71 Å, b = 22.21 Å and c = 28.79 Å, with β = 85.2°. The large length scale periodicity in the GIWAXS points to potentially a distorted or step-like layered phase, which cannot be accounted for by reported benzylammonium/FA phases. Such distortions and large unit cells have been reported due to octahedral distortions by disordered organic spacer layers[12], and so this observation is consistent with the phase including the $BnFA^+$ cation.

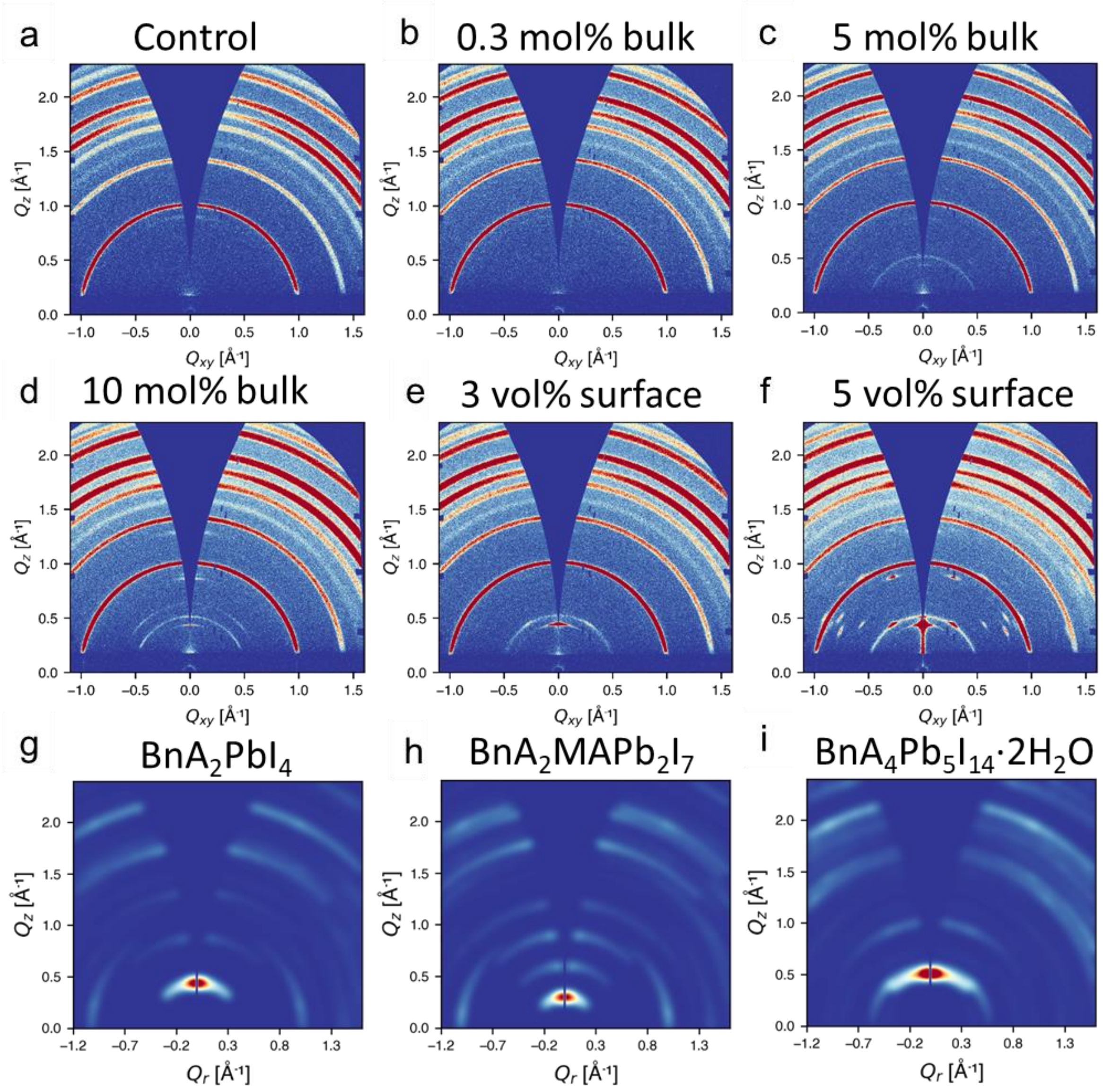

Supplementary Figure S29. GIWAXS pattern of (a) $FA_{0.75}Cs_{0.25}Pb(I_{0.8}Br_{0.2})_3$ perovskite ctrl film. (b) 0.3 mol% BnAm additive perovskite film. (c) 5 mol% BnAm additive perovskite film. (d) 10 mol% BnAm additive perovskite film. (e) 3 vol% BnAm perovskite surface treatment. (f) 5 vol% BnAm perovskite surface treatment. (g) simulated GIWAXS patterns of BnA_2PbI_4. (h) simulated GIWAXS patterns of $BnA_2MAPb_2I_7$. (i) simulated GIWAXS patterns of $BnA_4Pb_5I_{14}·2H_2O$ (CIF from ref [9]).

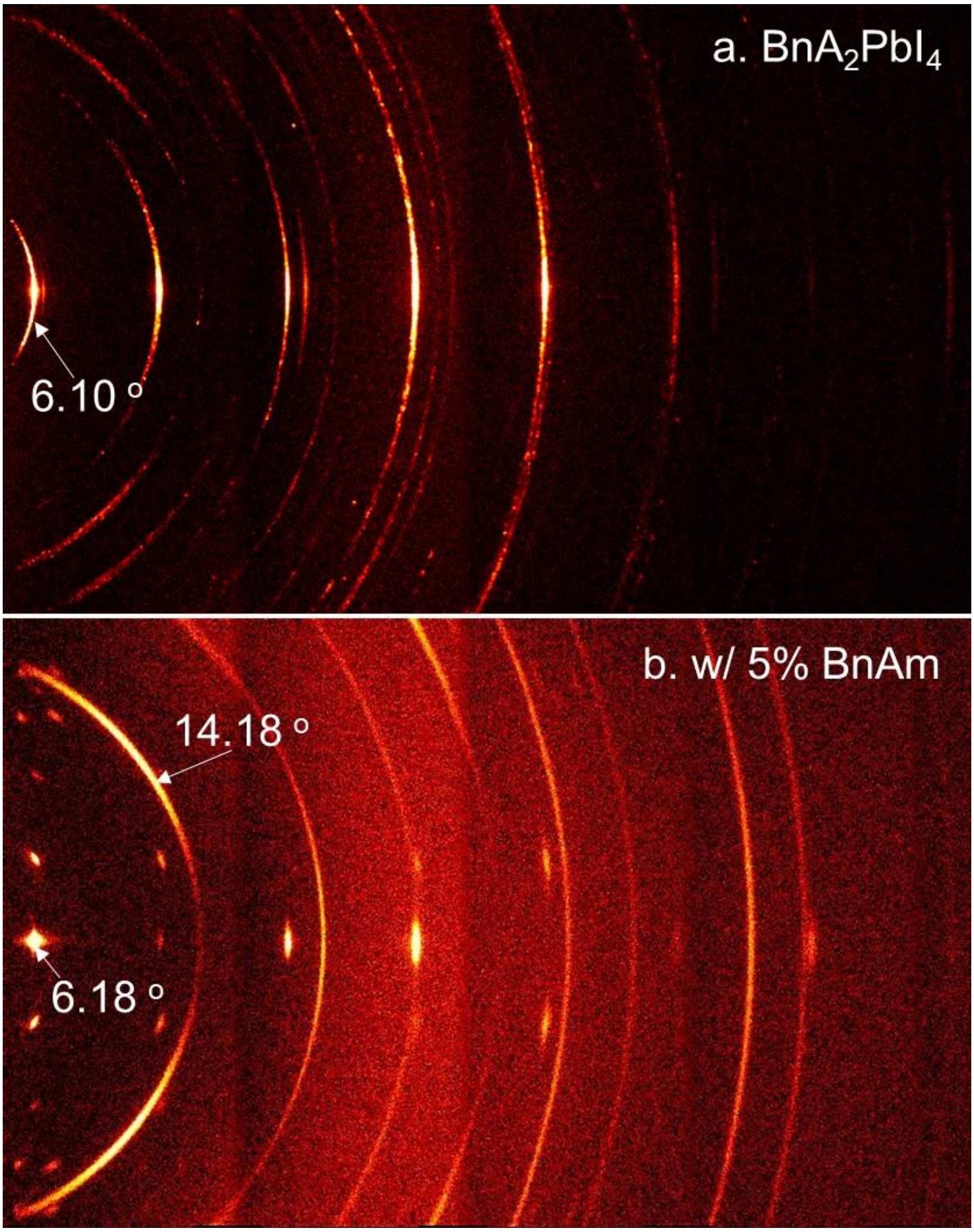

Supplementary Figure S30. (a) 2D XRD patterns of BnA_2PbI_4. (b) 2D XRD patterns of 5 vol% BnAm additive in $FA_{0.83}Cs_{0.17}Pb(I_{0.75}Br_{0.25})_2$, regular Q_z spacing of Q = 0.438 Å^{-1}, 1/Q= 14.36 Å, regular Q_{xy} spacing of Q = 0.271 Å^{-1}, 1/Q= 23.2 Å. The labels show the 2D phase patterns as well as the 3D perovskite pattern.

3.5.3. BnAm Additive at High Concentrations

In a pure $FAPbI_3$ perovskite, when we add high concentrations of BnAm (20 mol% and 50 mol%), the same lower-dimensional peaks appear at 2θ ~ 6.1° as in the

FACs- perovskite, from which we infer this phase does not include Cs^+ (see **Figure S31a**). However, when we add 20 mol% benzylammonium halides (BnAX, X = Cl, Br, I) to the perovskite, no lower-dimensional peaks are formed.

According to the solution NMR spectroscopy experiments, BnFAI and NH_3 are both formed as a result of BnAm and FA^+ reaction. Since BnFAI can form lower dimensional phases with PbI_2 that we correlate with the enhancement of the perovskite film quality, we pre-synthesized and isolated BnFAI to investigate its role further. In **Figure S32**, we show XRD patterns of the perovskite thin films formed when increasing concentrations of BnFAI are added to the pristine precursor ink, while reducing the original FAI content in the precursor ink by the same mol%. As BnFAI additive concentration is increased to 20 mol%, we observe formation of a lower dimensionality phase, which is in direct contrast to what we observed with the BnAX addition (**Figure S31b**). From this result, we infer that formation of the $BnFA^+$-containing lower dimensionality phase is more favorable than the corresponding benzylammnoium phase. This discovery may have implications for the mode of structural inclusion of these two bulky organic cations into the perovskite phase.

3.5.4. Comparison of Adding BnAm and BnFAI and BnAm Order of Addition

We investigate if BnFAI is solely responsible for the passivation effect at low additive concentrations relevant for device applications. BnFAI was added into the perovskite precursor solution at the optimal concentration for BnAm of 0.3 mol%, while the precursor FAI is reduced by 0.3 mol% to keep the FA^+ and I^- amount constant. By comparing the powder XRD patterns of BnFAI (**Figure S33**) with the BnFAI-modified perovskite films (**Figure S32**), it is apparent that we do not see the same diffraction patterns, which supports the notion that BnFAI is forming lower dimensional phases with PbI_2.

a

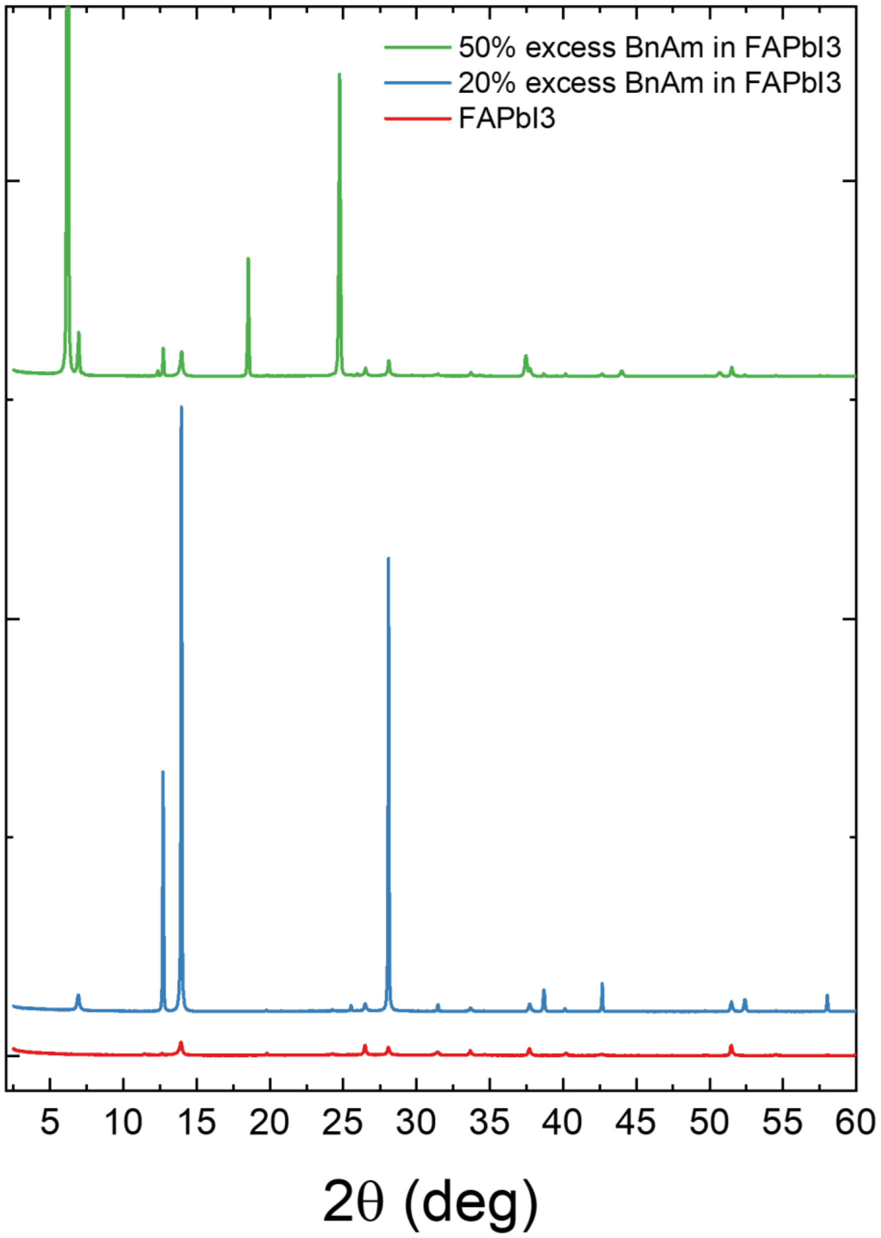

50% excess BnAm in FAPbI3
20% excess BnAm in FAPbI3
FAPbI3
5 10 15 20 25 30 35 40 45 50 55 60
2θ (deg)

b

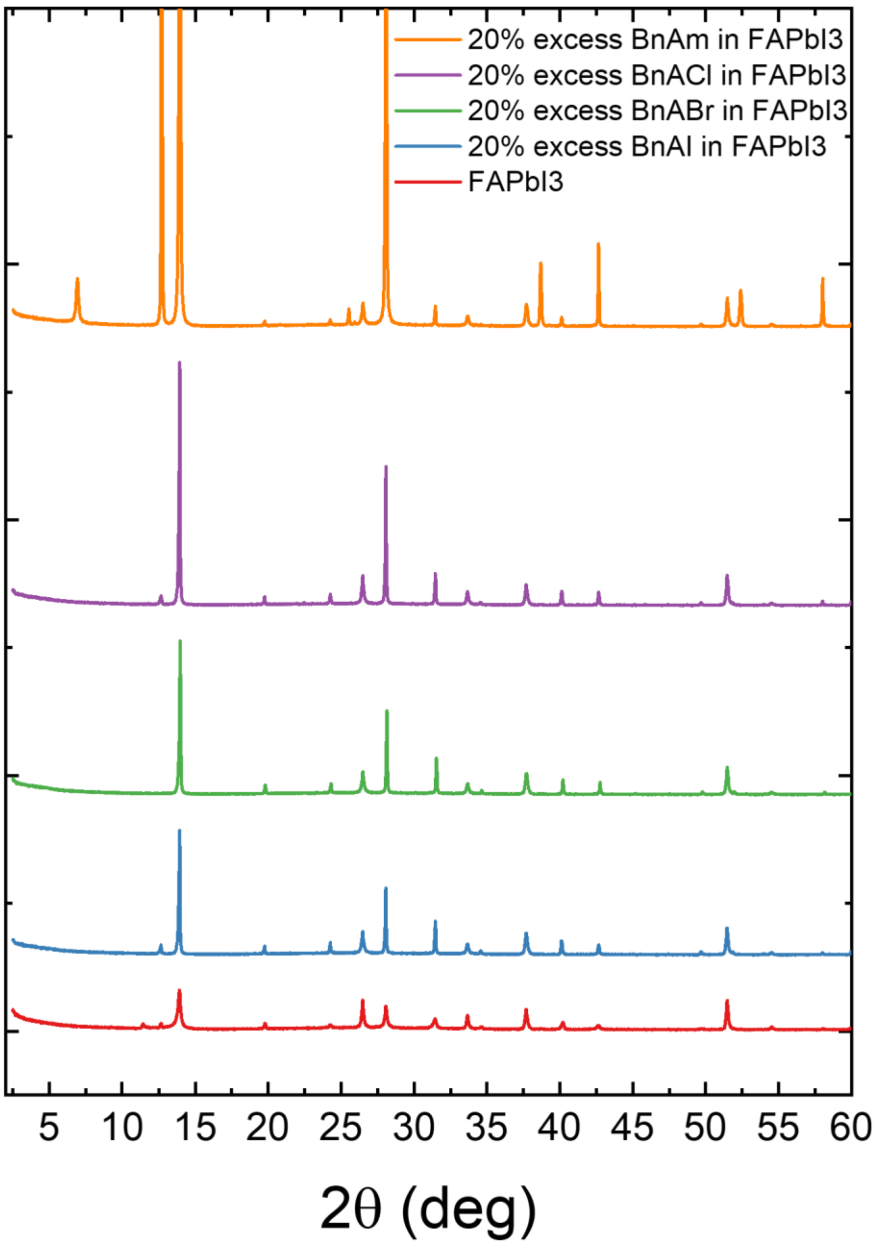

Supplementary Figure S31. (a) XRD of $FAPbI_3$ films with 20 mol% and 50 mol% excess BnAm addition. (b) $FAPbI_3$ film with 20 mol% excess of different benzylammonium halides (BnAX).

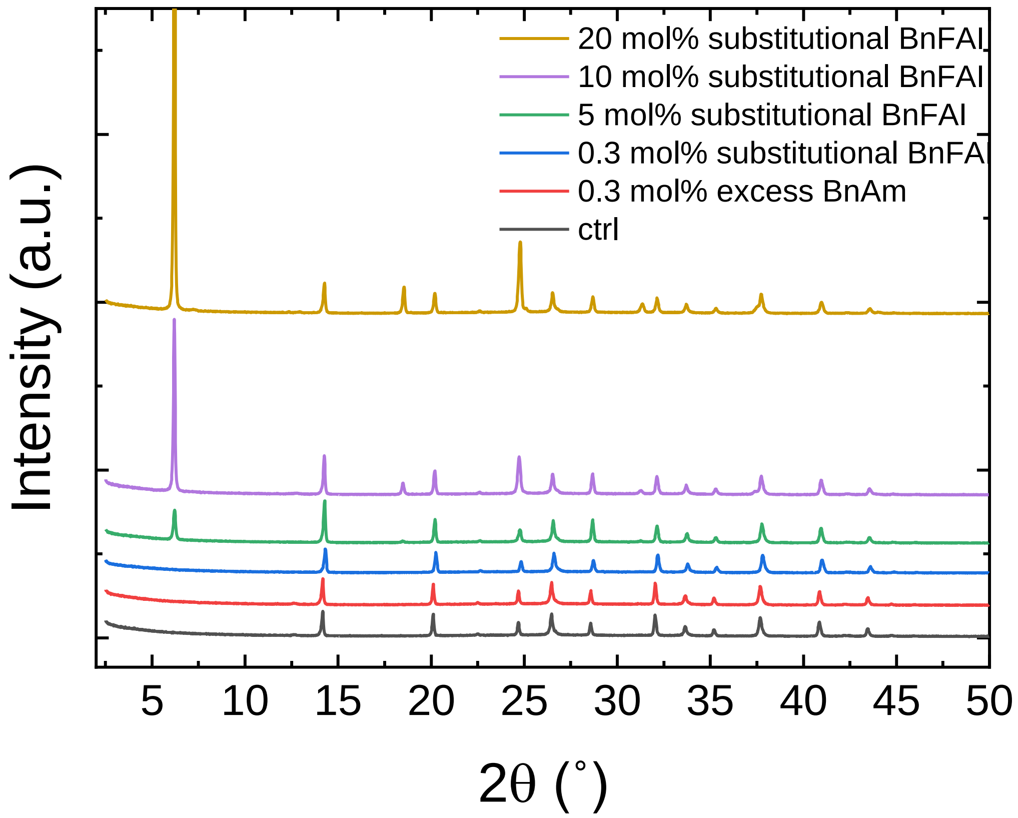

Supplementary Figure S32. XRD pattern of varying BnFAI additive concentration in the $FA_{0.75}Cs_{0.25}Pb(I_{0.8}Br_{0.2})_3$ perovskite films.

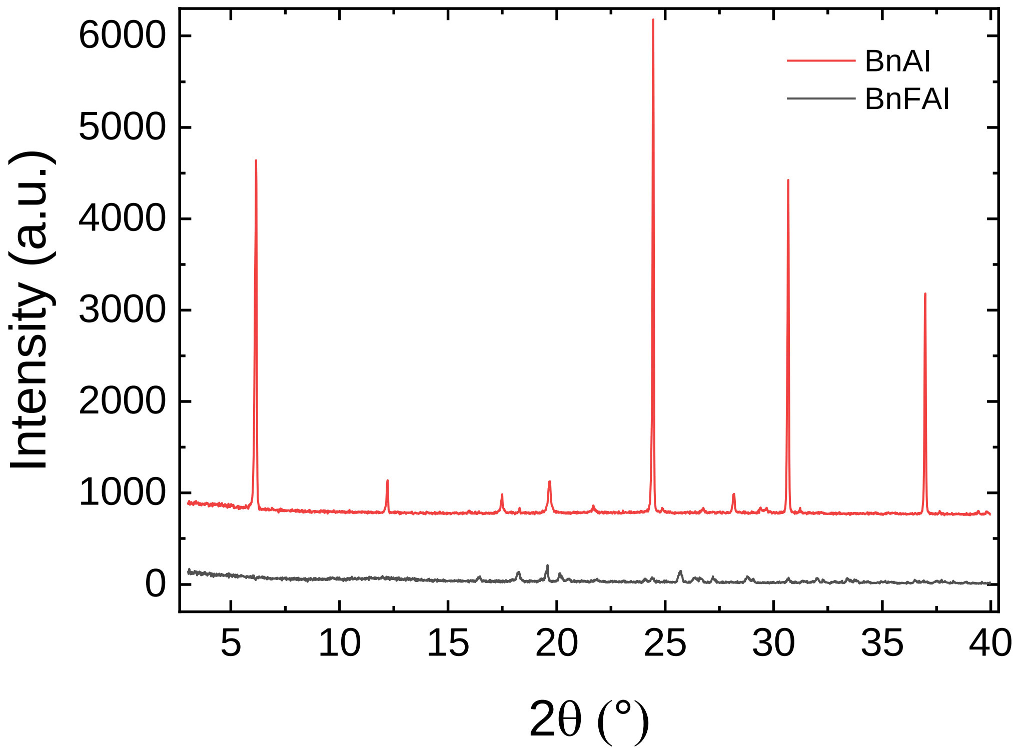

Supplementary Figure S33. Powder XRD patterns of pure BnAI and BnFAI.

Next, to check that in the case of directly adding BnFAI, the same crystallographic phases form as when BnAm is added, we compare adding 5 mol% BnAm and 5 mol% BnFAI additives in perovskite films, as shown in **Figure S34**.

We then compared the order of adding BnAm and its effects on the product. When BnAm is first mixed with FAI and then added to the rest of the perovskite precursor solution (**Figure S34** purple), we observe the lower-dimensional peak that corresponds to the BnFAI-modified perovskite at ~6.1˚. Along with the aforementioned ToF-SIMS results, we hypothesize that there are competing reactions between BnAm with FA^+ and FA^+-coordination to Pb^{2+}. When BnAm is added to the perovskite, NH_3 could coordinate with Pb^{2+}, leaving less available Pb^{2+} sites to coordinate with $BnFA^+$.

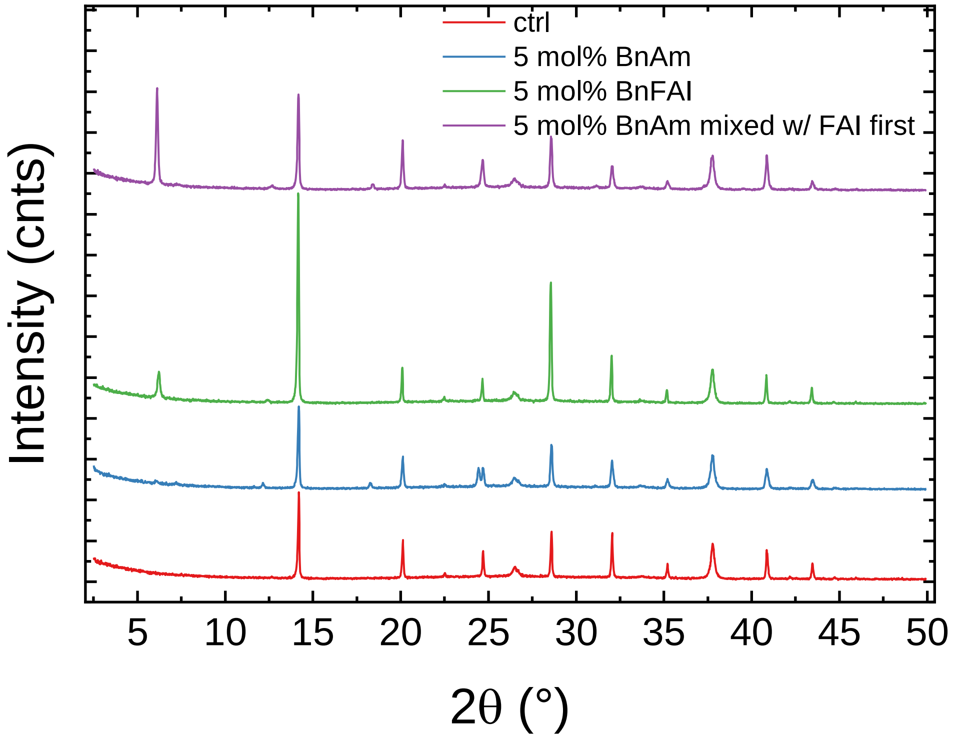

Supplementary Figure S34. XRD patterns of perovskite films: control film without additive, with 5 mol% BnAm, 5 mol% BnFAI, and 5 mol% BnAm mixed with FAI first and then with the rest of the perovskite.

3.6. Solid-State Nuclear Magnetic Resonance Spectroscopy

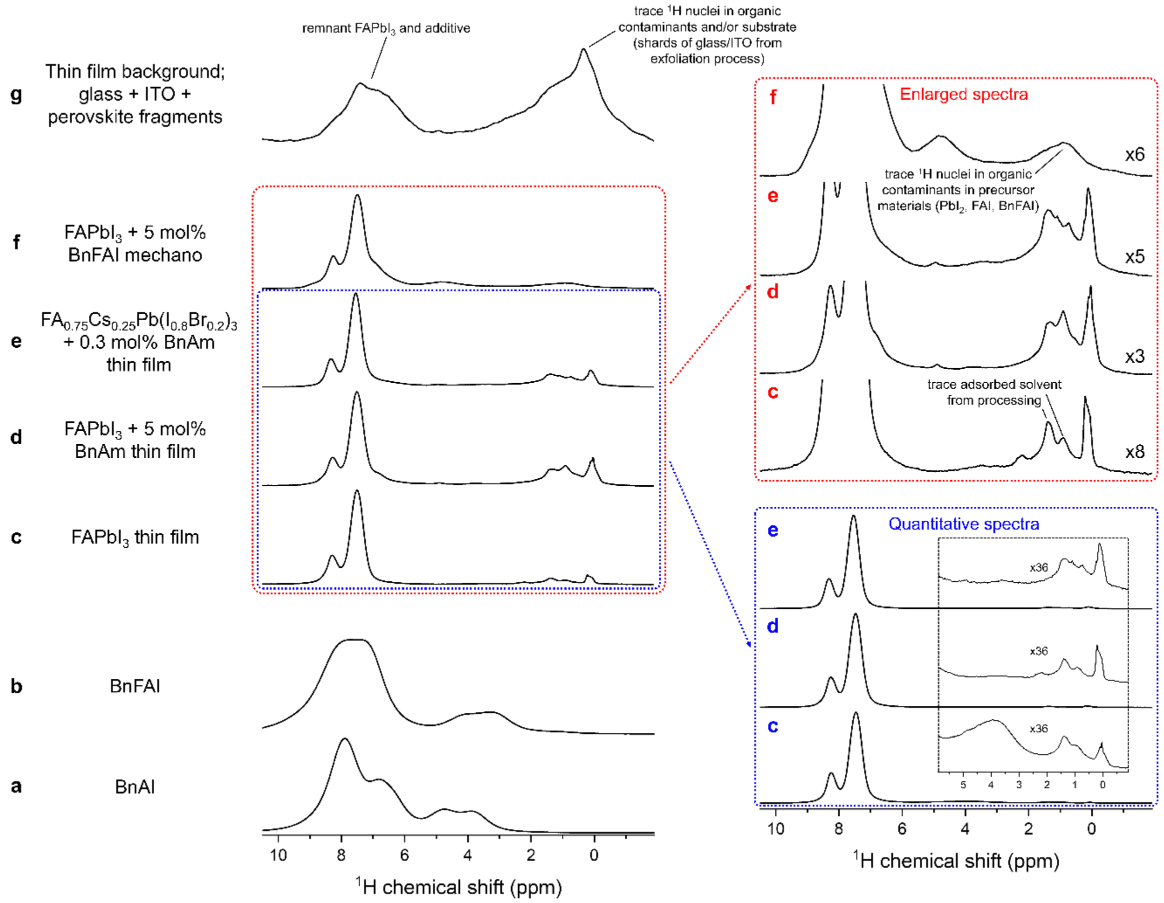

Supplementary Figure 35. Full ^{1}H ssNMR spectra, highlighting trace contaminants. ^{1}H MAS NMR (23.5 T, 55 kHz) spectra of (**a**) benzylammonium iodide (BnAI), (**b**) benzylformamidinium iodide (BnFAI), (**c**) $FAPbI_3$ thin films, (**d**) $FAPbI_3$ + 5 mol% BnAm thin films, (**e**) $FA_{0.75}Cs_{0.25}Pb(I_{0.8}Br_{0.2})_3$ + 0.3 mol% BnAm thin films, (**f**) $FAPbI_3$ + 5mol% BnAm fabricated mechanosynthetically by liquid-assisted grinding (LAG), (**g**) shards of mixed glass, ITO and perovskite recovered from samples after mechanical exfoliation. Spectra (**c-f**) (and enlarged versions highlighted in red) are recorded with short recycle delay to emphasize peaks corresponding to additives and contaminants, whose magnetization recovers more rapidly than that of FA^+. Quantitative spectra of (**c-e**) are recorded with a recycle delay of 150 seconds, to allow magnetization of all ^{1}H nuclei in the samples to fully recover and for direct quantitative comparison of these three spectra.

Note that the spectrum shown in **d** above is recorded with a much longer echo delay time (3640 μs; see **Figure S36**) than standard (114 μs) in order to emphasize the low intensity peak corresponding to $BnFA^+$ in the same environment observed in the target composition (**Figure S35e**).

In addition to the ^{1}H MAS NMR signals discussed in the main text, we observe several low intensity peaks at low chemical shift (-1 – 2.5 ppm). These are approximately consistent in quantity and lineshape (see **Supplementary Figure S35c-e** highlighted in blue) across all three solution-processed thin film samples. These signals do not correspond to expected environments in FA^+, BnA^+ or $BnFA^+$.

As shown in **Figure S35g**, we are able isolate some of the low chemical shift peaks (approximately -1 – 1 ppm) as originating from residual protons in the glass/ITO substrates. This is done by recording a spectrum on isolated shards of glass and ITO introduced into samples during thin film exfoliation process. In particular the feature centred at ~0.5 ppm, which is common across all our thin film material samples appears to originate from the substrate. We note that this seems to be a particular feature of substrates used in the corresponding author's laboratory; we have conducted similar analysis on substrates used by other groups and found no such residual ^{1}H signal.

We further note that the presence of a broad feature in the 1H MAS NMR of FAPbI3 + 5 mol% BnFAI mechano (**Figure S35f**) confirms that trace organic impurities are being introduced into the material via the precursors, most like from FAI, as highlighted in the figure. The only reagents for this mechanosynthesized material are PbI_2 (TCI, 99.99% trace metal basis), FAI (>99%, Greatcell Solar Materials) and BnFAI (synthesised in this work; see **Figure S18** for ^{1}H NMR spectrum). Given that the impurities seem to be present at comparable at% in the material as $BnFA^{+}$, and considering the purity of our synthesized BnFAI, it is unlikely that the synthesized material is the source of the impurities.

Besides the features noted above, we also note the presence of more clearly resolved (narrow) signals in the 1-3 ppm region of our thin film ^{1}H MAS NMR spectra. We tentatively suggest that these features may correspond to remnant processing solvents that are adsorbed to the perovskite material and survive post-processing thermal treatment.

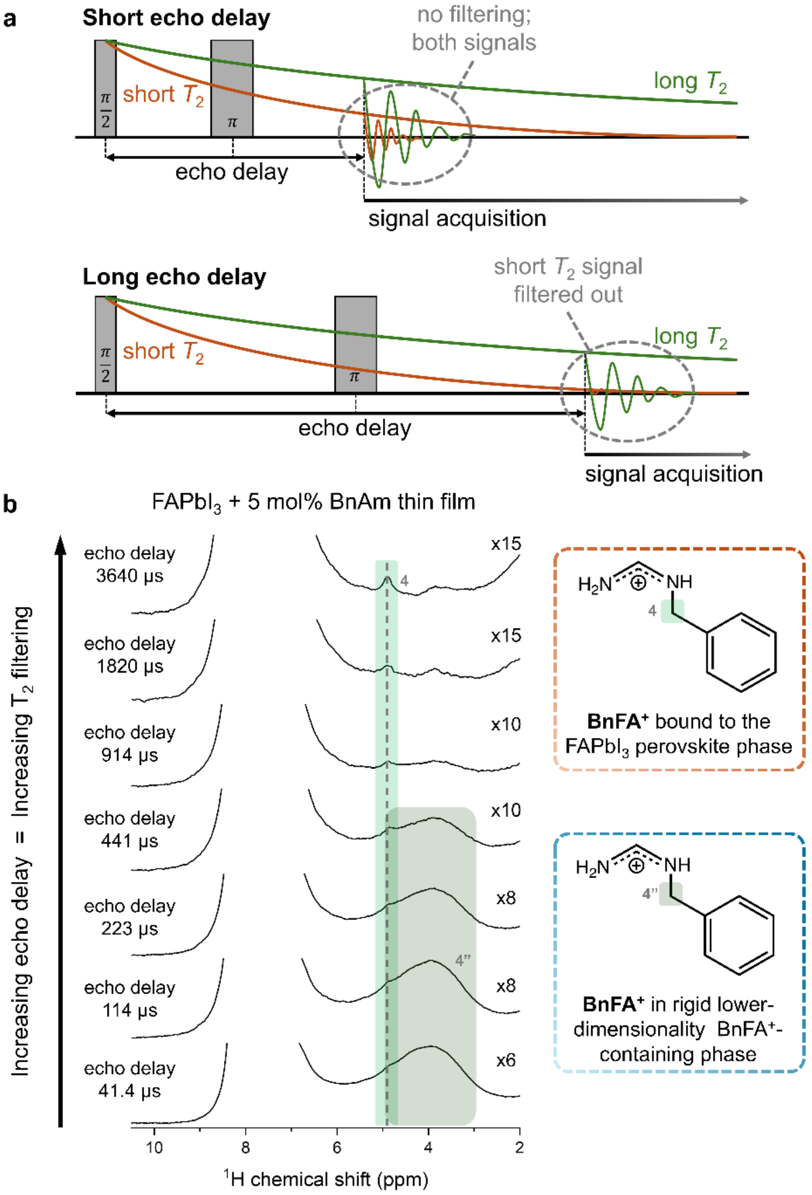

Supplementary Figure S36. Applying T_2 filtering to ^{1}H MAS NMR spectra to differentiate species by rigidity. a, NMR pulse sequence (Hahn echo) with short and long echo delay, highlighting the influence of altering echo delay on filtering of signals with short and long T_2. **b**, ^{1}H MAS NMR (23.5 T, 55 kHz) spectra of $FAPbI_3$ + 5 mol% BnAm thin film acquired *via* a Hahn echo pulse sequence with increasing echo delay.

Interactions between adjacent nuclear spins in the solid state lead to loss of magnetization over time in a process known as spin-spin relaxation. The rate of this loss of magnetization in this manner can be described by the spin-spin relaxation time, T_2. Spin-spin interactions within a material are suppressed by rapid molecular dynamics. Thus, components within a material that are relatively rigid (due to strong, well-defined intermolecular bonding, for example) typically display very short T_2 values. As shown in **Figure S36a**, by gradually increasing the spin-echo delay time it is

possible to ‘filter-out’ peaks corresponding ^{1}H nuclei in more rigid environments (short T_2) from ^{1}H MAS NMR spectra. Nuclei with longer T_2 values are unaffected.

By applying this method to our $FAPbI_3$ + 5 mol% BnAm thin film sample (**Figure S36b**) deduce the following:

1) The same ^{1}H signal (4.90 ppm) is present in this sample as in our device composition thin film (**Figure 3b**), corresponding to $BnFA^+$ in atomic-level proximity to FA^+ in $FAPbI_3$ (see main text).
2) The smaller, narrower signal at 4.90 ppm corresponding to $BnFA^+$ in contact with the 3D perovskite phase clearly has a substantially longer T_2, suggesting these cations are relatively dynamic in the structure in comparison to those in the suspected 2D phase. This is also consistent with the absence of well-defined intermolecular bonding between neighbouring $BnFA^+$ bound to the 3D perovskite surface, which are presumably somewhat more dispersed than those in the 2D phase, prohibiting intermolecular interactions and allowing less-inhibited molecular dynamics.
3) The presence of the broad, T_2-filterable peak at ~4 ppm suggests that this thin film sample (which has substantially higher BnAm additive loading than our other thin film samples) has an additional species in it. Considering the efficacy of our T_2 filter, it is clear that this species has a very short T_2 time, suggesting that it is bound rather rigidly inside the material. Given the chemical shift of this peak, and our GIWAXS results on this composition that clearly show the formation of 2D perovskite-like phases, the most likely interpretation of this peak is that it corresponds to the methylene ^{1}H nuclei of $BnFA^+$ in a lower dimensionality low-*n* perovskite phase, for example of the $(BnFA)_2FA_{n-1}Pb_nI_{3n+1}$ Ruddlesden Popper family. The rigidity of the cation could also therefore be accounted for by strong π-π bonding between adjacent $BnFA^+$ cations in the 2D perovskite phase.

Taken together, these results suggest that two modes of $BnFA^+$ incorporation are active in our materials; (1) $BnFA^+$ capping 3D perovskite domains, and (2) $BnFA^+$ forming isolated low-dimensionality phases. As at low $BnFA^+$ concentrations (<0.5 mol%, **Figure 3b**) only the domain-capping mode is observed, it seems that this is the energetically preferred mode. However, when $BnFA^+$ concentration is increased, an

additional incorporation mode becomes active; formation of isolated secondary phases of low-dimensionality materials (see XRD [**Figure S32**], GIWAXS [**Figure S29**] and ^{1}H MAS NMR [**Figure S35**]). In order to unify the results of our high and low $BnFA^{+}$ concentration materials, we show this model schematically in **Figure 3h**.

Finally, we note that further to the arguments presented in the main text in support of the domain surface passivation model for $BnFA^{+}$ incorporation, the absence of ^{1}H signals corresponding to $BnFA^{+}$ in an isolated phase in the liquid-assisted grinding material (**Figure 3a** and **3f**), in comparison to the corresponding 5 mol% BnAm thin film material (**Figure S35-36**), also supports this model. Mechanosynthesis typically produces materials with substantially smaller domain sizes and greater disorder than polycrystalline thin films. The density of crystallite surface area in the LAG material, with which $BnFA^{+}$ is hypothesized to bind, is therefore much greater than in the corresponding thin film material. Thus, it is expected that a greater proportion of the $BnFA^{+}$ introduced to the material is utilized in passivating perovskite crystallite surfaces. Our ^{1}H NMR results suggest that all $BnFA^{+}$ present in the liquid-assisted grinding material is passivating in this fashion (**Fig. 3f** and **Figure S35**). Not only does this result support our hypothesized binding mode, but it justifies the use of the liquid-assisted grinding material as representative of the device composition perovskite.

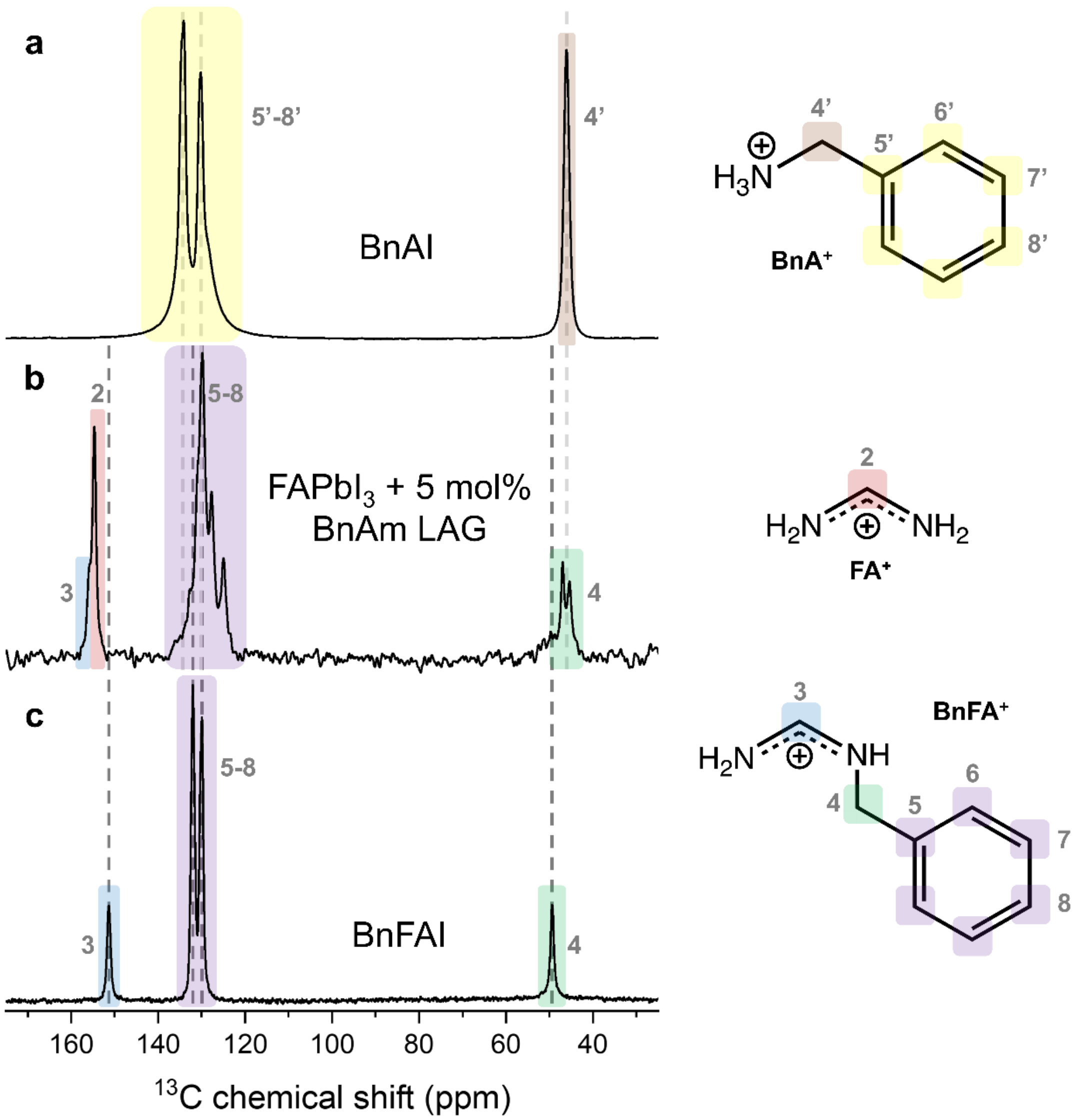

Supplementary Figure S37. Comparing ^{13}C MAS NMR spectra of additive-loaded $FAPbI_3$ with suspected incorporated additives. ^{13}C MAS NMR (11.7 T, 15 kHz) spectra of $FAPbI_3$ + 5mol% BnAm fabricated mechanosynthetically by liquid-assisted grinding (LAG) (**b**) compared with benzylammonium iodide (BnAI, **a**) and benzylformamidinium iodide (BnFAI, **c**). All spectra are acquired *via* ^{1}H-^{13}C cross-polarization (CP).

3.7. Perovskite Solar Cells

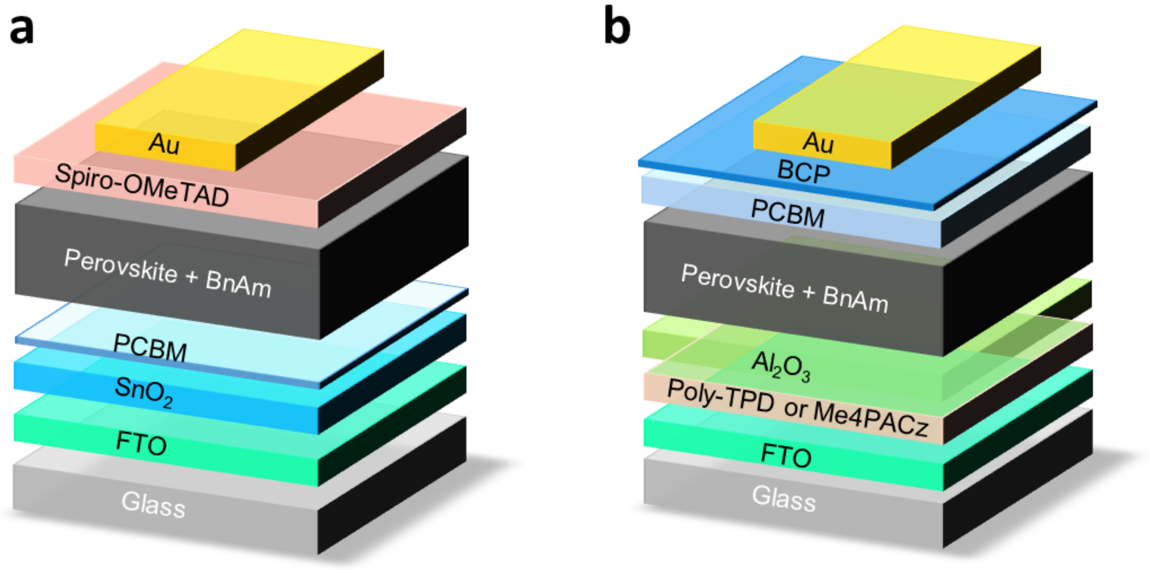

Supplementary Figure S38. (a) n-i-p device stack. (b) p-i-n device structure used for both medium and wide bandgap MA-free perovskite solar cells.

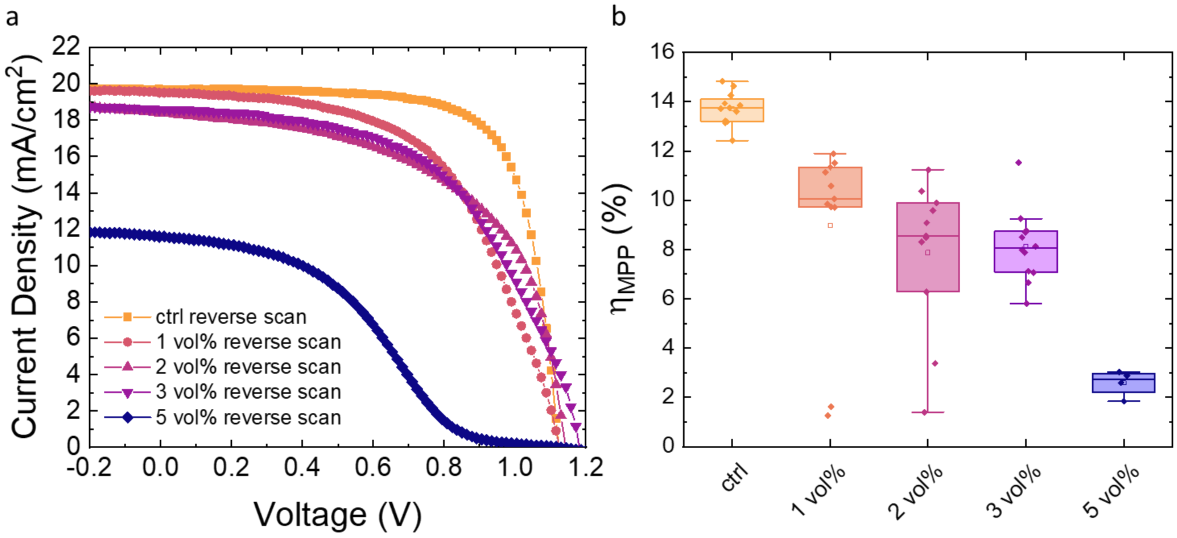

Supplementary Figure S39. Different volume percent surface treatment of BnAm (in IPA) on the $FA_{0.75}Cs_{0.25}Pb(I_{0.8}Br_{0.2})_3$ n-i-p device performance. (a) J-V curves of devices with different vol% BnAm surface treatment. (b) Maximum power point tracked (for 30 seconds) efficiency of these devices.

3.7.1. n-i-p Perovskite Solar Cells

Having established an enhancement of the perovskite film optoelectronic quality with the addition of 0-1 mol% BnAm, we fabricate negative-intrinsic-positive (n-i-p) perovskite solar cells (PSCs) with BnAm additive. The architecture is FTO/SnO_2/PCBM/Perovskite/Spiro-OMeTAD/Au with a perovskite absorber of $FA_{0.75}Cs_{0.25}Pb(I_{0.8}Br_{0.2})_3$. **Figure S38a** shows a schematic of the device architecture. With 0.3 mol% BnAm additive, the average V_{oc} increases from 1.07 ± 0.04 V to 1.09 ± 0.04 V (**Figure S40a**), consistent with the improvement in PLQY (**Figure 1b**). The average fill factor is slightly improved from 67.9 ± 7.2% to 72.7 ± 4.5% and the average J_{sc} also improved from 18.29 ± 1.03 mA/cm^2 to 18.65 ± 0.77 mA/cm^2 with 0.3 mol% BnAm addition (**Figure S40b and S40c**). The improvements in all device parameters contribute to the increase in the average maximum power point tracked efficiency (η_{MPP}) from 14.5% ± 1.0 to 15.6 ± 1.3% (**Figure S40d**).

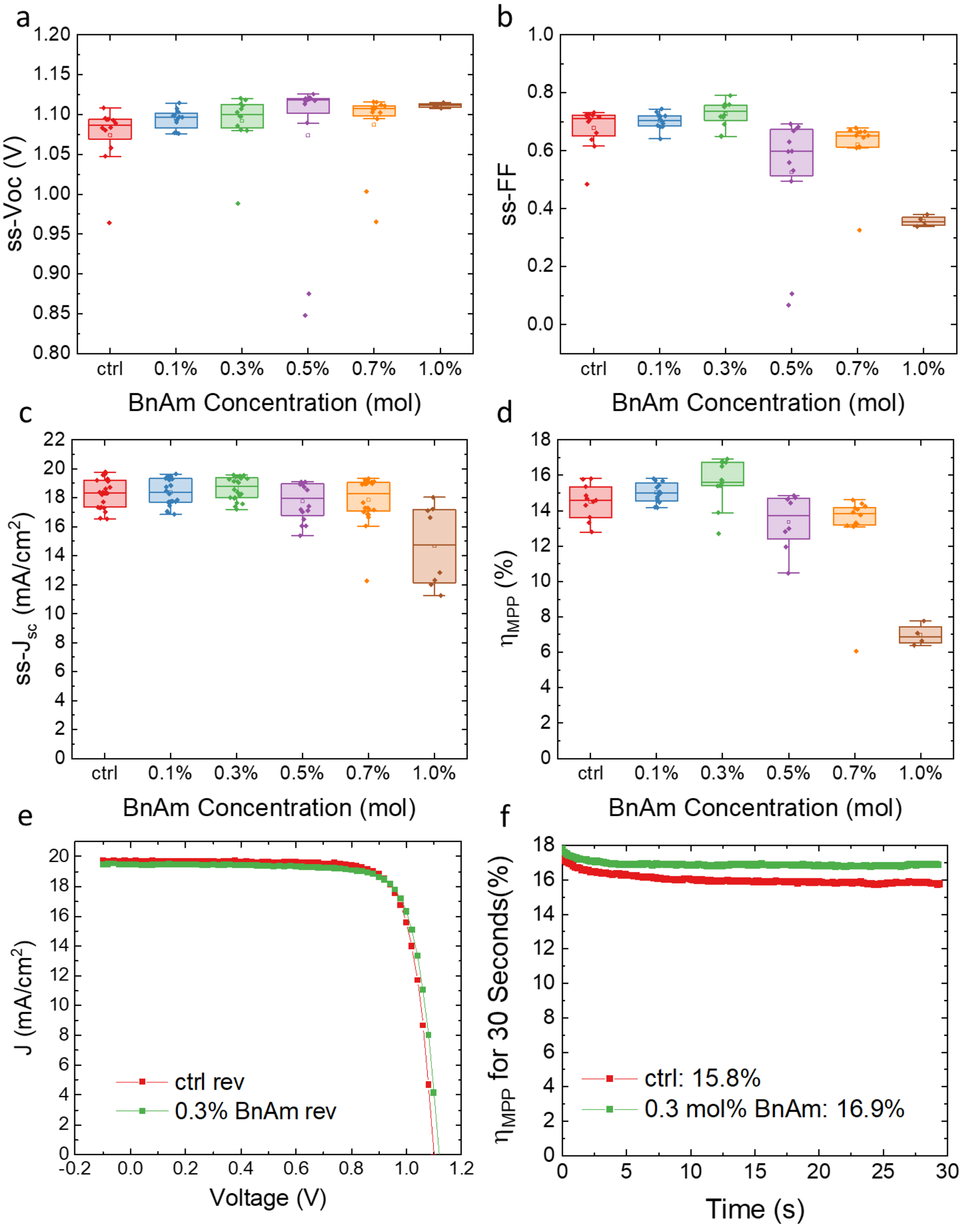

Supplementary Figure S40. Device parameters of different concentrations of BnAm additive in n-i-p devices. (a) Steady-state open-circuit voltage, stabilized for 30 seconds. (b) Steady-state fill factor, stabilized for 30 seconds. (c) Steady-state short circuit current density, stabilized for 10 seconds. (d) n-i-p device statistics of maximum power point tracked efficiencies (η_{MPP}) with different amounts of BnAm additive for the 1.68 eV $FA_{0.75}Cs_{0.25}Pb(I_{0.8}Br_{0.2})_3$ perovskite. (e) J-V curves of n-i-p champion devices with and without BnAm additive in the $FA_{0.75}Cs_{0.25}Pb(I_{0.8}Br_{0.2})_3$ perovskite. (f) 30 seconds of maximum power point tracked efficiency of the devices in (e).

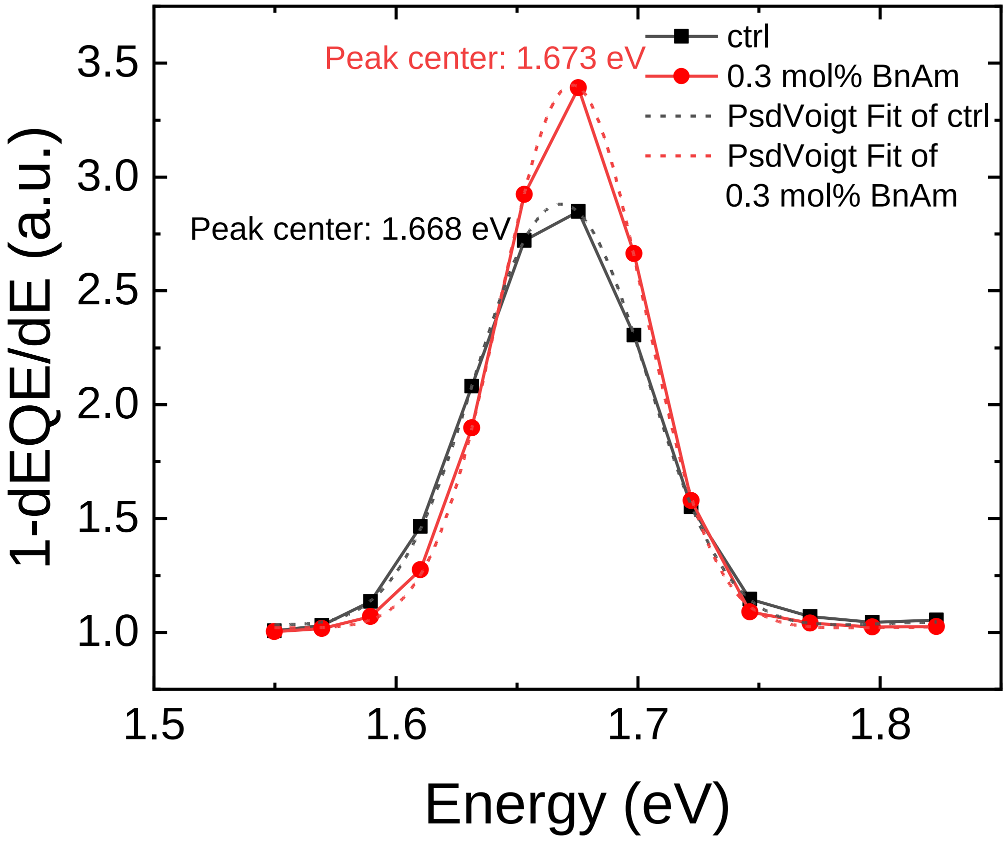

Supplementary Figure S41. Photovoltaic bandgap determination of $FA_{0.75}Cs_{0.25}Pb(I_{0.8}Br_{0.2})_3$ n-i-p devices with and without 0.3 mol% BnAm additive through dEQE/dE analysis. Both devices with and without BnAm additive exhibit a peak at 740 nm. We use 1-dEQE/dE to turn the peaks signs positive. Their PV bandgaps are then calculated by: $E = h\nu = hc/\lambda \approx 1.68$ eV.

3.8. BnAm and BnFAI comparison in MA-free perovskite solar cells

We hypothesize that directly adding BnFAI instead of BnAm would be a more controllable means to add the active component to the solution. To investigate whether the substitution of BnFAI for FAI has the same effect on perovskite devices as adding BnAm at 0.3 mol%, we fabricated p-i-n perovskite solar cells with the architecture: FTO/Me4PACz/Al_2O_3 nanoparticles/$FA_{0.75}Cs_{0.25}Pb(I_{0.8}Br_{0.2})_3$/PCBM/BCP/Au (**Figure S38 b**). The FA^+/$BnFA^+$ stoichiometry is kept constant by reducing FAI in the precursor by 0.3 mol%. The device parameters are shown in **Figure 42 a-d**. While BnFAI-modified devices have the same steady-state V_{oc} and J_{sc} boost as BnAm-modified

devices, their steady-state FFs are slightly lower, but still resulting in a boost in η_{MPP} compared to the control devices.

Next, we compare the influence of varying the point at which BnAm is added to the perovskite solution upon film properties and devices. When BnAm is reacted with FAI first and then added into the perovskite precursor solution, the device FFs are also slightly worse than the BnAm-modified devices and comparable to the device FFs of direct BnFAI addition (see **Figure 42a**), as would be expected from the XRD results. The higher FFs for BnAm-modified devices can be possibly attributed to Pb^{2+} coordination with NH_3 according to the ToF-SIMS results. Note that benzylamine is a Lewis base for PbI_2 so the presence of Pb^+ can also affect the pK_a of the solution and the amount of $BnFA^+$ formed as well.

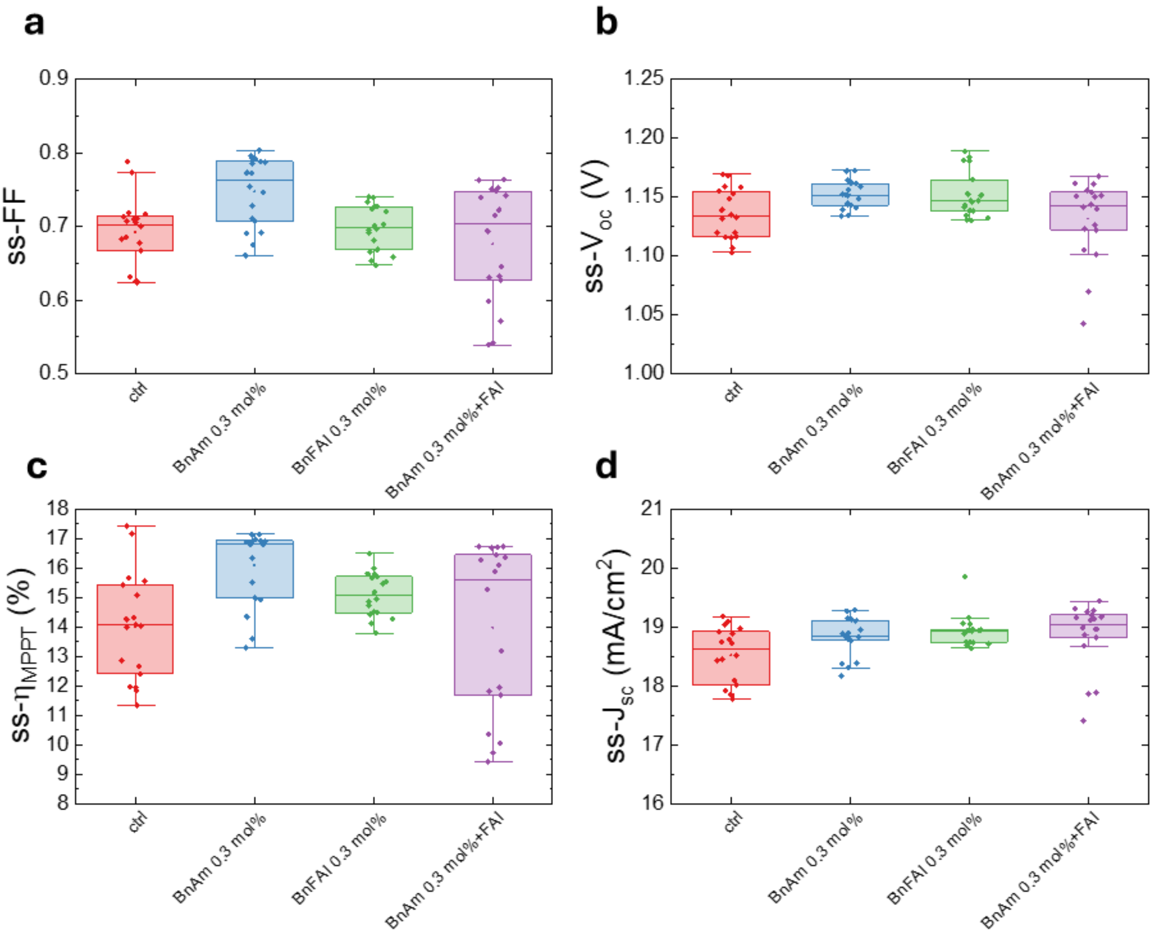

Supplementary Figure S42. (a) Steady-state (ss) fill-factor (ss-FF). (b) 30 seconds of ss-open-circuit voltage (ss-V_{oc}). (c) 30 s of maximum power point (η_{MPP}) tracking efficiency of devices (d) 10 seconds of ss short-circuit current-density (ss-J_{sc}) when BnFAI is directly applied in comparison to applying BnAm and forming BnFAI in-situ.

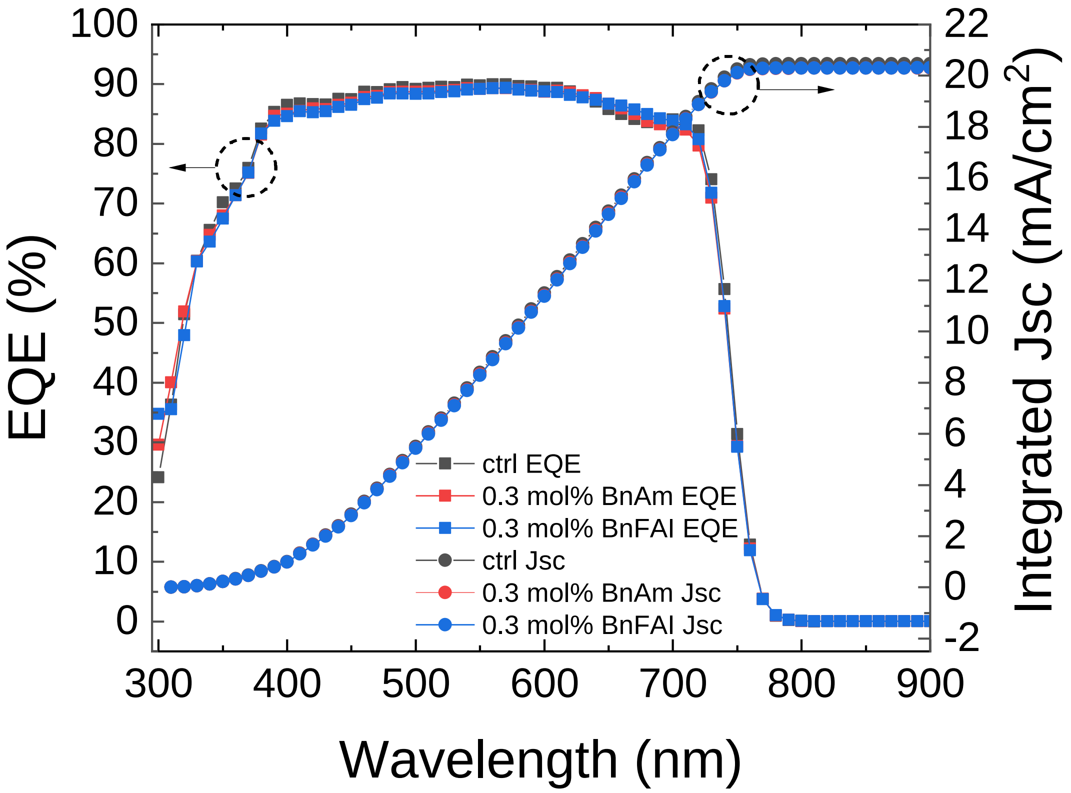

Supplementary Figure S43. p-i-n $FA_{0.75}Cs_{0.25}Pb(I_{0.8}Br_{0.2})_3$ devices external quantum efficiency (EQE) and integrated J_{sc} of 0.3 mol% BnFAI additive compared to 0.3 mol% BnAm additive. Device structure: FTO/Me4PACz/Al_2O_3 nanoparticles/$FA_{0.75}Cs_{0.25}Pb(I_{0.8}Br_{0.2})_3$/PCBM/BCP/Au.

To show that the benzylamine additive approach is applicable to different bandgaps and device configurations, we fabricated p-i-n devices: FTO/Poly-TPD/ $FA_{0.8}Cs_{0.2}PbI_3$/PCBM/BCP/Au (**Figure S38b**). We added BnAm at varying concentrations to a 1.55 eV $FA_{0.8}Cs_{0.2}PbI_3$ PSCs, suitable for single-junction PV applications (**Figure S44**). BnAm concentration was optimized at 0.2 mol%, reaching a champion reverse scan power conversion efficiency (PCE) of 18.8% compared to the control devices at 16.6% (**Figure S45**). After 30 s of MPP efficiency tracking, the device with 0.2 mol% BnAm stabilized at η_{MPP} of 17.4%, whereas the control device stabilized at 16.1%.

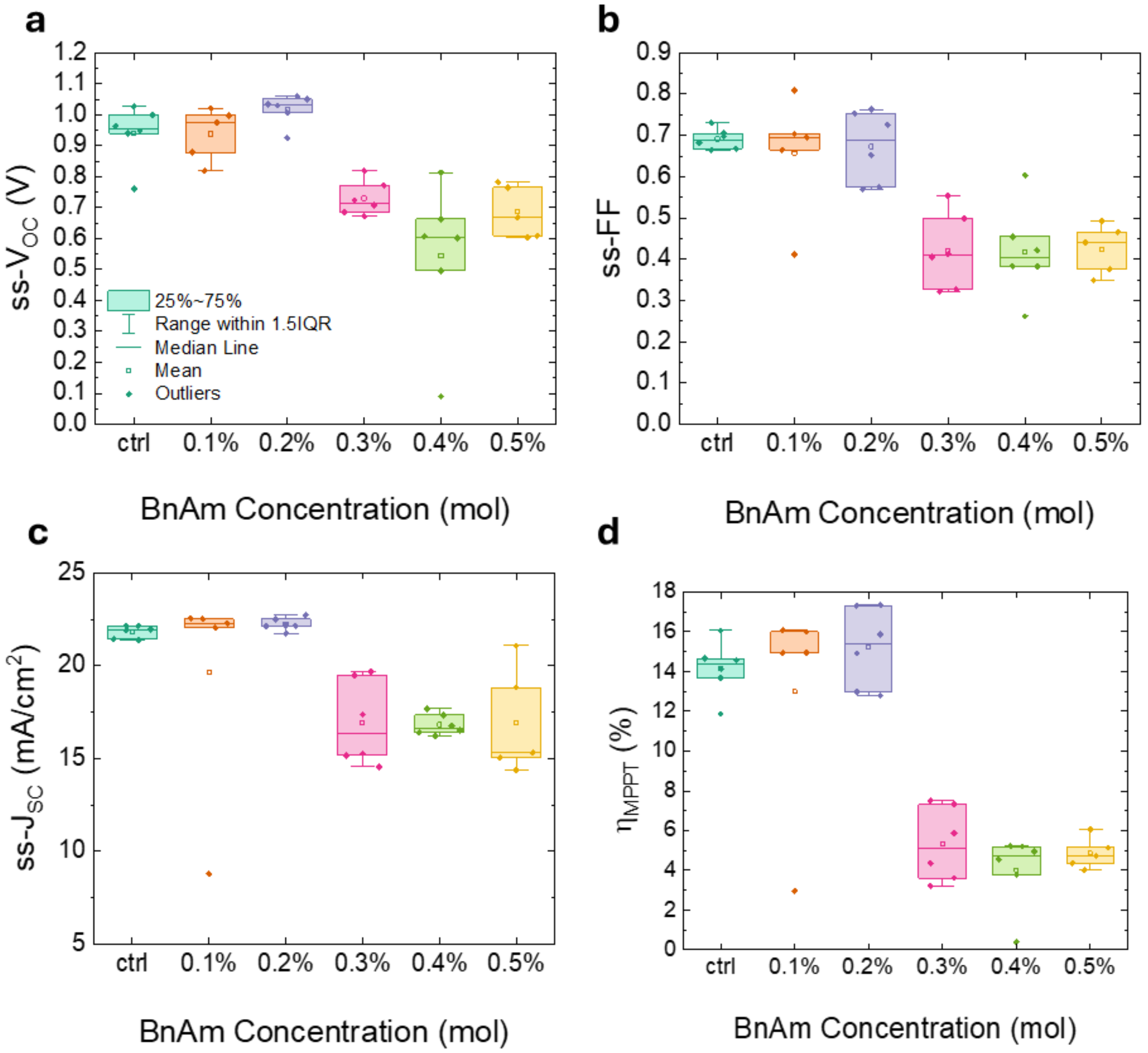

Supplementary Figure S44. Device parameters of 1.55 eV $FA_{0.8}Cs_{0.2}PbI_3$ p-i-n solar cells. (a) Steady-state open-circuit voltage stabilized for 30 seconds, (b) Steady-state fill factor stabilized for 30 seconds, (c) Steady-state short-circuit current density stabilized for 10 seconds, (d) Maximum power point tracked efficiency for 30 seconds.

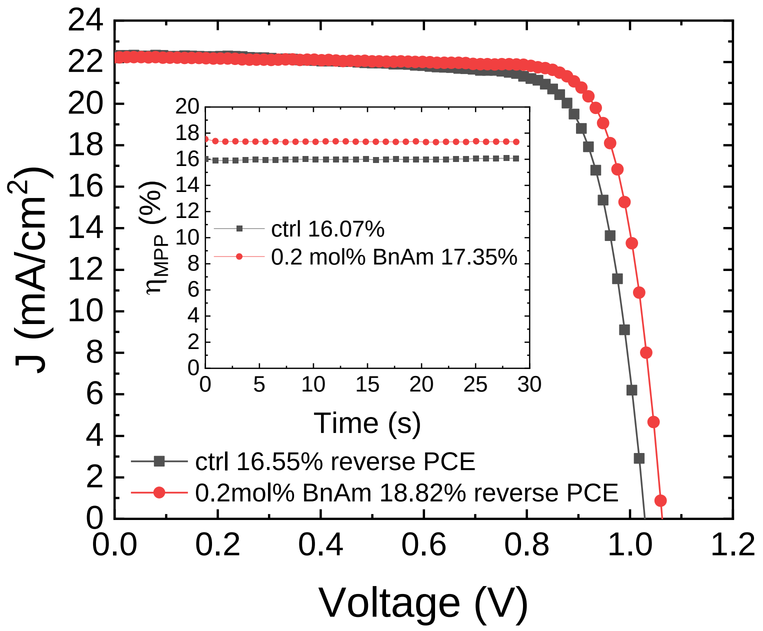

Supplementary Figure S45. Device J-V curves of champion 1.55 eV $FA_{0.8}Cs_{0.2}PbI_3$ p-i-n solar cells with 0.2 mol% BnAm addition and without BnAm addition.

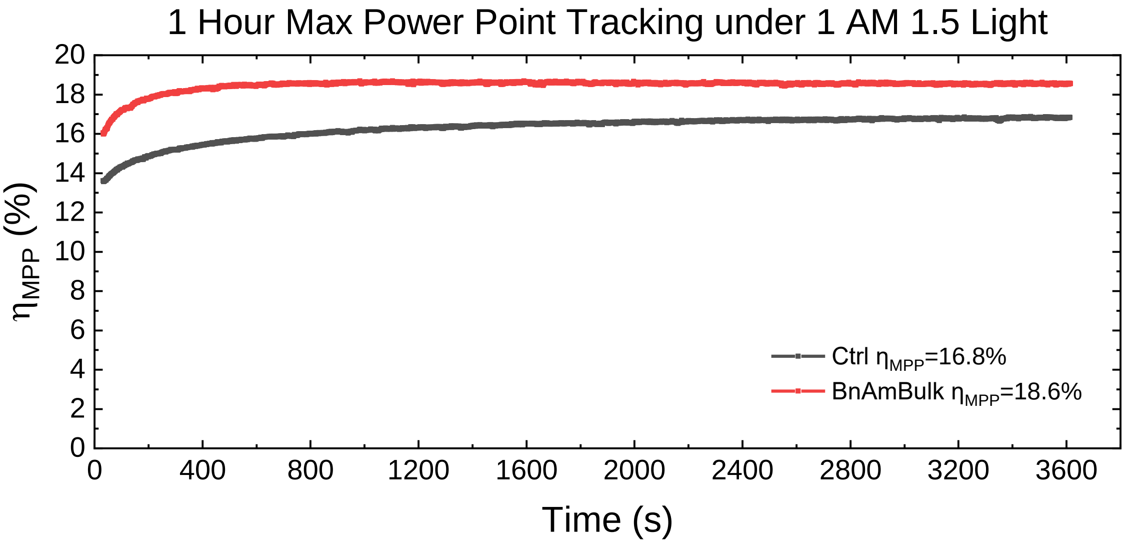

Supplementary Figure S46. 1 hour MPPT of champion 1.55 eV $FA_{0.8}Cs_{0.2}PbI_3$ p-i-n solar cells with 0.2 mol% BnAm addition and without BnAm addition.

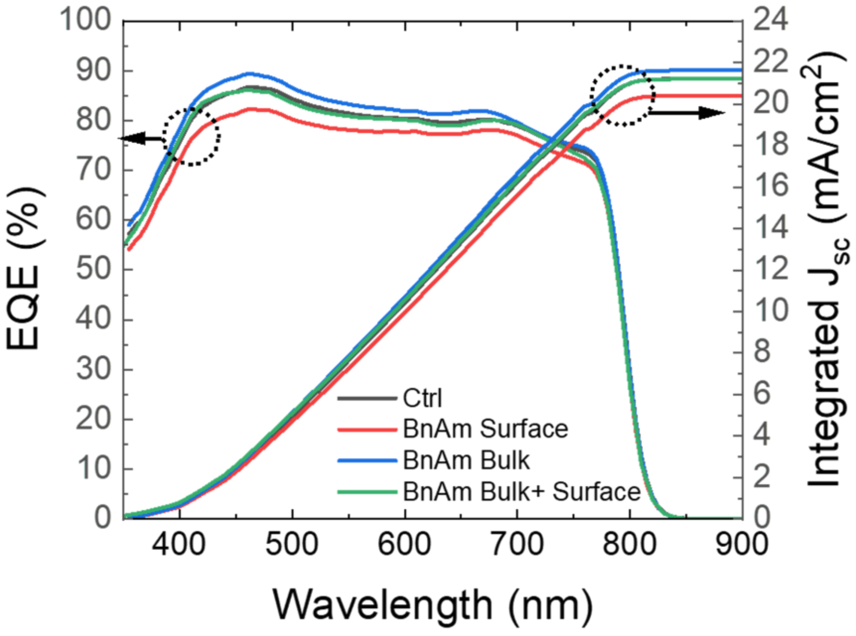

Supplementary Figure S47. EQE (left) and integrated current density (right) for p-i-n devices with $FA_{0.8}Cs_{0.2}PbI_3$ as the absorber. BnAm Surface is with 25 mM BnAm (in chlorobenzene) concentration surface treatment, BnAm Bulk is with 0.2 mol% BnAm additive. BnAm Bulk+ Surface is perovskite with 0.2 mol% BnAm additive and 25 mM BnAm surface treatment.

3.9. Perovskite Solar Cell Stability

BnAm surface passivation has been reported in the past[1,2] and there are ongoing discussions about 2D perovskite layer and domain-capping strategies.[13–16] We believe the BnAm passivation mechanism is a domain-capping effect, hence, to show that BnAm additive is a bulk passivation and that it is different from BnAm surface passivation, we also compare the stability of surface passivated and bulk passivated perovskite devices.

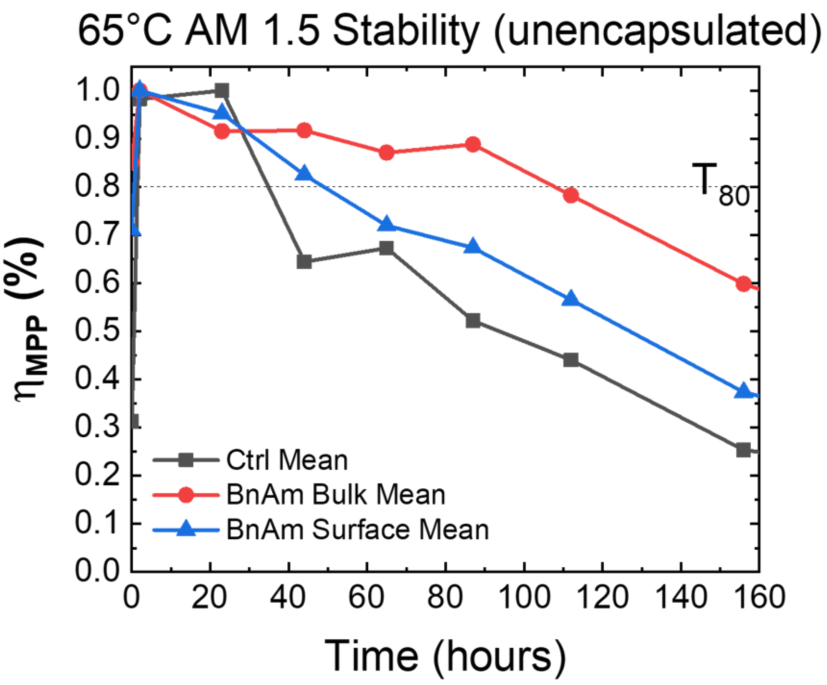

Supplementary Figure S48. 65 °C AM 1.5 intensity aging p-i-n devices with the architecture, FTO/Poly-TPD/$FA_{0.8}Cs_{0.2}PbI_3$/PCBM/BCP/Cr/Au (unencapsulated). BnAm bulk is with 0.2 mol% additive and BnAm surface is with 25 mM of surface passivation. Each data point is the average η_{MPP} of 5 devices for each condition. Devices were aged under open-circuit voltage conditions and taken out of the aging box regularly for testing.

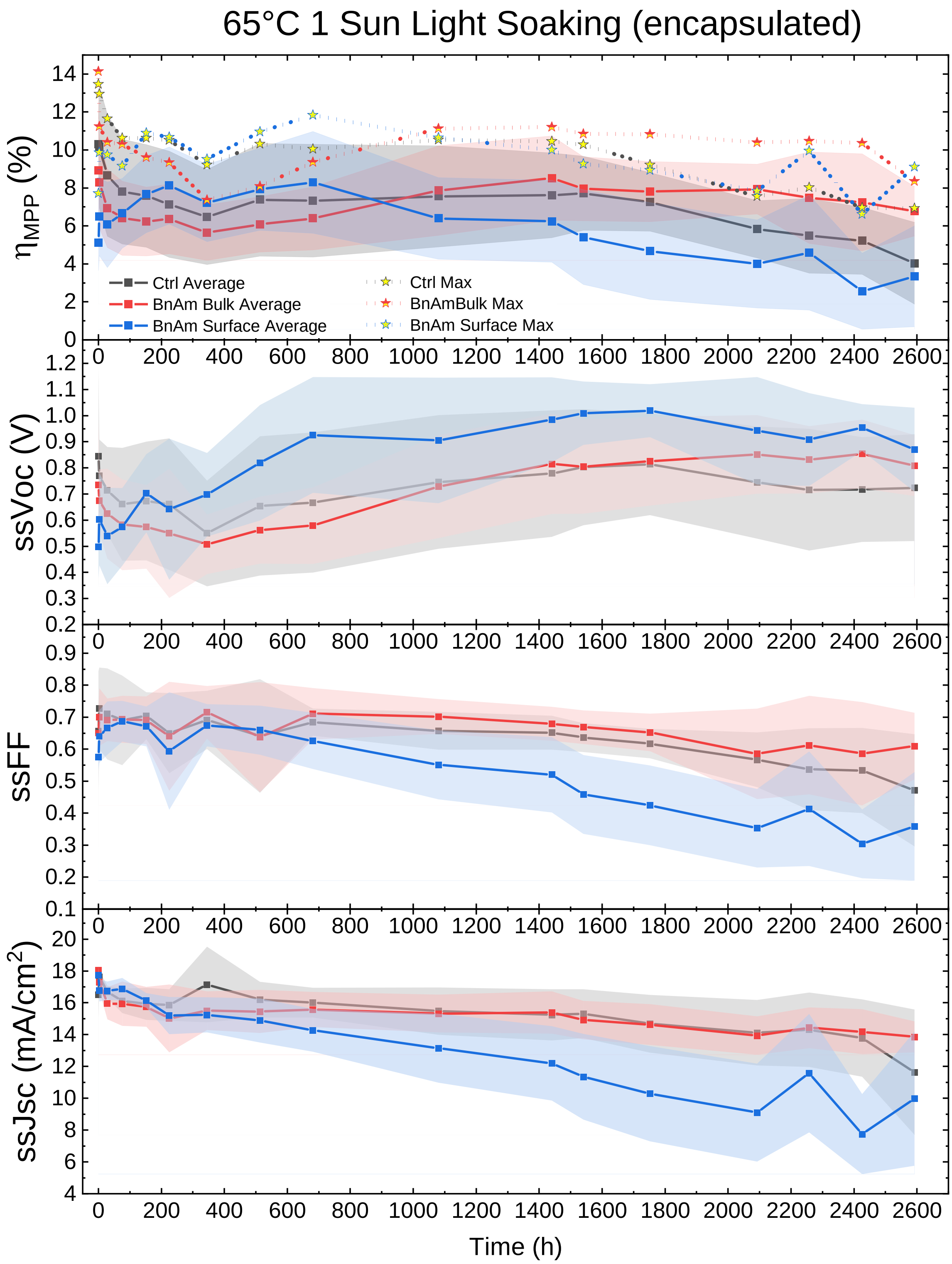

Supplementary Figure S49. 65 °C AM 1.5 intensity aging p-i-n devices with the architecture, FTO/Poly-TPD/$FA_{0.75}Cs_{0.25}Pb(I_{0.8}Br_{0.2})_3$/PCBM/BCP/Cr/Au (encapsulated). Individual device parameters degradation over time. Incidentally, this initial fast drop in efficiency followed by a slow recovery before a much slower long-term degradation at longer times is peculiar, but reproducible.

4. References

(1) Wang, F.; Geng, W.; Zhou, Y.; Fang, H.-H.; Tong, C.-J.; Antonietta Loi, M.; Liu, L.-M.; Zhao, N.; Wang, F.; Zhou, Y.; Zhao, N.; Geng, W.; Tong, C.; Liu, L.; Fang, H.; Loi, M. A. Phenylalkylamine Passivation of Organolead Halide Perovskites Enabling High-Efficiency and Air-Stable Photovoltaic Cells. *Adv. Mater.* **2016**, *28* (45), 9986–9992. https://doi.org/10.1002/ADMA.201603062.

(2) Zhou, Y.; Wang, F.; Cao, Y.; Wang, J.-P.; Fang, H.-H.; Loi, M. A.; Zhao, N.; Wong, C.-P. Benzylamine-Treated Wide-Bandgap Perovskite with High Thermal-Photostability and Photovoltaic Performance. *Adv. Energy Mater.* **2017**, *7* (22), 1701048. https://doi.org/https://doi.org/10.1002/aenm.201701048.

(3) de Mello, J. C.; Wittmann, H. F.; Friend, R. H. An Improved Experimental Determination of External Photoluminescence Quantum Efficiency. *Adv. Mater.* **1997**, *9* (3), 230–232. https://doi.org/10.1002/adma.19970090308.

(4) Kirchartz, T.; Márquez, J. A.; Stolterfoht, M.; Unold, T. Photoluminescence-Based Characterization of Halide Perovskites for Photovoltaics. *Adv. Energy Mater.* **2020**, *10* (26), 1904134. https://doi.org/10.1002/AENM.201904134.

(5) Dasgupta, A.; Mahesh, S.; Caprioglio, P.; Lin, Y.-H.; Zaininger, K.-A.; Oliver, R. D. J.; Holzhey, P.; Zhou, S.; McCarthy, M. M.; Smith, J. A. Visualizing Macroscopic Inhomogeneities in Perovskite Solar Cells. *ACS Energy Lett.* **2022**, *7* (7), 2311–2322.

(6) Ashiotis, G.; Deschildre, A.; Nawaz, Z.; Wright, J. P.; Karkoulis, D.; Picca, F. E.; Kieffer, J. The Fast Azimuthal Integration Python Library: PyFAI. *J. Appl. Crystallogr.* **2015**, *48* (2), 510–519.

(7) Munechika, K.; Chen, Y.; F. Tillack, A.; P. Kulkarni, A.; Jen-La Plante, I.; M. Munro, A.; S. Ginger, D. Quantum Dot/Plasmonic Nanoparticle Metachromophores with Quantum Yields That Vary with Excitation Wavelength. *Nano Lett.* **2011**, *11* (7), 2725–2730. https://doi.org/10.1021/nl2010127.

(8) Lindsey, C. P.; Patterson, G. D. Detailed Comparison of the Williams–Watts and Cole–Davidson Functions. *J. Chem. Phys.* **2008**, *73* (7), 3348–3357. https://doi.org/10.1063/1.440530.

(9) Kamminga, M. E.; De Wijs, G. A.; Havenith, R. W. A.; Blake, G. R.; Palstra, T. T. M. The Role of Connectivity on Electronic Properties of Lead Iodide Perovskite-Derived Compounds. *Inorg. Chem.* **2017**, *56* (14), 8408–8414. https://doi.org/10.1021/ACS.INORGCHEM.7B01096/ASSET/IMAGES/LARGE/IC-2017-010966_0005.JPEG.

(10) Breiby, D. W.; Bunk, O.; Andreasen, J. W.; Lemke, H. T.; Nielsen, M. M. Simulating X-Ray Diffraction of Textured Films. *J. Appl. Crystallogr.* **2008**, *41* (2), 262–271.

(11) Kainz, M. P.; Legenstein, L.; Holzer, V.; Hofer, S.; Kaltenegger, M.; Resel, R.; Simbrunner, J. GIDInd: An Automated Indexing Software for Grazing-Incidence X-Ray Diffraction Data. *J. Appl. Crystallogr.* **2021**, *54* (4), 1256–1267.

(12) Mao, L.; Ke, W.; Pedesseau, L.; Wu, Y.; Katan, C.; Even, J.; Wasielewski, M. R.; Stoumpos, C. C.; Kanatzidis, M. G. Hybrid Dion–Jacobson 2D Lead Iodide Perovskites. *J. Am. Chem. Soc.* **2018**, *140* (10), 3775–3783. https://doi.org/10.1021/jacs.8b00542.

(13) Sutanto, A. A.; Caprioglio, P.; Drigo, N.; Hofstetter, Y. J.; Garcia-Benito, I.; Queloz, V. I. E.; Neher, D.; Nazeeruddin, M. K.; Stolterfoht, M.; Vaynzof, Y.; Grancini, G. 2D/3D Perovskite Engineering Eliminates Interfacial Recombination Losses in Hybrid Perovskite Solar Cells. *Chem* **2021**, *7* (7), 1903–1916. https://doi.org/https://doi.org/10.1016/j.chempr.2021.04.002.

(14) Grancini, G.; Nazeeruddin, M. K. Dimensional Tailoring of Hybrid Perovskites for Photovoltaics. *Nat. Rev. Mater.* **2019**, *4* (1), 4–22. https://doi.org/10.1038/s41578-018-0065-0.

(15) Chen, J.; Yang, Y.; Dong, H.; Li, J.; Zhu, X.; Xu, J.; Pan, F.; Yuan, F.; Dai, J.; Jiao, B.; Hou, X.; Jen, A. K.-Y.; Wu, Z. Highly Efficient and Stable Perovskite Solar Cells Enabled by Low-Dimensional Perovskitoids. *Sci. Adv.* **2024**, *8* (4), eabk2722. https://doi.org/10.1126/sciadv.abk2722.

(16) Leung, T. L.; Ahmad, I.; Syed, A. A.; Ng, A. M. C.; Popović, J.; Djurišić, A. B. Stability of 2D and Quasi-2D Perovskite Materials and Devices. *Commun. Mater.* **2022**, *3* (1), 63. https://doi.org/10.1038/s43246-022-00285-9.
